# Cationic Amphipathic Triazines with Potent Anti-bacterial, Anti-inflammatory and Anti-atopic Dermatitis Properties

**DOI:** 10.1038/s41598-018-37785-z

**Published:** 2019-02-04

**Authors:** Pethaiah Gunasekaran, Ganesan Rajasekaran, Eun Hee Han, Young-Ho Chung, Young-Jin Choi, Yu Jin Yang, Ji Eun Lee, Hak Nam Kim, Kiram Lee, Jin-Seok Kim, Hyun-Jun Lee, Eun-Ju Choi, Eun-Kyung Kim, Song Yub Shin, Jeong Kyu Bang

**Affiliations:** 1 Division of Magnetic Resonance, Korea Basic Science Institute (KBSI), Ochang, Chung Buk 28119 Republic of Korea; 2 Department of Cellular and Molecular Medicine, Chosun University, Gwangju, 501-759 Republic of Korea; 3 Drug & Disease Target Research Team, Korea Basic Science Institute (KBSI), Cheongju, 28119 Republic of Korea; 4 Division of Food Bioscience, Konkuk University, Chungju, 27478 Republic of Korea; 5 Natural Medicine Research Center, Korea Research Institute of Bioscience and Biotechnology, Ochang-eup, Chungcheongbuk-do 28116 Republic of Korea; 6 Department of Physical Education, Daegu Catholic University, Gyeongsan, 38430 Republic of Korea; 7 Department of Bio-analytical Science, University of Science & Technology, Daejeon, 34113 Republic of Korea

## Abstract

The emergence of multi-drug resistant bacteria forces the therapeutic world into a position, where the development of new and alternative kind of antibiotics is highly important. Herein, we report the development of triazine-based amphiphilic small molecular antibacterial agents as mimics of lysine- and arginine-based cationic peptide antibiotics (CPAs). These compounds were screened against a panel of both Gram-positive and Gram-negative bacterial strains. Further, anti-inflammatory evaluation of these compounds led to the identification of four efficient compounds, DG-5, DG-6, DL-5, and DL-6. These compounds displayed significant potency against drug-resistant bacteria, including methicillin-resistant *S*. *aureus* (MRSA), multidrug-resistant *P*. *aeruginosa* (MDRPA), and vancomycin-resistant *E*. *faecium* (VREF). Mechanistic studies, including cytoplasmic membrane depolarization, confocal imaging and flow cytometry suggest that DG-5, DG-6, and DL-5 kill bacteria by targeting bacterial membrane, while DL-6 follows intracellular targeting mechanism. We also demonstrate that these molecules have therapeutic potential by showing the efficiency of DG-5 in preventing the lung inflammation of lipopolysaccharide (LPS)-induced acute lung injury (ALI) mouse model. More interestingly, DL-6 exhibited impressive potency on atopic dermatitis (AD)-like skin lesions in BALB/c mice model by suppressing pro-inflammatory cytokines. Collectively, these results suggest that they can serve a new class of antimicrobial, anti-inflammatory and anti-atopic agents with promising therapeutic potential.

## Introduction

Since the discovery of penicillin in the 1920s, a number of antibiotics have been introduced in the market to treat infectious diseases. However, in recent decades, pharmaceutical companies limited their investments for the discovery of new antibiotics because of reduced profits and short life-span of antibiotics^1^. Still, the frequent and excessive misuse of antibiotics leads to the inexorable emergence of drug-resistant pathogens, namely superbugs that are a serious and imminent threat to antibiotic therapy^2^. Moreover, the accompanying rise of antibiotic resistance is considered a serious and concerning challenge for the pharmaceutical industries, country leaders, and the public because of the social and economic burden imposed by it in treating diseases. Thus, a rapid increase in multidrug-resistant (MDR) pathogens and an apparent dearth of a new class of antibiotics necessitate the discovery of alternative and potent antimicrobial agents^3–5^. In this regard, cationic peptide antibiotics (CPAs), also known as cationic antimicrobial peptides, find importance as a new generation of antibiotics in treating drug-resistant pathogens owing to their distinctive mode of action and rapid killing rate^6,7^. CPAs have drawn remarkable attention because they are widely found in species ranging from bacteria to mammals and exhibit potent antimicrobial activities^8,9^. They exist in both *α*-helical and *β*-sheet forms, which exhibit amphiphilic conformations by presenting the cationic groups on one side and hydrophobic groups on the other side of the molecules that result in cationic/hydrophobic segregations^10,11^. They exhibit selectivity to prokaryotic cells over eukaryotic cells because CPAs act on the negatively charged bacterial membrane by the effective association of their positive groups in the amphiphilic structure and also by forming stable pores, thus disrupting the membrane^12^. Consequently, the bacterial killing is very rapid and bacterial resistance is limited; they can also permeate the outer membranes of Gram-negative bacteria, and exhibit a broad spectrum of antibacterial activity^13^. Despite CPAs being an alternative for treating drug-resistant bacteria, their transformation into molecules of therapeutic use is a highly daunting task owing to several disadvantages, including their large size, hemolytic activity, proteolytic instability, poor salt resistance, and cytotoxicity^14^. Hence, amphiphilic small molecules containing a lipophilic groups and cationic groups exhibiting controlled hemolytic activity are highly imperative for the discovery of new antibiotics and to overcome peptide-associated disadvantages, including proteolytic instability and poor cell permeability^15,16^. Thus, small molecules with structural features of CPAs are considered as a prominent strategy that may help in the discovery of small-molecule antibacterials. To accomplish this, we synthesized *s*-triazine-based cationic amphipathic small molecules comprising cationic groups including amines and guanidyl groups as mimetics of lysine and arginine-containing CPAs, and hydrophobic residues, yielding amphiphilic small molecules containing both hydrophobic and hydrophilic groups.

Over the years, synthesis of *s*-triazine-based heterocycles have received considerable interest in the field of medicinal chemistry, due to their remarkable pharmacological properties and easy access to array the structural diversity. *s*-Triazine derivatives were found to exhibit diverse biological properties such as anticancer^17^, antiviral^18^, antiprotozoal^19^, antimalarial^20^, estrogen receptor modulatory^21^, cyclin-dependent kinase inhibitory^22^, and antifungal activities^23^. Moreover, *s*-triazine derivatives were found to show antibacterial activities^24,25^; however, to the best of our knowledge, a detailed mechanistic study on CPA-mimicking amphipathic triazine analogues with profound anti-inflammatory and anti-atopic properties has not been reported yet. The present work stems as a part of our research program embarked on the discovery of new antibacterial agents^26–28^.

In this work, we have designed and synthesized a series of triazine-based small-molecule antibacterials containing a norspermidine (**1**), which is abundantly available and inexpensive, and has at least two positive charges. Moreover, both mono and bis(alkyls/alkylaryls) were anchored to achieve hydrophobicity. The resultant amphipathic structure provides insights into opportunities for finding the key factors, which are essential for displaying antibacterial activity. These small molecules were assayed for their antibacterial activity against both Gram-positive and Gram-negative bacterial strains. With an aim of identifying the most promising compound as a potential therapeutic lead, the compounds with the highest antibacterial activity and low hemolytic activity, including **DL-5**, **DL-6**, **DG-5**, and **DG-6**, were evaluated for their activity against MRSA, MDRPA, and VREF. Moreover, most of the compounds were examined for their cytotoxicity using sheep red blood cells (sRBCs) and RAW 264.7 cells. In order to understand the bacterial killing mechanism and to identify the effect of these compounds on the integrity of the bacterial membranes and intracellular region, we performed cytoplasmic membrane depolarization experiments and confocal imaging studies. We investigated the inhibition potency of **DG-5**, **DG-6**, **DL-5**, and **DL-6** in LPS-induced inflammatory responses *in vitro*. In addition, the *in vivo* activity of **DG-5** against LPS-induced inflammatory responses in a mouse model of LPS-induced ALI (acute lung injury) was evaluated. Finally, inspired by the proficient anti-inflammatory effect of **DL-6**, our synthesized small-molecule antibacterial compound, **DL-6** has been investigated for its effect on AD-like skin lesions. Its underlying mechanisms of action was studied using BALB/c model by measuring the ear thickness in mice with AD-like skin lesions and analyzing the histopathological changes including mast cell count and cytokine expression in ear tissue.

## Results and Discussion

### Synthesis of triazine-based antibacterials

**Figure 1:**
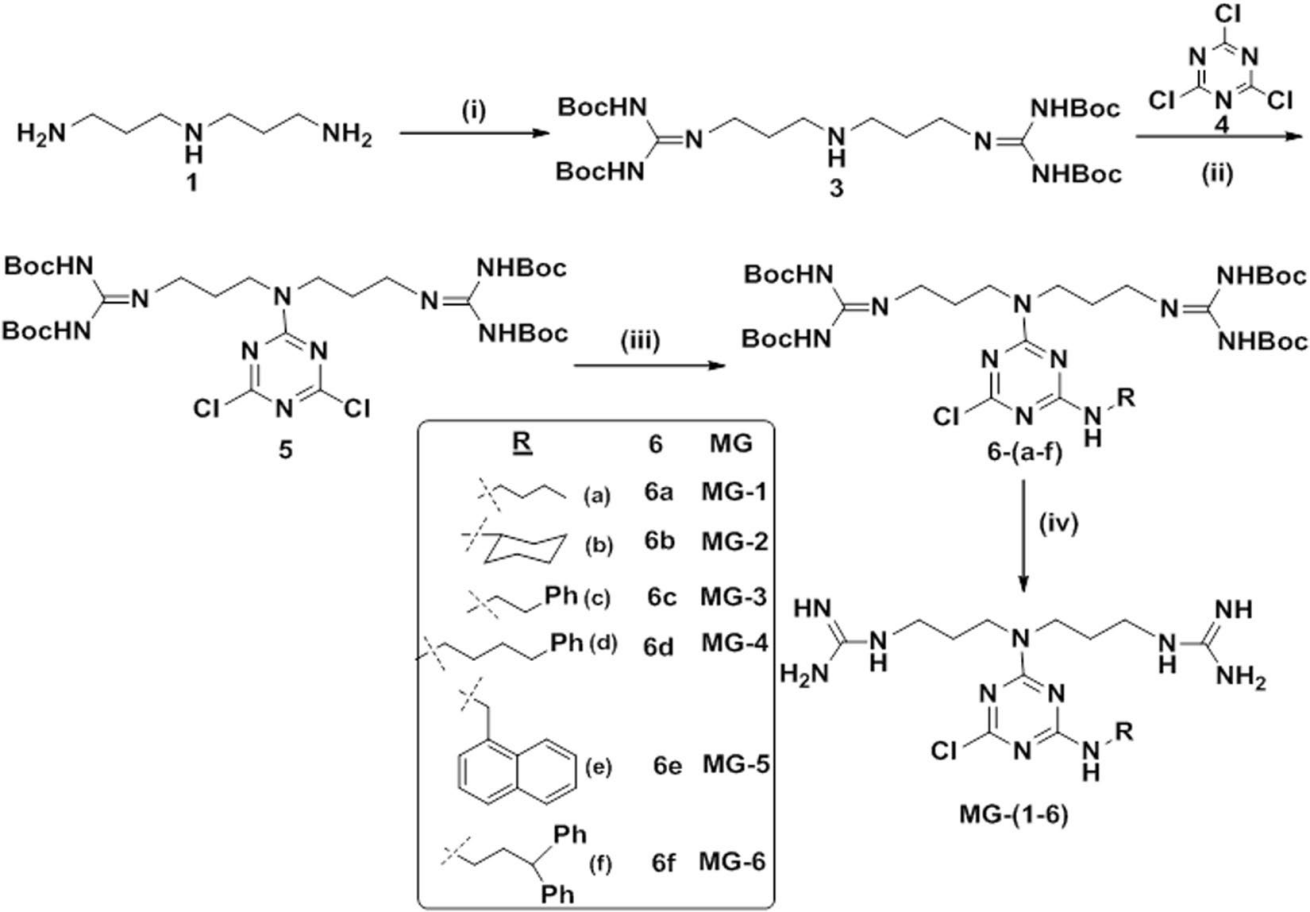
Synthesis of **MG-(1–6)**, reagents and conditions: (i) *N*,*N*′-di-Boc-*N*″-trifylguanidine (**2**), TEA, DCM, rt, 6 h, 68%; (ii) cyanuric chloride (**4**), DIEA, DCM, 0 °C, 4 h, 97%; (iii) R-NH_2_, DIEA, DCM, rt, 4 h; (iv) TFA/DCM (3:1), 0 °C - rt, 3 h.

It is reported that the 2,4,6-positioned chlorine atoms in 2,4,6-trichloro-1,3,5-triazine, i.e., cyanuric chloride, are more susceptible to the nucleophilic substitution reactions, which offers the synthetic accessibility to substitute diverse nucleophiles sequentially by controlling the temperature gradient to achieve diversity in the structure^25^. To synthesize a series of compounds to perform the structure activity relationship study, we introduced norspermidine derivatives comprising amine or guanidyl groups and various alkyl/arylalkyl groups into *s*-triazine by replacing the chlorine atoms in 2,4,6-trichloro-1,3,5-triazine using the variable temperature strategy. Initially, the guanidinylation at the terminal amines of norspermidine (**1**) was carried out using *N*,*N*′-di-Boc-*N*″-trifylguanidine (**2**) to achieve **3**, which was successfully adopted for the nucleophilic substitution reaction with 2,4,6-trichloro-1,3,5-triazine (**4**) in basic condition to result in the formation of **5** as delineated in Fig. 1. In order to incorporate the hydrophobic groups, various amines were treated with **5** in the presence of base at room temperature to achieve (**6**). Finally, the multiple Boc groups on the guanidyl groups were successfully removed using TFA and subsequent reverse phase HPLC purification resulted in the formation of **MG-(1–6)**.

**Figure 2:**
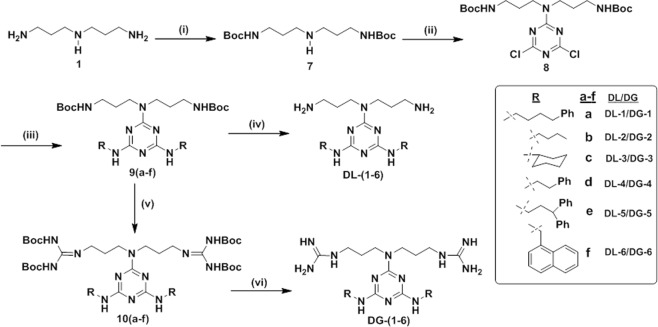
Synthesis of **DG-(1–6)**/**DL-(1–6)**, reagents and conditions**:** (i) Boc-ON, DIEA, THF, 0 °C - rt, 21 h, 80%; (ii) cyanuric chloride (**4**), DIEA, DCM, 0 °C, 3 h, 87%; (iii) R-NH_2_, DIEA, 1,4–dioxane, reflux, 14 h; (iv) TFA/DCM (3:1), 0 °C - rt, 3 h; (v) (**a**) TFA/DCM (3:1), 0 °C - rt, 3 h, (**b**) *N*,*N*′-di-Boc-*N*′-trifylguanidine (**2**), TEA, DCM, rt; (vi) TFA/DCM (3:1), 0 ^o^C - rt, 3 h.

Then, to diversify the analogs to perform the SAR studies, the **DL** and **DG** series of compounds were synthesized as shown in Fig. 2. We began our synthesis with the selective Boc protection at the primary amines of norspermidine (**1**) to obtain **7**. Treatment of **7** with cyanuric chloride in dichloromethane (DCM) in the presence of diisopropylethylamine (DIEA) at low temperature resulted in the formation of **8**. In order to introduce various hydrophobic groups at the expense of replacing two chlorine atoms by nucleophilic substitution reactions, various amines were treated with **8** in the presence of DIEA in 1,4-dioxane at reflux conditions, which resulted in the formation of various derivatives, **9(a–f)**. Finally, treatment of **9(a–f)** with TFA in DCM yielded the desired compounds, **DL-(1–6)**. Next, in view of introducing guanidine groups to attain the protected arginine mimic **10(a–f)**, the series of compounds **9(a–f)** was treated with TFA in DCM, resulting in the deprotection of two Boc groups and subsequent treatment of triethylamine and *N*,*N*′-di-Boc-*N*″-trifylguanidine resulted in the formation of compounds, **10(a–f)** with appreciable yields^29^. Finally, treatment with TFA in DCM followed by reverse phase HPLC purification yielded **DG-(1–6)**.

### Structure-antibacterial activity study

The antibacterial activity of the **MG**, **DG**, and **DL** series compounds was investigated and their minimum inhibitory concentration (MIC) values determined using a panel of four organisms representative of both two Gram-negative bacteria [(*E*. *coli* (KCTC 1682) and *P*. *aeruginosa* (KCTC 1637)], and two Gram-positive bacteria [(*S*. *epidermidis* (KCTC 1917) and *S*. *aureus* (KCTC 1621)].

We began our structure activity study by synthesizing the **MG** series of compounds in the first phase of derivatization as shown Table 1. Initially, we introduced two hydrophobic residues including a linear alkyl (*n*-butyl (**MG-1**)) and a cyclic alkyl (cyclohexyl (**MG-2**)) residue by replacing a chlorine atom in the core residue of **5**. Unfortunately, the MIC was not appreciable against any bacterial strain except *S*. *aureus*, where both compounds showed minimal activity. These results suggest that both compounds lacked hydrophobicity. In order to increase the hydrophobicity, ethylphenyl and butylphenyl were incorporated, which yielded **MG-3** and **MG-4**, respectively. Interestingly, **MG-3** and **MG-4** showed improved results against *P*. *aeruginosa* and *S*. *aureus*. In particular, **MG-4** showed better results against *P*. *aeruginosa*, *S*. *epidermidis*, and *S*. *aureus*, compared to **MG-3**, which demonstrated that further increase in hydrophobicity would result in a rise in antibacterial activity. To verify this hypothesis, we further enhanced the hydrophobicity by introducing naphthyl methyl (**MG-5**) and 3,3-diphenylpropyl (**MG-6**) residues. Although **MG-5** did not show an appreciable antibacterial activity, **MG-6** displayed better activity against almost all the strains compared to the rest of the compounds in the **MG** series. Collectively, the antibacterial effect of these compounds followed an order of 3,3-diphenylpropyl **(MG-6)** > butylphenyl **(MG-4)** > ethylphenyl (**MG-3**) > naphthylmethyl (**MG-5)** > cyclohexyl **(MG-2)** and *n*-butyl (**MG-1**) value.

**Table 1:** Antibacterial and hemolytic activities of **MG**, **DG** and **DL** compounds.

Compound	MIC^a^ (μM)						
	Gram-negative bacteria		Gram-positive bacteria				
	*E*. *coli* (KCTC 1682)	*P*. *aeruginosa* (KCTC 1637)	*S*. *epidermidis* (KCTC 1917)	*S*. *aureus* (KCTC 1621)	GM(μM)^b^	MHC (μM)^c^	TI (MHC/GM)^d^
First phase derivatisation							
MG-1	>160	80	>160	40	190	>320	16.0
MG-2	>160	80	>160	20	185	>320	3.5
MG-3	160	40	160	10	92.5	>320	6.9
MG-4	160	20	80	5	66.3	>320	9.7
MG-5	160	160	160	5	121.3	>320	5.3
MG-6	40	10	40	5	23.8	>320	26.9
Second phase derivatisation							
DG-1	5	10	10	2.5	6.9	177	25.7
DG-2	160	20	160	10	87.5	>320	7.3
DG-3	10	10	10	2.5	8.1	>320	79.0
DG-4	40	10	20	2.5	18.1	>320	35.4
DG-5	5	5	10	2.5	5.6	140	25.0
DG-6	5	10	10	2.5	6.9	88	12.8
DL-1	20	10	10	2.5	10.6	88	8.3
DL-2	>160	10	>160	80	182.5	>320	3.5
DL-3	160	10	160	10	85.0	>320	7.5
DL-4	160	20	160	10	87.5	>320	7.3
DL-5	5	5	5	2.5	4.4	185	42.0
DL-6	5	10	20	2.5	9.4	>320	68.1
melittin	2.5	5	5	2.5	3.8	4	1.1

^a^MICs of the synthesized compounds, are given in µM, were determined as the lowest concentration of compounds which requires to inhibit 100% microbial growth. ^b^GM is the geometric mean of MICs of tested bacterial strains. ^c^MHC is the lowest concentration of compounds, which require to induce 10% hemolysis in sheep red blood cells. ^d^TI (therapeutic index) is the ratio between MHC value (µM) and GM (µM). 320 µM was accounted for calculating the GM value when MIC was observed more than160 µM. 640 µM was considered for calculating the MHC value when 10% hemolysis was not found until 320 µM.

### Second phase derivatization

It is pertinent to note that the balance between the charge density and the hydrophobicity is one of the key determinants of the antibacterial activity^30^. To investigate this, initially, **DG-1** was synthesized by the incorporation of two butylphenyl residues and screened against all the four bacterial strains. Interestingly, the MIC value of **DG-1** was significant against all the strains; in particular, the antibacterial efficiency against *S*. *aureus* (2.5 µM) was the same as that of melittin. These results suggested that two butylphenyl residues balanced the charge produced by two guanidyl residues, compared with the mono butylphenyl substituted **MG-4**. This inference was further ascertained by decreasing the hydrophobicity from bis(butylphenyl) to bis(*n*-butyl) (**DG-2**), which ultimately resulted in dropping its antibacterial activity against almost all the strains compared to **DG-1**. Substitution of the symmetrical bis(cyclohexyl) residues (**DG-3**) showed an antibacterial effect nearly as equal as that of **DG-1** with enhanced therapeutic index (TI) values. In general, the therapeutic potential of antibacterial agents represents their selectivity towards killing the bacterial cells without displaying significant cytotoxicity to mammalian cells. Thus, cell selectivity of the synthesized compounds is known as therapeutic index (TI) = minimum hemolytic concentration (MHC)/geometric mean (GM), where MHC denotes the antibacterial agent concentration required to cause the 10% lysis to human red blood cells, while GM is the geometric mean of MICs against four bacterial strains.

Hence, the relative safety and the proficiency of a drug candidate to differentiate between a pathogen and the host cells can be inferred from the therapeutic index (TI) values. Thus, larger TI values indicate greater cell selectivity. However, substituting **DG-4** with bis(ethylphenyl) displayed little decrease in antibacterial activity compared to **DG-3**. From the above results, it was observed that the increase in hydrophobicity could elevate the antibacterial activity. Therefore, elevated hydrophobicity was established by synthesizing **DG-5**, which comprises two 3,3-diphenylpropyl groups. Predictably, **DG-5** displayed potent antibacterial effect as almost similar to the control melittin, except against *E*. *coli* and *S*. *epidermidis*, where it elicited a two-fold reduced activity, while displaying significant TI value. Finally, **DG-6** was synthesized and investigated for its antibacterial efficacy by incorporating bis(naphthyl) residues as hydrophobic groups. Expectedly, **DG-6** also displayed an antibacterial efficiency as potent as **DG-5** and the control melittin, while there was a slight drop in MIC value against *P*. *aeruginosa* (10 µM). On the whole, based on the antibacterial activities with enhanced TI values, we identified four potent compounds namely, **DG-5**, **DG-6**, **DG-1**, and **DG-3**.

Next, to investigate the effect of changing the charge from bis(guanidyl) groups to bis(amino) groups while manipulating the hydrophobic residues in a similar way as in the **DG** series, the **DL** series of compounds were derivatized as delineated in Fig. 2. First, we began with anchoring the symmetrical bis(butylphenyl) residue against positively charged two amine residues that resulted in **DL-1**, which displayed antibacterial effects almost as same as **DG-1**. However, again, manipulating the hydrophobic residues, including *n*-butyl (**DL-2**), cyclohexyl (**DL-3**) and ethyl phenyl (**DL-4**) resulted in a loss of the antibacterial effect against *E*. *coli* and *S*. *epidermidis*, while showing appreciable activity against *P*. *aeruginosa* and *S*. *aureus* compared to the control. The decreasing activity can be attributed to the imbalance of the charge to hydrophobicity ratio. To investigate this, we synthesized **DL-5** by incorporating bis(3,3-diphenylpropyl). Surprisingly, **DL-5** displayed excellent antibacterial activity, being as effective as the control melittin, with significant TI value. This observation led to the identification of **DL-5** as the most effective compound among the **MG**, **DG**, and **DL** series of compounds. Further, **DL-6** was also synthesized and assayed against all the bacterial strains, and was found to display as potent an antibacterial activity as **DL-5**; while there was a little decrease in the efficacies against *P*. *aeruginosa* and *S*. *epidermidis*, and it showed an excellent TI value. Lastly, **DL-1**, **DL-5** and **DL-6** were the most promising compounds in the **DL** series of compounds. Our structure activity relationship studies inferred that (i). Triazine scaffold is an excellent choice for the design of new antibacterial agents; (ii). Both guanidine and amine can be used as positive charge donor; (iii). Bulky hydrophobic residues including 3,3-diphenylpropyl and 1-naphthylmethyl are necessary to balance the charge deduced from either guanidyl or amine groups. Finally, **DG-1**, **DG-5**, **DG-6**, **DL-1**, **DL-5**, and **DL-6** were spotted as most potent compounds based on their antimicrobial effects and TI values.

### Cell selectivity and cytotoxicity

**Figure 3:**
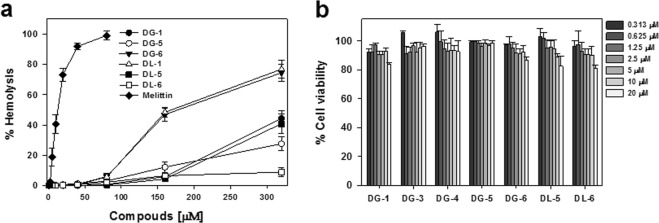
(**a**) Hemolytic activity of the selected compounds in sheep red blood cells (s-RBCs); (**b**) Cytotoxic evaluation of the selected compounds in mouse macrophage RAW264.7 cells.

Achieving maximum possible antibacterial effect with minimum toxicity towards the host is an attractive strategy for discovering antibacterial agents. The compounds with the most potent antibacterial activity, including **DG-1**, **DG-5**, **DG-6**, **DL-1**, **DL-5**, and **DL-6**, were evaluated for their hemolytic activity against sheep red blood cells in the range of 0–320 μM (Fig. 3a) to assess their ability in treating the bacterial cells selectively. **DG-1**, **DG-5**, **DG-6**, **DL-1**, and **DL-5** showed a noticeable hemolytic activity at 320 μM, the highest concentration tested (Fig. 3a and Table 1).

Interestingly, all these compounds exhibited less than 6% hemolysis until the concentration of 80 μM, at which the reference compound melittin almost induced 100% hemolysis. Even though **DL-1** and **DG-6** showed 50% hemolysis at a high concentration of 160 μM, the **DG-1**, **DL-5** and **DL-6** induced less than 8% hemolysis at the tested concentration, 160 μM. Astonishingly, the same lack of hemolysis was maintained even at more than 320 μM by **DL-6**, which was also revealed by its elevated TI values. **DG-5** and **DL-5** also showed appreciable cell selectivity by inducing less than 40% hemolytic even at a high concentration (more than 320 μM). These results further support the potential of these compounds for use as antibacterial therapeutics.

To further verify the cytotoxicity of **DL-5**, **DL-6**, **DG-5**, and **DG-6** against other mammalian cells, mouse macrophage RAW264.7 cells were used. Interestingly, these compounds didn’t show noticeable cytotoxicity until 20 μM as shown in Fig. 3b, despite the fact that amphipathic moieties are often found to exhibit cytotoxicity. The low toxicity and potent antibacterial activity of these compounds suggest that these compounds may have more affinity for negatively charged bacterial membrane than for zwitterionic mammalian membranes. Together these results indicate that these compounds could potentially be model compounds for the design of antibacterial therapeutic.

### Anti-inflammatory (endotoxin-neutralizing) activity

**Figure 4:**
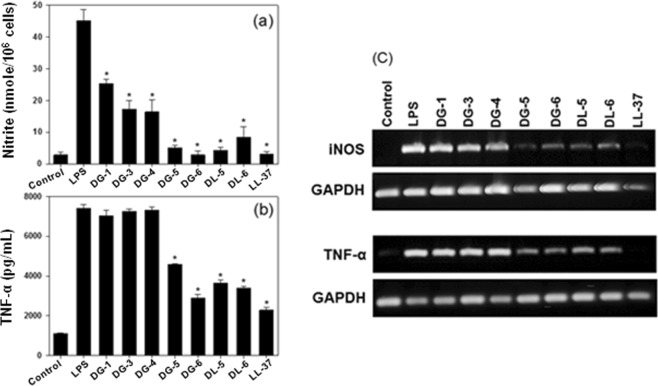
(**a**) Inhibition of compounds (10 μM) against nitric oxide (NO) production in LPS-stimulated RAW264.7 cells; (**b**) Inhibition of compounds (20 μM) on LPS-stimulated TNF-*α* release from RAW264.7 cells (Inhibition of compounds (10 μM) on TNF-*α* production is given in Fig. S2). Asterisks dictate the significant effects of the tested compounds compared to that of LPS treated cells. One-way ANOVA with Bonferroni’s post-test (*p < 0.001 for each agonist) used for analyzing data. The data are mean ± SEM of three experiments. The results remained similar when experiments were performed using different cells. Control indicates the cell only. (**c**) Anti-inflammatory efficacies of the tested compounds on suppressing the mRNA expression of iNOS and TNF-*α* in LPS-stimulated RAW264.7 cells by RT-PCR. *(Full-length blots are given in Supplementary information*).

In most of the Gram-negative bacteria, the outer membranes are composed of lipopolysaccharides (LPS, endotoxin) as a major constituent. During infection, the release of LPS from bacteria is inevitable, and consequently, monocytic and phagocytic cells are induced enormously to produce pro-inflammatory cytokines such as TNF-*α*, IL-6, IL-b, and others. Overexpression of cytokines have been associated with the development of sepsis and septic shock, leading to multiple organs damages^31,32^. Hence, it is worth discovering an antibacterial agent that has dual functions such as anti-microbial and anti-inflammatory effects to treat the bacteria-induced inflammation^33,34^. We examined seven compounds (**DG-1**, **DG-3**, **DG-4**, **DG-5**, **DG-6**, **DL-5**, and **DL-6**) with enhanced antibacterial activity and TI values to test their endotoxin-neutralizing effect using NO production and TNF-*α* release in LPS-stimulated RAW264.7 cells, because NO and TNF-*α* are two major causes promoting inflammatory responses (Fig. 4). As shown in Fig. 4a, all the seven compounds tested were found to inhibit LPS-induced NO production. In particular, **DG-5**, **DG-6**, **DL-5**, and **DL-6** were found to be more proficient in inhibiting NO production than the reference LL-37, which is well known for its characteristic LPS-neutralizing effects. Moreover, **DG-6** showed the maximum inhibitory potential with a similar spectrum as the reference and control compounds.

Then as shown in Fig. 4b, **DG-5**, **DG-6**, **DL-5**, and **DL-6** significantly inhibited TNF-*α* and the other compounds were found to show poor efficiency. In this study, **DG-6** showed the maximum TNF-*α* inhibition almost as similar as the reference compound, LL-37. This inference is in accordance with the results obtained for NO inhibition test, which confirms that **DG-5**, **DG-6**, **DL-5**, and **DL-6** possess significantly potent LPS-neutralizing potency to become model compounds for the future design of antibacterials with profound anti-inflammatory activity.

Usually, mRNA expression of inducible nitric oxide synthase (iNOS) and TNF-*α* in LPS-stimulated RAW264.7 cells are induced by LPS and that correlates with NO and TNF-*α* release. Thus, the ability of the synthesized compounds on suppressing the LPS-induced mRNA expressions of iNOS and TNF-*α* was investigated by RT-PCR as shown in Fig. 4c. Among the seven compounds tested, **DL-5**, **DL-6**, **DG-5**, and **DG-6** significantly reduced the expression of both iNOS and TNF-*α* in LPS-stimulated RAW264.7 cells. In particular, **DG-5** displayed potency almost similar compared with the reference compound, LL-37. Thus, this result also correlated with the results obtained for NO inhibition and TNF-*α* release, additionally, confirming that **DL-5**, **DL-6**, **DG-5**, and **DG-6** have potent anti-inflammatory activity.

### Antibacterial activity of DG-5, DG-6, DL-5 and DL-6 against antibiotic-resistant bacteria

Recently, severe infection caused by escalating multidrug-resistant bacterial threats is considered as one of the most serious public health concerns^35^. For instance, notorious multidrug-resistant strains, including Gram-positive bacteria methicillin-resistant *S*. *aureus*, vancomycin-resistant *E*. *faecium*, and Gram-negative bacteria multidrug-resistant *P*. *aeruginosa*, have evolved as the sole reason for hospital- and community-acquired infections^36^. This facilitates a need for new antibiotics with great potency toward drug-resistant bacteria. Therefore, to probe the potency of our synthesized small molecules, **DL-5**, **DL-6**, **DG-5**, and **DG-6**, against drug resistance in bacteria, we screened them for their antibacterial activity against three MRSA strains (CCARM 3089, CCARM 3090, and CCARM 3095), a VREF strain (ATCC 51559), and two MDRPA strains (CCARM 2095 and CCARM 2109). Interestingly, all the compounds revealed almost considerable activities against all the strains compare to the reference compound, melittin (Table 2). Further, **DL-5** and **DG-5** displayed excellent efficacy against most of the bacterial strains either by showing similar or better activity with respect to the reference, and both followed similar activity trends.

**Table 2:** Antimicrobial activities of **DG-5**, **DG-6**, **DL-5**, **DL-6** and melittin against antibiotic-resistant bacterial strains.

Microorganisms	MIC (µM)				
	DG-5	DG-6	DL-5	DL-6	melittin
MRSA					
CCARM 3089	2	2	2	4	2
CCARM 3090	2	2	2	4	4
CCARM 3095	2	2	2	4	2
MDRPA					
CCARM 2095	4	8	4	8	2
CCARM 2109	4	8	4	8	4
VREF					
ATCC 51559	4	2	2	4	2

In particular, even though **DL-5** and **DG-5** showed a small reduction in efficiency against CCARM 2095 strain compared to that of reference, they found to be as potent as melittin against CCARM 2109. This enhanced activity against Gram-negative MDRPA strains may be likely due to the interaction between positive charges in **DL-5** and **DG-5** and the negative charge of the bacterial membrane to kill the pathogen. Moreover, **DG-6** also displayed similar or better activity against the majority of the strains except for MDRPA strains. Despite that, **DL-6** showed a slight decreased in activity against all the strains, and in the case of CCARM 3090, it showed an activity as potent as reference. Collectively, these results demonstrate that symmetrical hydrophobic groups of **DL-5**, **DL-6**, **DG-5**, and **DG-6** balance the charge produced either by two guanidyl or two amino groups. These results suggest that all our tested compounds could be used as a representative model for the design of a new class of antibacterial agents against drug-resistant bacteria.

### Salt resistance

To investigate the effects of salts on antimicrobial activity, these four compounds were tested for antibacterial activity against *E*. *coli* in the presence of different salts at physiological concentrations, because the salt resistance remains a prominent limitation to transform antibacterial compounds into novel therapeutic drugs^37^. As shown in Table S1 (see Supplementary), even though there was a slight drop in the antibacterial activity of **DL-5**, **DL-6**, **DG-5**, and **DG-6** compared to that in the absence of the salts, the resistance in the presence of all salts was still intact. In particular, **DG-5** and **DG-6** exhibited marked activity in the presence of all salts, where **DG-5** activity was decreased by two-fold compared to that in the control and **DG-6** showed similar or two-fold reduced activity compared to control. Similarly, **DL-5** and **DL-6** also showed considerable activity. These results indicate that the salt interference with the interaction between **DL-5**, **DL-6**, **DG-5**, and **DG-6** and the bacterial membrane was not extensive.

### Mechanistic studies

#### Cytoplasmic membrane depolarization

**Figure 5:**
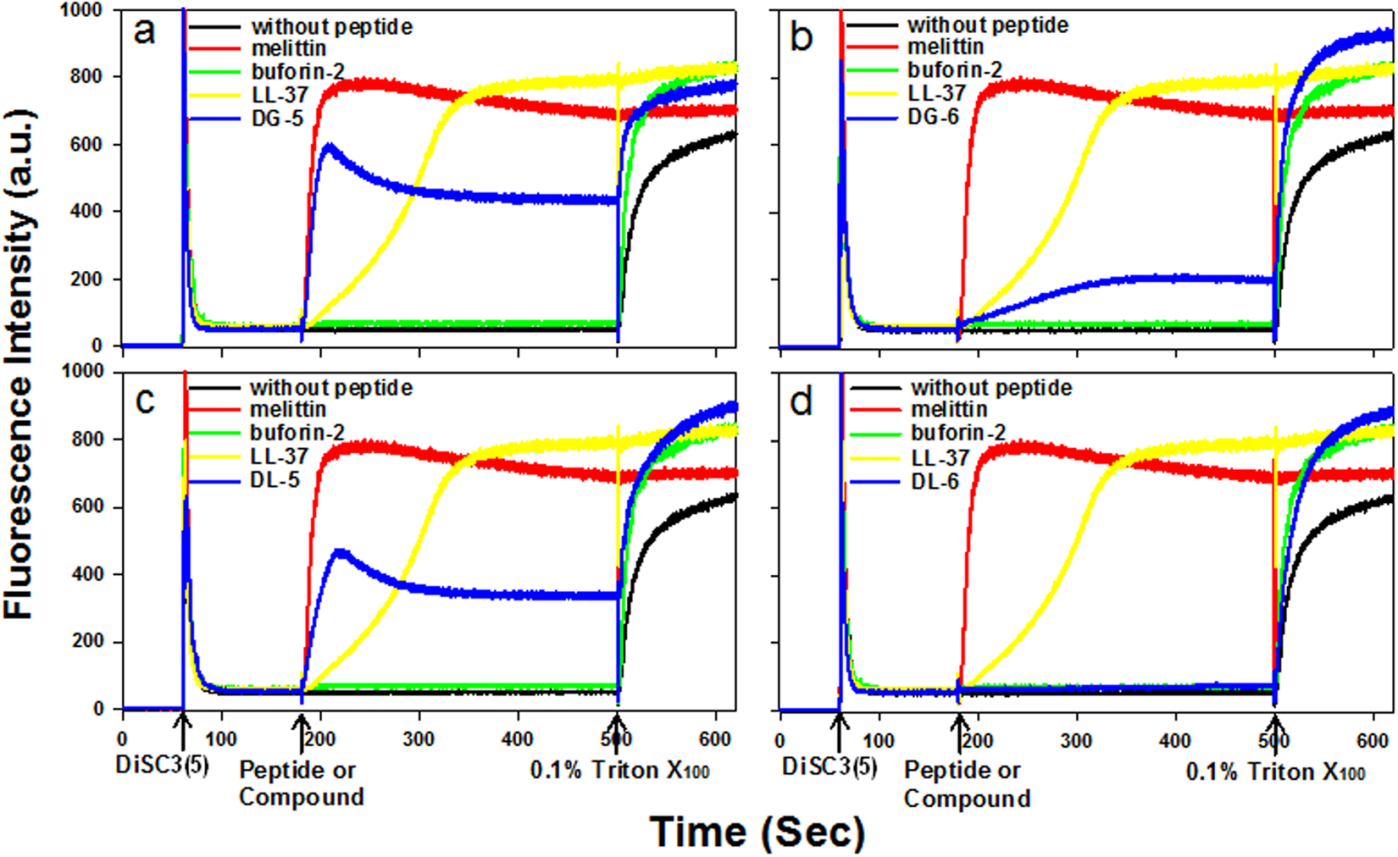
Depolarization effects of compounds (**DG-5** (**a**); **DG-6** (**b**); **DL-5** (**c**) and **DL-6** (**d**)) and peptides on *S*. *aureus* cytoplasmic membrane. The membrane potential-sensitive fluorescent dye 3,3′-dipropylthiadicarbocyanine Iodide (DiSC3(5)) was used for determining the *S*. *aureus* membrane depolarization by measuring fluorescence of the dye release (excitation λ = 622 nm, emission λ = 670 nm). Except for melittin (2 μM), the concentration of control antimicrobial peptides and antimicrobial compounds used is 2X MIC against *S*. *aureus* (KCTC 1621) (buforin-2 (16 μM); LL-37 (5 μM); **DG-5** (5 μM); **DG-6** (5 μM); **DL-5** (5 μM), **DL-6** (5 μM)). LL-37 and melittin were used as a positive control peptides and buforin-2 was used negative control peptide. The other two triplicate images are presented in Fig. S4 (SI).

To evaluate the mode of action of our synthesized compounds on the bacterial cytoplasmic membrane depolarization, we analyzed the depolarization ability of the compounds (**DL-5**, **DL-6**, **DG-5**, and **DG-6**) on *S*. *aureus* cytoplasmic membrane. We used 3,3′-Dipropylthiadicarbocyanine Iodide (DiSC3(5)), which is a membrane potential-dependent probe. During the permeabilization and disruption of the cytoplasmic membrane, there is an increase in fluorescence intensity due to the dispersion of probe into the medium. In this study, we used melittin and LL-37 as membrane-targeting positive controls. Buforin-2, known for its intracellular targeting mechanism, served as a negative control. As shown in Fig. 5(a–c), the inclusion of the synthesized small molecules **DL-5**, **DG-5**, and **DG-6** promoted a significant increase in fluorescence intensity similar to that exhibited by melittin and LL-37, indicating a rapid membrane depolarization. This result confirmed that **DL-5**, **DG-5**, and **DG-6** act as membrane targeting molecules, which caused a loss of membrane potential; however, membrane depolarization was not induced by buforin-2. Although **DL-5**, **DL-6**, and **DG-6** showed a similar pattern of mechanism to that exhibited by melittin, **DL-6** did not induce a significant increase in fluorescence at 2x MIC (Fig. 5d). In contrast, a slight increase in fluorescence was observed at 4x MIC (Fig. S3, ESI). This inference led to the speculation that **DL-6** may follow intracellular targeting mechanism similar to buforin-2.

#### Flow cytometry and Confocal laser-scanning microscopy

**Figure 6:**
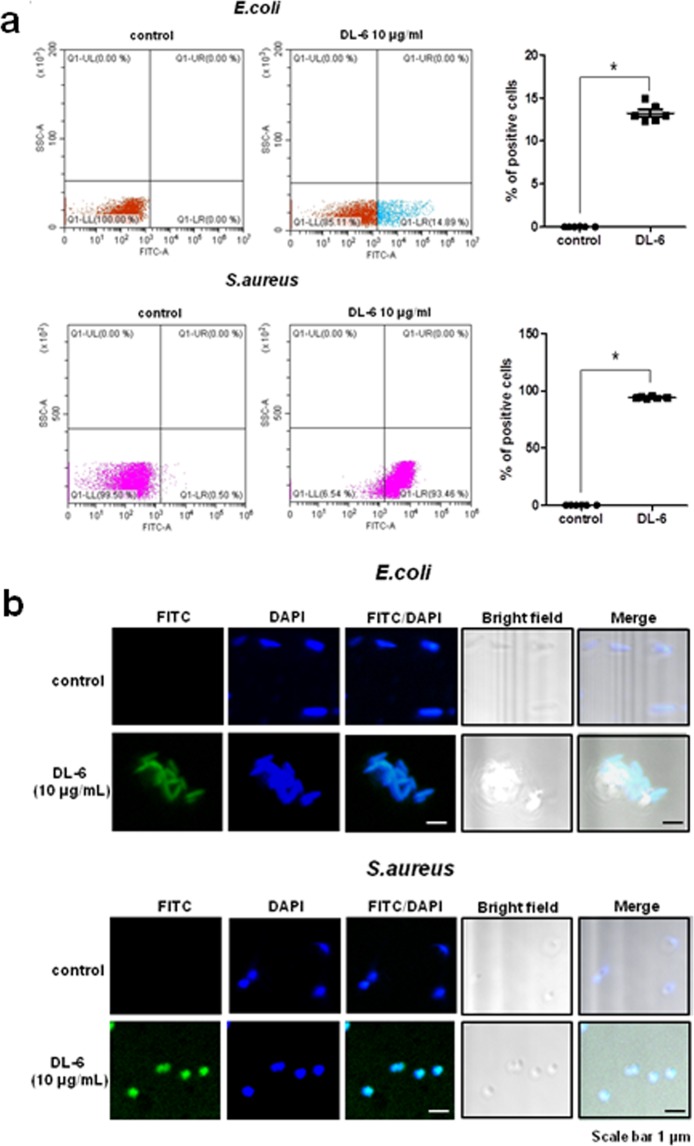
(**a**) Flow cytometry analysis of FITC-labeled **DL-6** uptake in bacteria; Quantification of positive bacterial cells with FITC labels. Each point represents +/−SEM of six repeated experiments *p* < 0.001. (**b**) Confocal fluorescence microscopy images of localization of FITC-labeled **DL-6** in *E*. *coli* and *S*. *aureus*.

To verify that **DL-6** follows intracellular targeting mechanism, FITC-labeled **DL-6** uptake in both Gram-negative and Gram-positive bacteria using *E*. *coli* and *S*. *aureus* respectively was confirmed by flow cytometry analysis. As shown in Fig. 6a, compared to the control, FITC-labeled **DL-6** showed a substantial shift in the fluorescence intensity towards the lower right-hand side region displaying 14.89% and 93.46% in *E*. *coli* and *S*. *aureus*, respectively, which can be attributed to the differences in the cell membrane of bacteria. To further confirm the hypothesis, we performed FITC-labeled **DL-6** (Fig. S1) uptake in *E*. *coli* and *S*. *aureus* using confocal laser-scanning microscopy (Fig. 6b). As observed in the merged portion (FITC/DAPI) of the images in both bacteria, there is a substantial increase in the fluorescence. This result indicated that FITC-labeled **DL-6** translocates inside the bacterial cell by accumulating at the cytoplasm of the cell, which suggest that **DL-6** exhibits intracellular targeting mechanism via cell penetration. However, still, we observed a few left out **FITC-DL-6** in *E*. *coli* because of the low permeability barrier of inner and outer membranes of bacteria (Fig. S5). This evidence further confirms that **DL-6** can translocate inside of both *E*. *coli* and *S*. *aureus* bacteria.

### Protease stability

The complexity rendered by the proteolytic instability of peptides in the presence of enzymes remains one of the major challenges in the advancement of peptide-based antibacterial for therapy, including cationic peptide antibiotics. It is known that lysine and arginine are highly prone to trypsin-mediated hydrolysis. Because the synthesized antibacterials (**DL-5**, **DL-6**, **DG-5**, **DG-6**) emerge as mimics of lysine and arginine, the proteolytic stability of these small molecules was investigated by tryptic degradation (see Supplementary Fig. S6). Incubation of these compounds with trypsin for 24 hours did not alter their antibacterial activity against *E*. *coli*. However, antibacterial activity of the reference melittin was lost entirely because of the proteolytic degradation by trypsin.

### Atopic dermatitis (AD)

AD can be classified as a complex and chronic inflammatory skin disease, which is characterized by a type 2 helper T cell (Th2) immune response and sensitization of immunoglobulin (Ig)E^38^. The pathogenesis of AD involves various factors including skin barrier dysfunction, susceptibility genes, and immunologic and environmental factors^39^. The most common environmental allergens for humans is house dust mites, including *Dermatophagoides pteronyssinus* and *Dermatophagoides fari*na (DFE)^40^. In general, the imbalance between Th1/Th2 cells remains the sole reason for the induction of AD, where Th2 and Th1 cells are responsible for promoting the acute and chronic phases of AD, respectively^41,42^.

**Figure 7:**
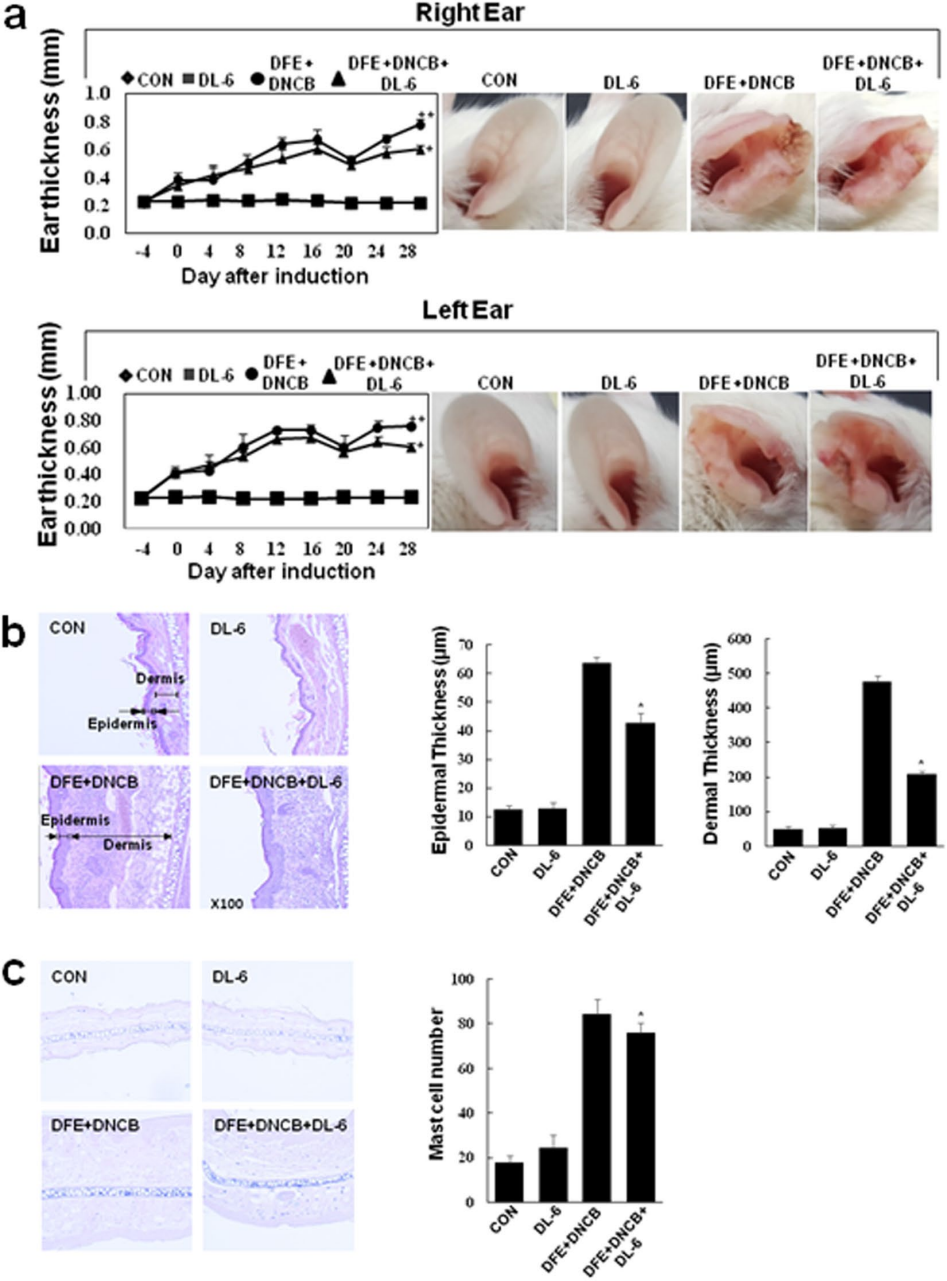
Histopathological analysis of **DL-6** on the ear thickness and mast cell infiltration. (**a**) The ear thickness measurement chart and day 28 mice photographs; (**b**) Measurement of the thickness of epidermis and dermis using hematoxylin and eosin-stained microphotographs; (**c**) Measurement of mast cells infiltration using toluidine blue staining. The data denote mean ± SD of six determinations of each group when n = 8. Asterisks indicate the significant difference from the AD value at *p* < 0.05. Original magnification was 200×. CON denotes the control. The pictures are representative images of each group (n = 8).

We found that most of our synthesized compounds possessed antibacterial activity along with considerable anti-inflammatory effects in LPS-induced inflammation. Hence, considering the proficient anti-inflammatory effects of our compounds, we anticipated that they could be effective in treating AD. Therefore, initially, we screened all the compounds for their ability to suppress the TNF*-α* expression (see Supplementary Fig. S7) in HaCaT cells using conventional RT-PCR. The results inferred that **DL**-**6** effectively suppressed TNF*-α* expression, even at 15 μM. Further, **DL-6** was evaluated for its cytotoxicity at different concentrations in HaCaT cells. The results revealed that **DL-6** did not show considerable toxicity at an elevated concentration (50 μM); more than 75% cell viability was observed (see Supplementary Fig. S8). Hence, the preliminary prerequisite for treating AD was successfully satisfied by **DL-6** in terms of both cytotoxicity and TNF-*α* suppressing effect. Therefore, **DL-6** was used for further studies on AD-like skin lesion in a BALB/c mouse model that was developed by alternate painting with *dermatophagoides farinae* extract (DFE) and 2,4-dinitrochlorobenzene (DNCB) for four weeks on both the earlobes of mouse (Fig. 7a). The repetitive application of DFE and DNCB induced swelling with a significant increase in the thickness of the ears.

However, treatment of **DL-6** with DFE and DNCB daily for 28 days exhibited remarkable effect on AD, which was evidenced by the suppression of the inflammation-induced thickness of the earlobes (Fig. 7a ear photographs). Microscopic images of DFE and DNCB-induced epidermal and dermal thickness were analyzed after treating with **DL-6**. As shown in Fig. 7b, treatment with **DL-6** significantly reduced the thickness of both epidermis and dermis. In particular, epidermal thickness was reduced by approximately 30% and dermal thickness by more than 50%, which proves the efficiency of **DL-6** over AD-lesion. The infiltration of mast cells in the ear tissue was also investigated by the toluidine blue staining of skin sections. Interestingly, as shown in Fig. 7c, **DL-6** decreased the infiltration of mast cells, which further proves the efficacy of **DL-6** against AD-like lesions.

**Figure 8:**
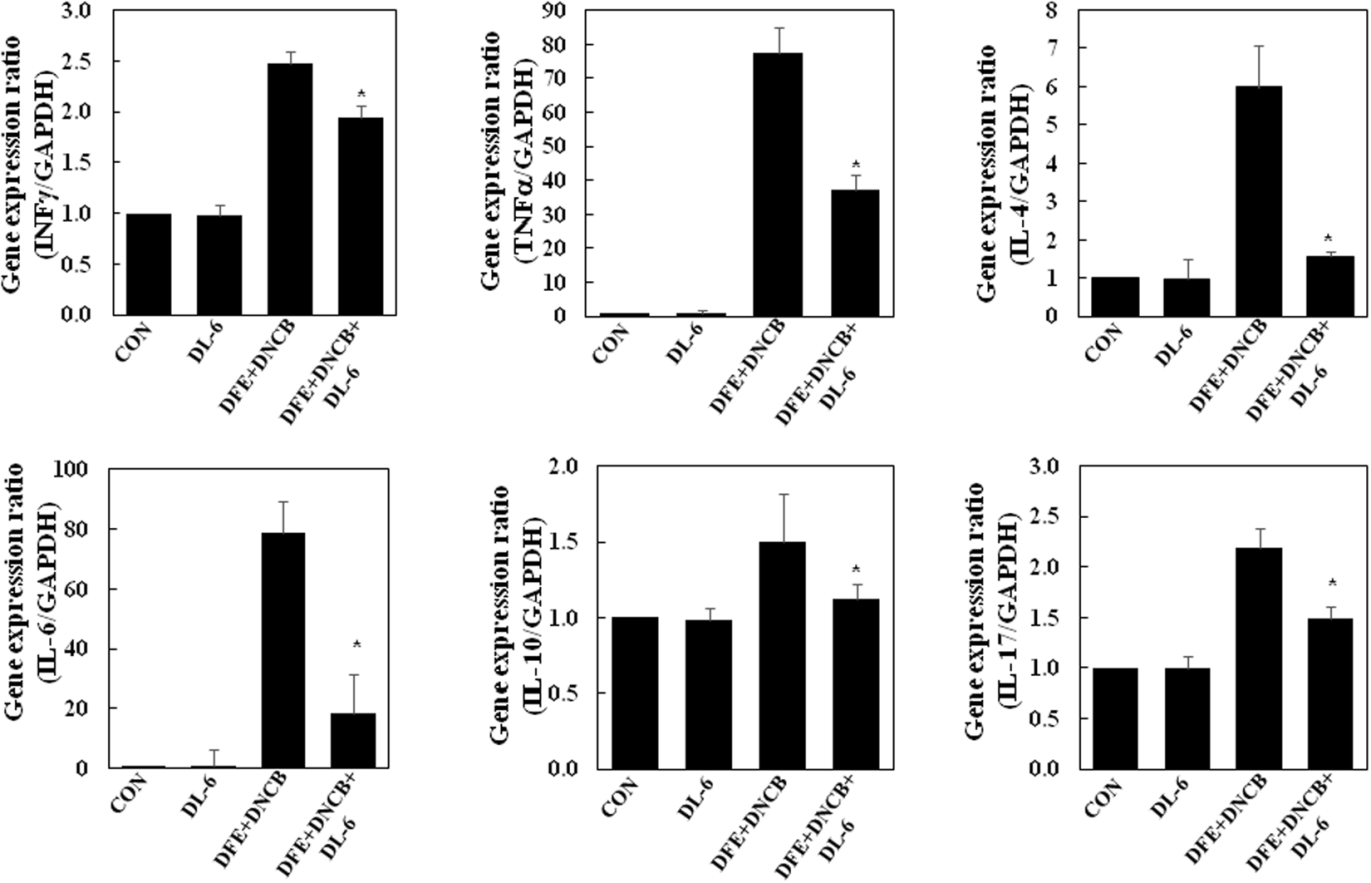
Anti-atopic efficacies of **DL-6** on the expression of various pathogenic factors in mice ear. After day 28, mice ears were excised, and total RNA was isolated, and performed the quantitative real-time polymerase chain reaction. The data indicate mean ± SD of triplicate experiments of each group, where n = 8. CON represents the control and asterisks indicate the significant differences from the AD value at *p* < 0.05.

It is reported that the Th2 cytokine, IL-4 is not only overexpressed in the acute phase of AD lesions but also, in many cases, IL-10 expressions also is predominant in AD lesions^43^. Also, there are reports pertaining to the increased levels of IL-17 in cutaneous lesions of AD patients in the acute lesions^44^. However, in the chronic phase of AD, Th1 producing IFN-*γ* and keratinocytes inducing proinflammatory cytokines TNF-*α* and IL-6 were abundant. We investigated the inhibitory effect of **DL-6** on inflammatory cytokine expressions in earlobes of mice with AD-like skin lesions using real-time PCR. As shown in Fig. 8, repeated oral application of DFE and DNCB increased the expressions of IFN-*γ*, TNF-*α*, IL-6, IL-4, IL-10, and IL-17. The treatment of **DL-6** significantly reduced the upregulated expression of cytokines in AD-like skin, irrespective of all Th1, Th2, and Th17.

It is pertinent to note that TNF-*α* expression was reduced by approximately 50%. Surprisingly, **DL-6** inhibited the expression of IL-4 and IL-6 by more than 75%, while that of IL-10 and IL-17 by approximately 25%. These results collectively infer the potency of **DL-6** in both acute and chronic inflammation in AD-lesions, which effectively suppressed cytokine expressions. As bacterial colonization is involved in the skin infection of AD patients, **DL-6** provides an opportunity for the development of AD inhibitors with potent antibacterial properties.

### *In vivo* anti-inflammatory activity of DG-5

**Figure 9:**
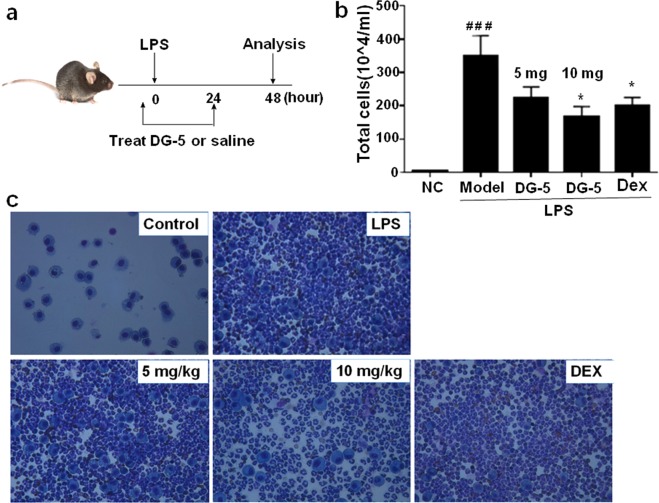
Anti-inflammatory effects of **DG-5** in LPS-induced mice BALF. (**a**) Timeline of LPS and **DG-5** treatment; (**b**) Total cells in mice BALF were isolated and quantified using Diff-Quick-stained reagent. ^#^*p* < 0.05, ^##^*p* < 0.01, and ^###^*p* < 0.001, compared with normal control (NC); *p < 0.05, **p < 0.01, and ***p < 0.001, compared with model group (two-way ANOVA); (**c**) Microscopic images of **DG-5** (5 mg and 10 mg doses) and dexamethasone (Dex) treatments in LPS induced lung inflammation cells.

Inspired by the inhibitory potency of **DG-5** over *in vitro* LPS-induced mRNA expressions of iNOS and TNF-*α*, we tested its efficacy in LPS-induced ALI mouse model to evaluate its therapeutic potential *in vivo*. To examine the effect of **DG-5**, we prepared LPS-induced ALI mouse models and compared the inflammatory efficiency by counting the inflammatory cells in bronchoalveolar lavage fluid (BALF) as shown in Fig. 9a. In the LPS-induced ALI mouse model, the numbers of total and differential inflammatory cells in BALF were remarkably high compared to those in the normal controls (NC). Oral administration of **DG-5** in two doses (5 mg and 10 mg) resulted in the appreciable suppression of inflammation in LPS-induced ALI mouse model based on the reduction in BALF cell counts (Fig. 9b).

Although oral administration of 5 mg dose showed reduced effects in suppression of inflammation, interestingly, treatment with 10 mg dose of **DG-5** displayed significant effects in suppression of inflammation as Dex. This observation was further ascertained by the microscopic images of **DG-5**-treated LPS-induced lung inflammation cells (Fig. 9c). Compared to the 5 mg dose of **DG-5**, 10 mg of **DG-5** displayed superior effect in the eradication of lung inflammation cells. These results strongly support that **DG-5** is capable of suppressing acute lung inflammation in addition to showing strong antibacterial activity.

## Conclusion

Though triazine derived antibacterials are reported^24,25,45^, the present work advances in terms of highly symmetrical design, enhanced antibacterial and anti-inflammatory effects, and enhanced potency against drug-resistant pathogens. Our findings from this work demonstrate that substituted triazines are suitable for designing potent antibacterial agents due to their symmetric structures holding amphipathicity as in CPAs and easy access to the synthesis of various derivatives, which led to the identification of four lead compounds **DG-5**, **DG-6**, **DL-5**, and **DL-6**. These compounds have not only antibacterial and anti-inflammatory effects but also show significant potency against drug-resistant bacteria. Mechanistic evidence revealed that **DG-5**, **DG-6**, and **DL-5** were found to be membrane targeting and in contrast, **DL-6** followed an intracellular targeting mechanism. Along with the potent *in vitro* anti-inflammatory effects, **DG-5** and **DL-6** also showed their remarkable potency in *in vivo* ALI mouse model and atopic BALB/c mouse model, respectively. Thus, these compounds have the potential for future use as a therapeutic model. Together with their facile synthesis, these compounds could be a new class of drug-resistant, protease stable small molecular antibacterial agents with potent anti-inflammatory and anti-atopic dermatitis. In addition, further studies on atopic dermatitis are under progress using other *in vivo* models.

## Materials and Methods

### Chemistry

As described in our previous article^46^, all reagents and starting materials were purchased from commercial chemical suppliers (Sigma-Aldrich, TCI and Across Organics) and used as received. All the anhydrous organic solvents of purity greater than 99.9% were purchased from Aldrich and used directly. Thin layer chromatography (TLC) was performed on Merck aluminum sheets with silica gel 60 F254 and was visualized by ultraviolet light and staining with KMnO_4_ and ninhydrin. For the purification of compounds, column chromatography was performed on Merck silica gel 60 (70–230 mesh or 230–400 mesh). NMR spectra including, ^1^H and ^13^C NMR spectra were recorded on a Bruker DRX-400 and DRX-500 spectrometer. Chemical shifts (δ) are reported in parts per million (ppm) measured and coupling constants (*J*) are given in hertz (Hz). MALDI-TOF mass were recorded using Shimadzu mass spectrometer. Reverse-phase HPLC analysis (RP-HPLC) was carried out at 230 nm on an Agilent HPLC system equipped with a C18 analytical column (250 × 22 mm, 10 microns). Two different linear gradients of 0.05% aq. trifluoroacetic acid (TFA) (eluent A) and 0.05% TFA in CH_3_CN (eluent B) were used with a flow rate of 5.0 mL/min at 25 °C.

### Bacterial strains and Growth Conditions

As described in our previous articles^26^, two Gram-positive bacterial strains (*Staphylococcus aureus* [KCTC 1621] and *Staphylococcus epidermidis* [KCTC 1917]), and two Gram-negative bacterial strains (*Pseudomonas aeruginosa* [KCTC 1637] and *Escherichia coli* [KCTC 1682]) were received from the Korean Collection for Type Cultures (KCTC) of the Korea Research Institute of Bioscience and Biotechnology (KRIBB). Culture Collection of Antibiotic-Resistant Microbes (CCARM) (Seoul Women’s University in Korea) supplied the methicillin-resistant *Staphylococcus aureus* strains (MRSA; CCARM 3089, CCARM 3090, and CCARM 3095) and multidrug-resistant *Pseudomonas aeruginosa* strains (MDRPA; CCARM 2095, and CCARM 2109) were obtained from the. American Type Culture Collection (Manassas, VA, USA) supplied the vancomycin-resistant *Enterococcus faecium* (VREF; ATCC 51559). All bacterial strains were made as glycerol stocks and stored at −80°, cultured on Luria Broth agar plates, and stored at 4 °C. Before the experiments, the single bacterial colony was chosen from the agar plates and cultured in LB media for overnight at 37 °C. Then, subcultures were prepared and incubated at 37 °C for 3 h or until the OD600 reached 0.5, which corresponds to 2 × 108 CFU/mL.

### Antimicrobial activity

As described in our previous articles^47^, MICs were determined by the standard broth microdilution method as previously described. Briefly, 96-well polystyrene plates were inoculated with two-fold serial dilutions of the small molecule compounds (starting at 128 μM) and a log-phase culture of the target bacterial strain (2 × 10^6^ CFU/mL), which were both diluted in 1% peptone. The plates were subsequently incubated for 18–24 h at 37 °C and 200 rpm in a shaking incubator. The MIC is defined as the lowest concentration that inhibits bacterial growth as determined visually or spectrophotometrically by OD_600_. In order to assess the effects of different salts on the antimicrobial activity of the synthesized small molecules, MICs were also determined in the presence of salts. *E*. *coli* (KCTC 1682) at a concentration of 2 × 10^6^ CFU/mL were treated with small molecule compounds in solutions containing physiological concentrations of various salts (150 mM NaCl, 4.5 mM KCl, 6 µM NH_4_Cl, 1 mM MgCl_2_, 2.5 mM CaCl_2_, 4 µM FeCl_3_), added to 1% peptone. With the exception of the added salt, MICs were determined using the assay described above.

### Hemolytic Activity

As similar to our previous articles^47^, sheep red blood cells (sRBCs) in PBS were washed repeatedly three times or until the supernatant was clear (centrifuged at 2000 rpm for 5 min). Two-fold serial dilutions of the synthesized antibacterial agents in phosphate buffered saline (PBS) (concentrations varying from 1 to 320 μM) were added to 96-well polystyrene plates incubated with sRBCs to a final concentration of 4% (v/v) for 1 h at 37 °C. Samples were centrifuged at the speed of 800 × g for 10 min, cell supernatants were transferred to clear 96-well plates, and their absorbance was measured at 405 nm. The OD value of PBS was considered to be 0% hemolysis, while 100% hemolysis was established using 0.1% Triton X-100.

### Cytotoxicity against Mammalian Cells

As described in our previous article^47^ MTT assays were performed to determine the cytotoxicity of the synthesized small molecule compounds against RAW 264.7 macrophage cells. The cells were seeded into 96-well plates at 2 × 10^4^ cells per well and incubated for 24 h at 37 °C in 5% CO_2_ to ensure full confluency. Cells were treated with serial dilutions of small molecule compounds in DMEM ranging from 0.313 to 20 μM (final) and further incubated for 48 h. Cells without the added small molecule antibacterial agents served as controls. After incubation, the cell media were replaced with 100 μL of fresh media containing MTT solution (5 mg/mL), and cells were incubated for another 4 h at 37 °C. Next, the media were replaced with 100 μL of DMSO and metabolic activities were detected spectrophotometrically by measuring the absorbance at 550 nm as previously described. Cell proliferation values were expressed as percentages and calculated as follows: (Abs_550_ nm of treated sample)/(Abs_550_ nm of control) × 100.

### Measurement of NO production from LPS-stimulated RAW264.7 cells

As described in our previous articles^26,47^, NO production from LPS-stimulated RAW264.7 cells was determined. Briefly, RAW264.7 cells were plated at a density of 5 × 10^5^ cells/mL in 96-well culture plates and stimulated with LPS (20 ng/mL) in the presence or absence of synthesized small molecule compounds for 24 h. Isolated supernatant fractions were mixed with an equal volume of Griess reagent (1% sulfanilamide, 0.1% naphthylethylenediamine dihydrochloride and 2% phosphoric acid) and incubated at room temperature for 10 min. Nitrite production was quantified by measuring absorbance at 540 nm, and concentrations were determined using a standard curve generated with NaNO_2_.

### Measurement of release of TNF-α from LPS-stimulated RAW264.7 cells

As described in our previous articles^26,47^, RAW264.7 cells were seeded in 96-well plates (5 × 10^4^ cells/well) and incubated overnight. The synthesized small molecule compounds were added and the cultures were incubated at 37 °C for 1 h. Subsequently, 20 ng/mL of LPS was added and the cells were incubated for another 6 h at 37 °C. Cell supernatants were collected and stored at −20 °C until needed for analysis of cytokine levels. Levels of TNF-*α* in the samples were measured by the commercial enzyme-linked immunosorbent assay kit (R&D Systems, Minnespolis, MN, USA) according to the manufacturer’s instructions.

### Reverse-transcription polymerase chain reaction (RT-PCR)

As described in our previous article^47^, RAW264.7 cells were plated at a concentration of 5 × 105 cells/well in six-well plates and incubated overnight. Each peptide was added to the wells. The final concentration of the small molecule compounds was 10 μM (for NO) or 20 μM (for TNF-*α*). Cells to be used for quantification of TNF-*α* and iNOS were treated for 3 h and 6 h, respectively, with or without (negative control) 20 ng/mL LPS, in the presence or absence of peptide, in DMEM supplemented with 10% bovine serum. The cells were detached from the wells and washed once with PBS. Total RNA was extracted by Trizol Reagent (Ambion, USA). Then, 1 μg of total RNA was converted to cDNA using a TakaRa Primescript^TM^ Reverse Transcriptase (TakaRa, Seoul, Korea). The primers used were purchased from Bioneer (Seoul, Korea). The cDNA products were amplified by AccuPower® PCR PreMix (Bioneer, Daejeon, Korea). For iNOS (forward, 5′-CTGCAGCACTTGGATCAGGAACCTG-3′; reverse, 5′-GGGAGTAGCCTGTGTGCACCTGGAA-3′), TNF-α (forward, 5′-CCTGTAGCCCACGTCGTAGC-3′; reverse, 5′-TTGACCTCAGCGCTGAGTTG-3′), or GAPDH (forward, 5′-GACATCAAGAAGGTGGTGAA-3′; reverse, 5′-TGTCATACCAGGAAATGAGC-3′). The amplification procedure consisted of an initial denaturation step of 5 min at 94 °C, followed by 30 cycles of denaturation at 94 °C for 1 min, annealing at 55 °C for 1.5 min, and extension at 72 °C for 1 min, and afterwards by a final extension step of 5 min at 72 °C.

### Protease stability by radial diffusion assay

As described in our previous article^26^, *E*. *coli* (KCTC 1682) was grown overnight for 18 h at 37 °C in 10 mL of LB broth, and then 10 mL of this culture was inoculated into 10 mL of fresh LB and incubated for an additional 3 h at 37 °C to obtain mid-logarithmic-phase organisms. A bacterial suspension (2 × 10^6^ CFU/mL in LB) was mixed with 0.7% agarose. This mixture was poured into a 10-cm petri dish, and it dispersed rapidly. Five microliters of an aqueous peptide stock solution (10 mg/mL) were added to 25 mL of trypsin solution in PBS (0.2 mg/mL) and incubated at 37 °C for 4 h. The reaction was stopped by freezing with liquid nitrogen, after which 10 mL aliquots were placed in each circle paper (<6 mm in diameter), put on the agarose plates, and then incubated at 37 °C overnight. The diameters of the bacterial clearance zones surrounding the circle paper were measured for the quantitation of inhibitory activities.

### Cytoplasmic membrane depolarization assay

As described in our previous articles^26,47^, the effect of synthesized small molecule antibacterial agents on cytoplasmic membrane depolarization was determined using the membrane potential-sensitive dye, DiSC3(5), as previously described^48,49^. Briefly, *S*. *aureus* (KCTC 1621) grown at 37 °C with agitation to the mid-log phase (OD_600_ = 0.4) was harvested by centrifugation. Cells were washed twice with washing buffer (20 mM glucose and 5 mM HEPES, pH 7.4) and resuspended to an OD_600_ of 0.05 in the same buffer. The cell suspension was incubated with 20 nM DiSC3(5) until a stable fluorescence value was achieved, implying the full incorporation of the dye into the bacterial membrane. Membrane depolarization was monitored based on the changes in the intensity of the fluorescence emitted from the DiSC3(5) (excitation λ = 622 nm, emission λ = 670 nm) after peptide addition. The membrane potential was fully abolished by adding 0.1% Triton X_100_.

### Statistical analysis

As described in our previous article^47^, all results are presented as averages of values derived from three independent experiments, with triplicates used in each experiment. Error bars represent the mean ± standard deviation of the mean. The statistical significance of differences between samples and respective controls (no added antimicrobial agent) were determined by one-way analysis of variance (ANOVA) with Bonferroni’s post-test method using Sigma plot v12.0 (Systat Software Inc., San Jose, CA, USA). Differences with *P* < 0.001 were considered statistically significant.

### Confocal fluorescence microscopy

Bacteria was collected to the extent of 10^7^ CFU, then it was resuspended in phosphate buffer (PBS) containing FITC-labeled **DL-6** at 10 μg/mL and incubated for 1 hour at 37 °C. Subsequently, the cells were washed, fixed, DAPI stained and immobilized on poly-L-lysine coated glass slides. Before mounting, ProLong Gold antifade reagent (Invitrogen) was added to the slides. Finally, localization of FITC-labeled **DL-6** was observed using LSM710 (Carl Zeiss, Germany) confocal microscopy equipped with a 100X oil immersion lens.

### Flow cytometry analysis

FITC-labeled **DL-6** uptake in bacteria was confirmed by flow cytometry analysis. Bacteria was resuspended in PBS containing FITC-labeled **DL-6** at a 10 μg/mL and incubated for 1 hour at 37 °C. Subsequently, cells were washed and analyzed using a flow cytometer (CytoFlex, Beckman Coulter, Miami, USA). The laser excitation wavelength remained at 488 nm and emission band-pass wavelengths were at 525/40 nm. The results are described as the median of the distribution of FITC intensity obtained by analyzing 10,000 bacteria.

### *In vivo* study

#### Animals

As reported our previously reported article^50^, six-week-old male C57BL/6J mice (20–22 g) were obtained from the KRIBB Laboratory Animal Resource Center (Ochang, Korea) and housed in environmentally controlled pathogen-free conditions throughout the experiments. All experimental procedures were approved by the Institutional Animal Care and Use Committee of the Korea Research Institute of Bioscience and Biotechnology and performed in compliance with the National Institute of Health Guidelines for the care and use of laboratory animals and the Korean National Animal Welfare Law.

#### Mouse model of LPS-induced ALI (Acute Lung Injury)

Mice (n = 3) were grouped based on the experimental design. (i) control group (NC), which received saline; (ii) LPS-induced ALI group (model), which received LPS; (iii) LPS-received low-dose **DG-5** group, which received 5 mg/kg **DG-5** and LPS; (iv) LPS-received high-dose **DG-5** group, which received 10 mg/kg **DG-5** and LPS; (v) LPS-received dexamethasone group (Dex), which received 1 mg/kg dexamethasone and LPS. Experimental mice were subjected to an intranasal instillation of 10 μg of LPS (Sigma-Aldrich, St. Louis, MO) or PBS. **DG-5** and dexamethasone were administered to mice by oral gavage (1 h before and 24 h after LPS treatment). The dose of **DG-5** chosen in the present study was based on the results from *in vitro* assays.

#### Bronchoalveolar lavage fluid (BALF)

BALF collection was performed as previously described^50^. The trachea was cannulated, and the BALF was obtained by injecting ice-cold PBS (0.5 mL) and withdrawing as much fluid as possible using a tracheal cannula with a 20G blunt needle. The procedure was repeated once for each mouse, and the fluid was transferred to a test tube on ice. For total and differential cell counting, BALF was centrifuged on cytospin slides for 5 min at 1500 rpm. The slides were dried, and the inflammatory cells were fixed and stained using Diff-Quik® staining reagent (IMEB Inc, Deerfield, IL) according to the manufacturer’s instructions.

### Atopic dermatitis

#### Cell culture

As described in our previous article^51^, in this AD study, Human keratinocytes HaCaT cells were broadly used. The HaCaT cells were cultured in a 5% CO_2_ atmosphere at 37 °C in Dulbecco’s modified Eagle’s medium (DMEM) supplemented with 10% fetal bovine serum (FBS) and antibiotics (10 μg/100 mL penicillin/streptomycin). To perform PCR and western blotting, HaCaT cells were pre-incubated with 10 ng/mL of both TNF-*α* and IFN-*γ* for the stimulation. After 6 h of stimulation, cells were harvested.

#### Cytotoxicity Assay

As described in^52^, cell viability was determined by 3-(4,5-dimethylthiazol-2yl)-2,5-diphenyltetrazolium bromide (MTT) assay in 96-well plates. Cells were incubated with various concentrations of **DL-6** for 48 h followed by MTT for 4 h, and then 100 μL isopropanol (in 0.04 N-hydrochloric acid) was added to dissolve the formazan crystals. The absorbance was read at 570 nm using a spectrophotometer (Tecan, Switzerland). Cell viability was calculated as the relative absorbance compared to DMSO vehicle control absorbance^52^.

#### Animals

As described in our previous article^53^, eight-week-old female BALB/c mice were purchased from Samtako and housed under specific pathogen-free conditions. All experiments were approved by the Institutional Animal Care and Use Committee of Konkuk University (KU17089).

#### Induction of AD lesions in the ear

As previously described in our article^53^, AD was induced in the mice by repeated local exposure of *Dermatophagoides farinae* extract (DFE; house dust mite extract) and 2,4-dinitrochlorobenzene (DNCB) to the ears. For the induction of AD, the mice were divided into four groups (control, **DL-6**-only, AD-only, and AD + **DL-6**), and the surfaces of both earlobes were stripped five times with surgical tape (Nichiban, Tokyo, Japan). After stripping, 20 µL of 1% DNCB was painted onto each ear, followed by 20 µL of DFE (10 mg/mL) 4 days later. Animals received **DL-6** (20 μL of 20 mg/mL, topical application) throughout the 4 weeks of AD induction. The ear thickness was measured 24 h after DNCB and DFE application with a dial thickness gauge (Kori Seiki MFG, Co., Japan).

#### Histological observations

As described in our previous article^53^, excised ears were fixed in 4% paraformaldehyde for 16 h and embedded in paraffin. Thin (6 µm) sections were stained with hematoxylin and eosin (H&E). The thickness of the epidermis and dermis was measured under a microscope. For measurement of mast cell infiltration, skin sections were stained with toluidine blue and the number of mast cells was counted in five randomly chosen fields of view.

#### Real-time polymerase chain reaction (PCR)

The conditions for PCR were similar to those previously described^53^. The total RNA was isolated from cells using TRIzol according to the manufacturer’s protocol. The first-strand complementary DNA (cDNA) was synthesized using Superscript II reverse transcriptase (Invitrogen). Quantitative real-time PCR was carried out using a Thermal Cycler Dice TP850 (Takarabio Inc., Shiga, Japan) according to the manufacturer’s protocol. Total RNA was isolated from the ear tissues of each group. Procedure in Briefly, 2 μL of cDNA (100 ng), 1 µL of sense and antisense primer solution (0.4 µM), 12.5 µL of SYBR Premix Ex Taq (Takarabio Inc.), and 9.5 µL of dH_2_O were mixed to obtain a final 25 µL reaction mixture in each reaction tube. The primers used for PCR were: mouse tumor necrosis factor-alpha (*TNF-α*, 5′-AAGCCTGTAGCCCACGTCGTA-3′ and 5′-GGCACCACTAGTTGGTTGTCTTTG-3′; mouse *IFN-γ*, 5′-TCAAGTGGCATAGATGTGGAAGAA-3′ and 5′-TGGCTCTGCAGGATTTTCATG-3′; mouse *IL-4*, 5′-ACAGGAGAAGGGACGCCAT-3′ and 5′-GAAGCCGTACAGACGAGCTCA-3′; mouse *IL-6*, 5′-CCGGAGAGGAGACTTCACAG-3′ and 5′-GGAAATTGGGGTAGGAAGGA-3′; mouse *IL-10*, 5′-TCAGCTGTGTCTGGGCCACT-3′ and 5′-TTATGAGTAGGGACAGGAAG-3′; mouse *IL-17*, 5′-CCTACCAGACCAAGGTCAAC-3′ and 5′-AGGGGGTAATAAAGGGATTG-3′; mouse glyceraldehyde 3-phosphate dehydrogenase (*GAPDH)*, 5′-GCACAGTCAAGGCCGAGAAT-3′ and 5′-GCCTTCTCCATGGTGGTGAA-3′. The amplification conditions were 10 s at 95 °C, 40 cycles of 5 s at 95 °C and 30 s at 60 °C, 15 s at 95 °C, 30 s at 60 °C, and 15 s at 95 °C. The mRNA levels of the target genes, relative to *GAPDH*, were normalized using the following formula: relative mRNA expression = 2^−(ΔCt of target gene−ΔCt of *GAPDH*)^, where Ct is the threshold cycle value. In each sample, the expression of the analyzed gene was normalized to that of *GAPDH* and presented as the relative mRNA level.

## Supplementary information

Supplementary Information

## Data Availability

All data generated or analysed during this study are included in this article and its supplementary information files.
