# Supplementary material for: Cationic Amphipathic Triazines with Potent Anti-bacterial, Anti-inflammatory and Anti-atopic Dermatitis Properties
Source: Sci Rep. 2019 Feb 4;9:1292. doi: 10.1038/s41598-018-37785-z (PMC6361992; doi:10.1038/s41598-018-37785-z)
Supplement: Supplementary file 1 — Supplementary Information [file 41598_2018_37785_MOESM1_ESM.docx]

**Cationic Amphipathic Triazines with Potent Anti-bacterial, Anti-inflammatory and Anti-atopic Dermatitis Properties**

Pethaiah Gunasekaran^1,+^, Ganesan Rajasekaran^2,+^, Eun Hee Han^3^, Young-Ho Chung^3^, Young-Jin Choi^4^, Yu Jin Yang^1^, Ji Eun Lee^1,7^, Hak Nam Kim^1^, Kiram Lee^5^, Jin-Seok Kim^5^, Hyun-Jun Lee^5^, Eun-Ju Choi^6^, Eun-Kyung Kim*^,4^, Song Yub Shin*^,2^, Jeong Kyu Bang*^,1,7^

^1^Division of Magnetic Resonance, Korea Basic Science Institute (KBSI), Ochang, Chung Buk, 28119, Republic of Korea. E-mail: [bangjk@kbsi.re.kr](mailto:bangjk@kbsi.re.kr), Tel: +82-43-240-5023, Fax: +82-43-240-5059

^2^Department of Cellular and Molecular Medicine, Chosun University, Gwangju, 501-759, Republic of Korea. E-mail: syshin@chosun.ac.kr

^3^Drug & Disease Target Research Team, Korea Basic Science Institute (KBSI), Cheongju 28119, Republic of Korea

^4^Division of Food Bioscience, Konkuk University, Chungju 27478, Republic of Korea. E-mail: eunkyungkim@kku.ac.kr

^5^Natural Medicine Research Center, Korea Research Institute of Bioscience and Biotechnology, Ochang-eup, chungcheongbuk-do 28116, Republic of Korea

^6^Department of Physical Education, Daegu Catholic University, Gyeongsan 38430, Republic of Korea

^7^Department of Bio-analytical Science, University of Science & Technology, Daejeon, 34113, Republic of Korea.

^+^These authors contributed equally to this work

*Corresponding authors

Correspondence and requests for materials should be addressed to J.K.B. (email: bangjk@kbsi.re.kr), S.Y.S. (email: syshin@chosun.ac.kr) or E.K.K. (email: eunkyungkim@kku.ac.kr)

Table of Contents

| **No** | **Contents** | **Page** |
| --- | --- | --- |
| **1** | **Figures S1-S8** | **S3-S7** |
| **2** | **Table S1** | **S7** |
| **3** | **Full-length blots of *Figure 4c*** | **S8** |
| **4** | **Full-length blots of DG compounds in *Figure S7A*** | **S9** |
| **5** | **Full-length blots of DL compounds in *Figure S7B*** | **S10** |
| **6** | **Full-length blots of DL-6 in *Figure S8A*** | **S10** |
| **7** | **Experimental section-chemistry** | **S11-S23** |
| **8** | **1H &13C NMR spectra of all compounds** | **S24-S105** |


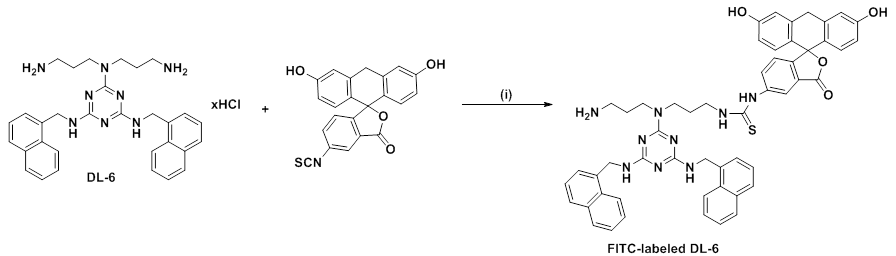


**Figure S1.** Synthesis of FITC (fluorescein isothiocyanate) labeled **DL-6**: Reagents and conditions**:** (i). diisopropylethylamine (DIEA), dichloromethane (DCM), 0-5 ^o^C, 3 h, 52 %

**Figure S2.** Inhibition of compounds (10 μM) on LPS-stimulated TNF-*α* release from RAW264.7 cells. Asterisks dictate the significant effects of the tested compounds compared to that of LPS treated cells. One-way ANOVA with Bonferroni's post-test (**p* < 0.001 for each agonist) used for analyzing data. The data are mean ± SEM of three experiments. The results remained similar when experiments were performed using different cells. Control indicates the cell only.


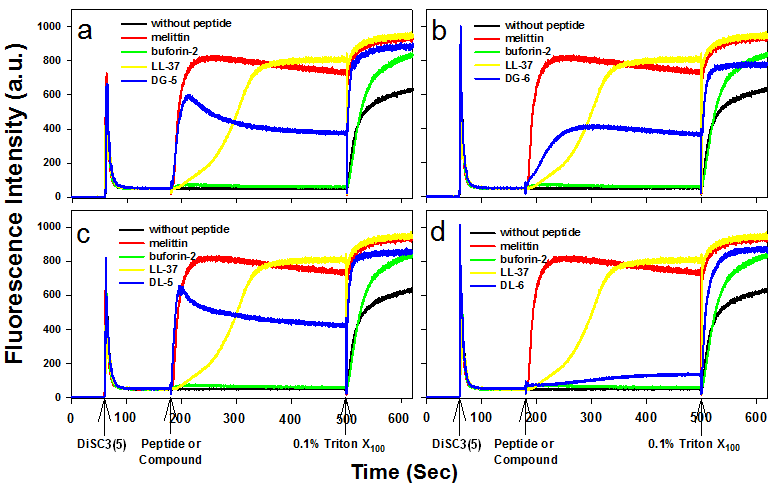


**Figure S3.** Depolarization effects of compounds (**DG-5** (a); **DG-6** (b); **DL-5** (c) and **DL-6** (d)) and peptides on *S. aureus* cytoplasmic membrane. The membrane potential-sensitive fluorescent dye 3,3'-dipropylthiadicarbocyanine Iodide (DiSC3(5)) was used for determining the *S. aureus* membrane depolarization by measuring fluorescence of the dye release (excitation λ = 622 nm, emission λ = 670 nm). Except for melittin (2 μM), the concentration of control antimicrobial peptides and antimicrobial compounds used is 4X MIC against *S. aureus* (KCTC 1621) (buforin-2 (32 μM); LL-37 (10 μM); **DG-5** (10 μM); **DG-6** (10 μM); **DL-5** (10 μM); **DL-6** (10 μM)). LL-37 and melittin were used as a positive control peptides and buforin-2 was used negative control peptide.


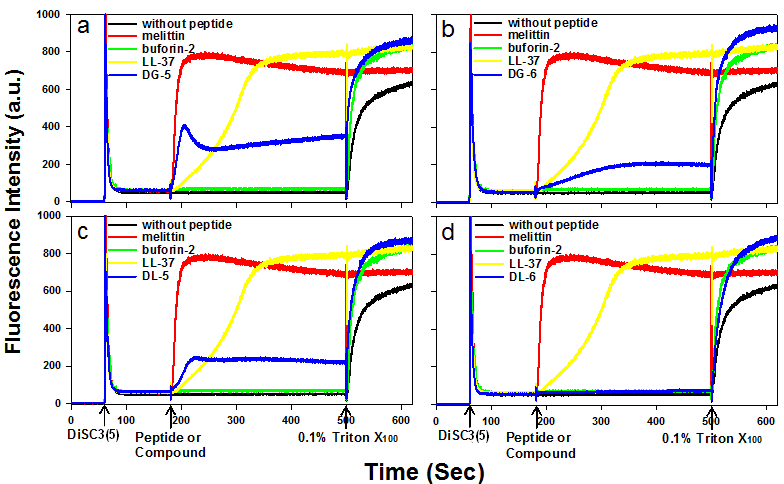

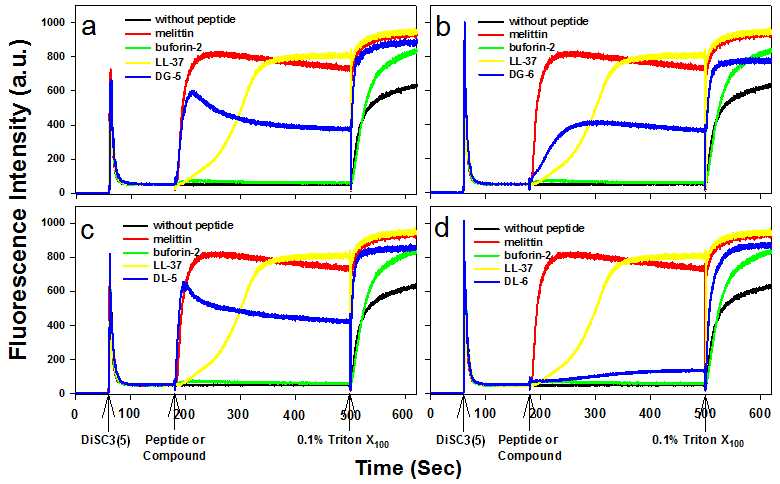


**Figure S4.** The other two images of Figure 5.


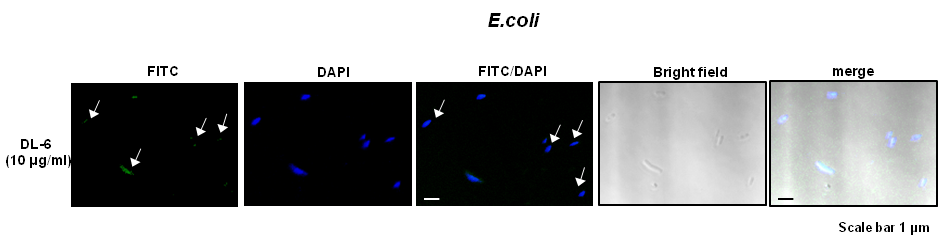


**Figure S5:** Confocal fluorescence microscopy image of localization of FITC-labeled **DL-6** in *E. coli*. White arrows indicate FITC negative cells.


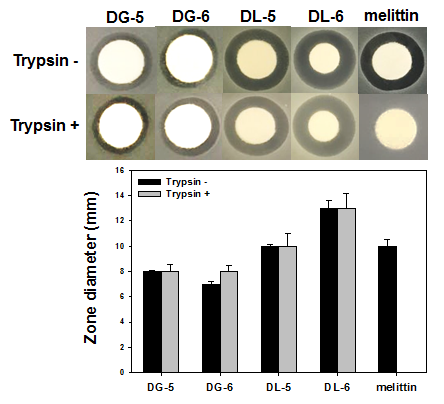


**Figure S6.** Trypsin mediated inhibition of antimicrobial activity of compounds in *E. coli*, assessed by a radial diffusion assay.


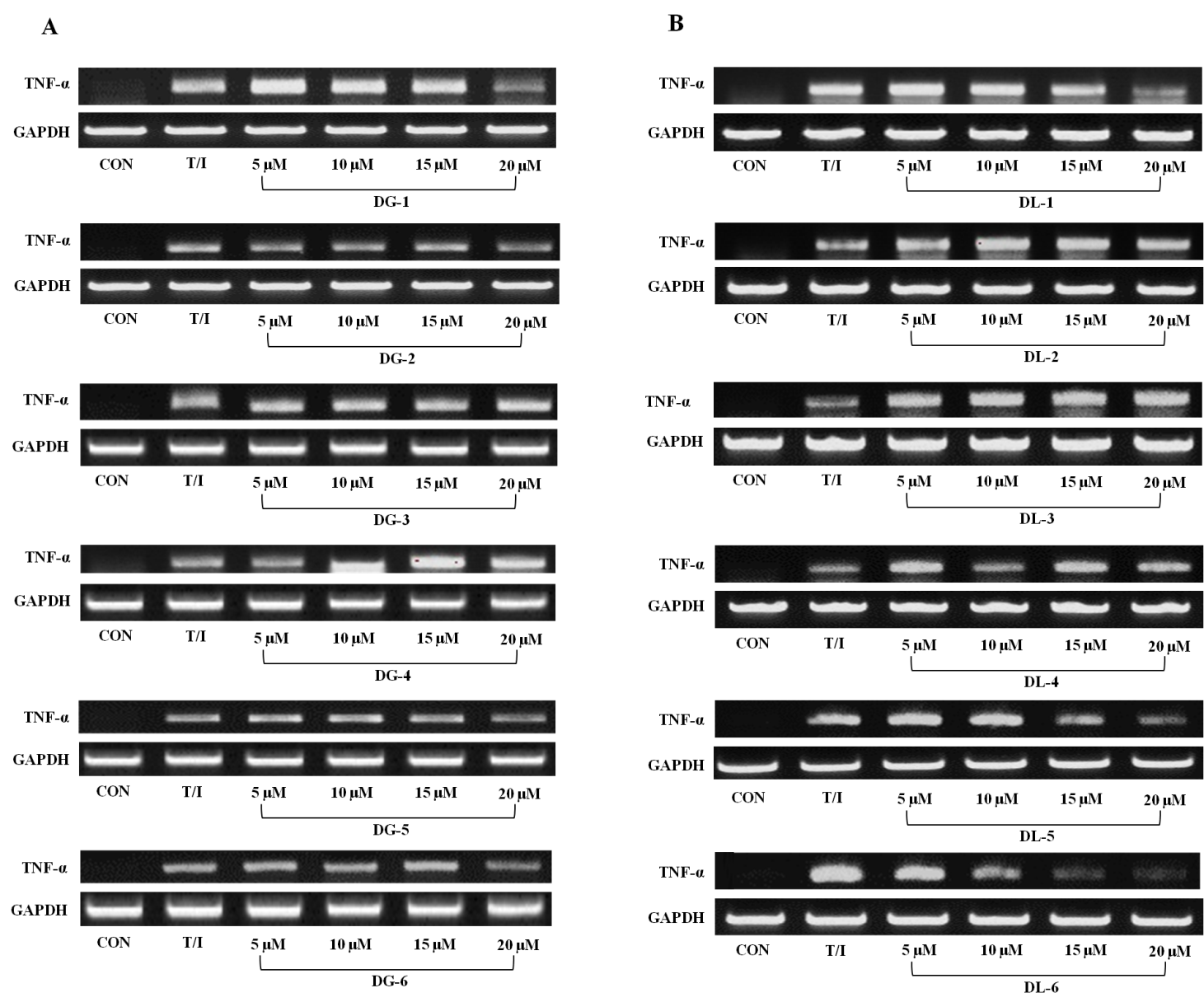


**Figure S7.**  DG & DL screening on HaCaT cells. Gene expression levels of TNF-α was measured by conventional RT-PCR (***full-length blots are given in page S9 and S10***)

.

.


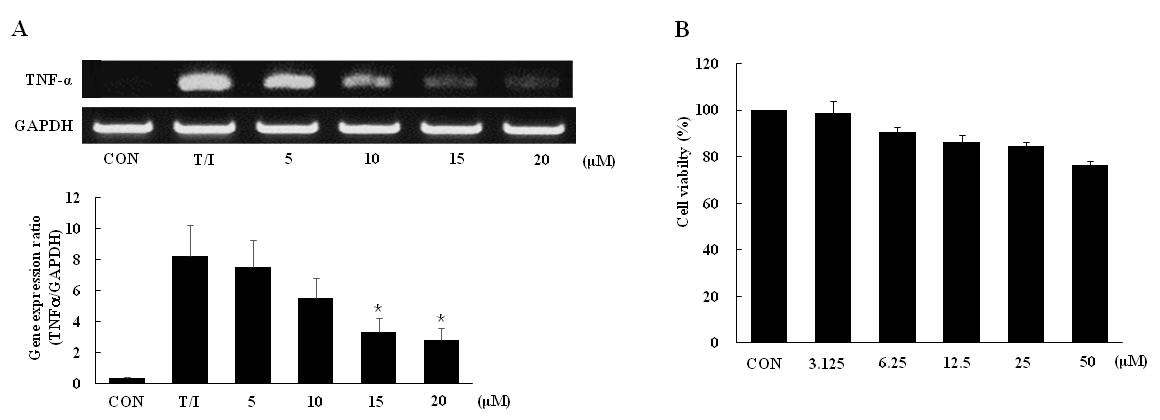


**Figure S8.** (A) Inhibition of **DL-6** against the TNF-*α* expression in HaCaT cells by conventional RT-PCR. ^*^Significant difference from the T/I value at *p* < 0.05. CON: control; T: TNF-*α*; I: IFN-*γ*. (***Full-length blots are given in page S10****).* (B) Cytoxicity evaluation of **DL-6** on HaCaT cells. **DL-6** was treated with HaCaT cells in various concentrations for 48 h followed by MTT assay, and absorbance was measured. Cell viability was calculated as the relative absorbance compared to DMSO vehicle control

| Compounds | Control | 150 mM  NaCl | 4.5 mM KCl | 6 µM NH_4_Cl | 1 mM MgCl_2_ | 2.5 mM CaCl_2_ | 4 µM FeCl_3_ |
| --- | --- | --- | --- | --- | --- | --- | --- |
| **DG-5** | 5 | 10 | 10 | 10 | 10 | 10 | 10 |
| **DG-6** | 10 | 20 | 20 | 10 | 10 | 10 | 10 |
| **DL-5** | 5 | 20 | 10 | 10 | 10 | 10 | 10 |
| **DL-6** | 5 | 20 | 20 | 20 | 20 | 20 | 20 |
| melittin | 5 | 5 | 5 | 10 | 5 | 10 | 10 |

**Table S1.** MIC values of the tested compounds in the presence of physiological salts against *E. coli.*

**Full-length blots**

**Full-length blots of *Figure 4c***

**
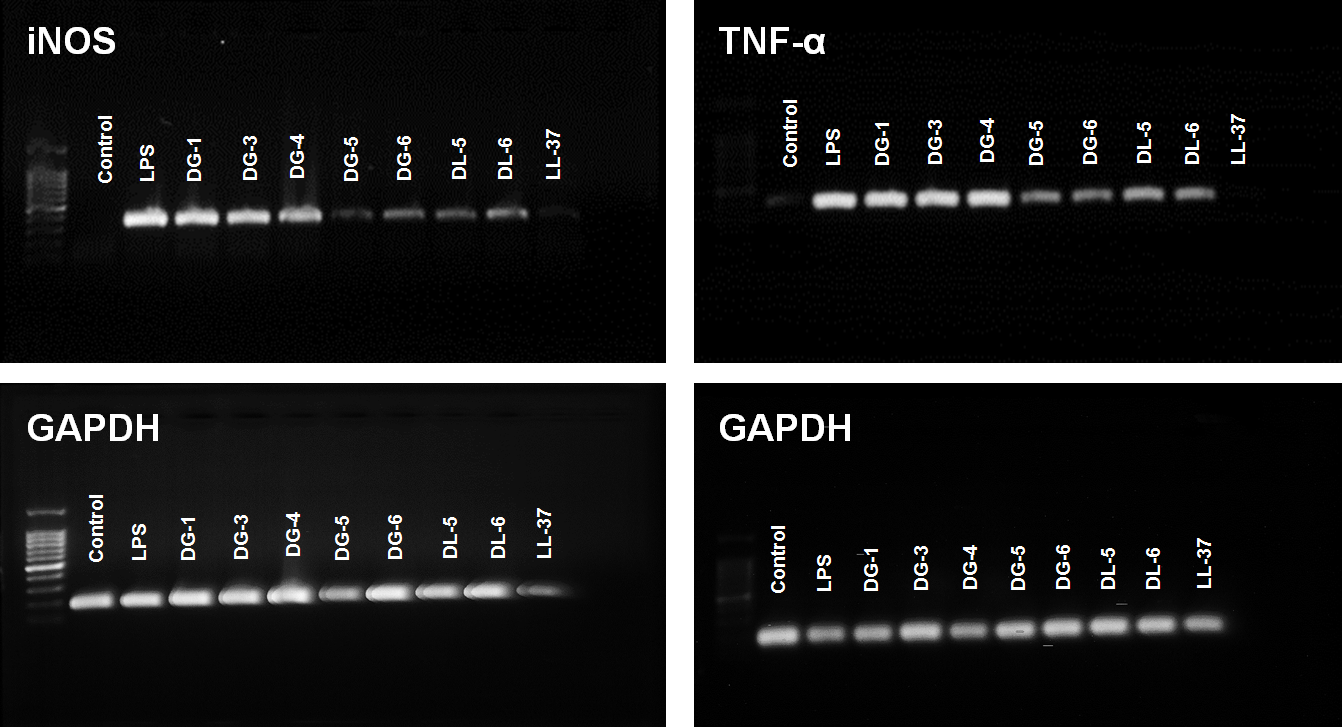
**


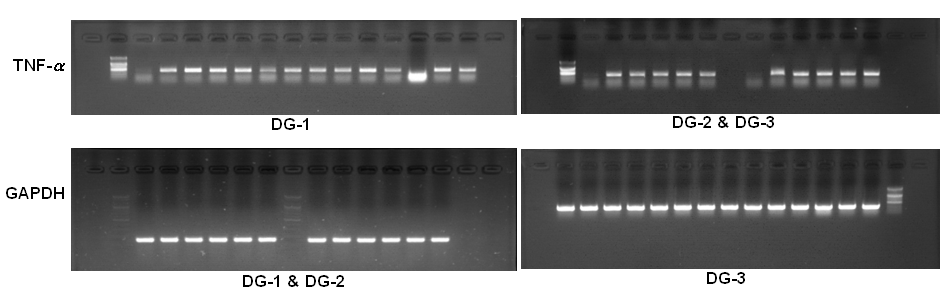
**Full-length blots of DG compounds in *Fig. S7A***


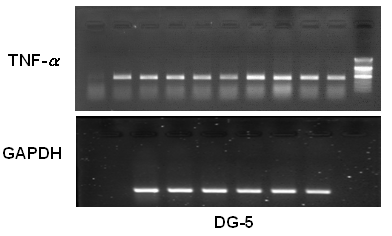


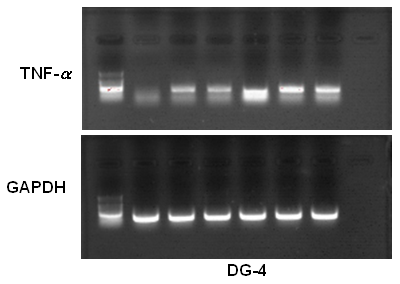


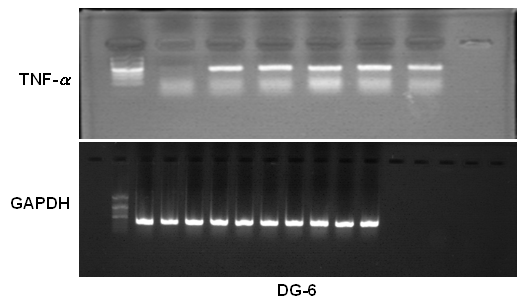


**Full-length blots of DL compounds in *Fig. S7B***

**
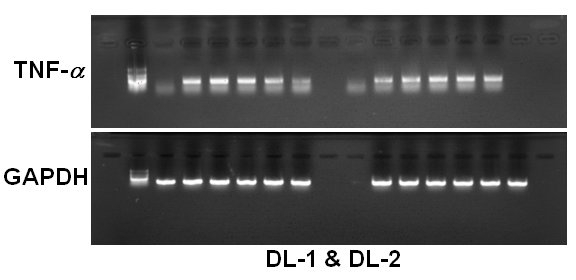
**


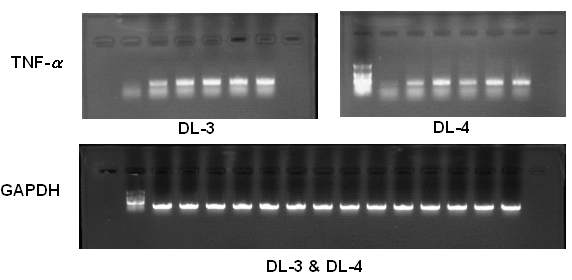


**
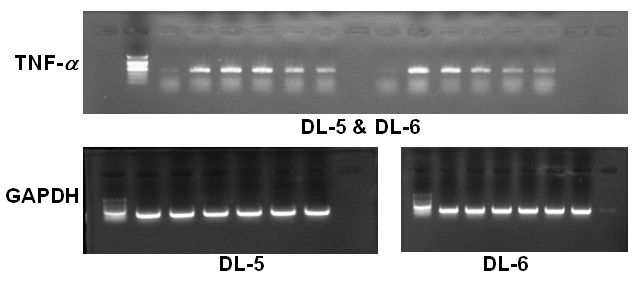
**

**Full-length blots of DL-6 in *Fig. S8A***

***
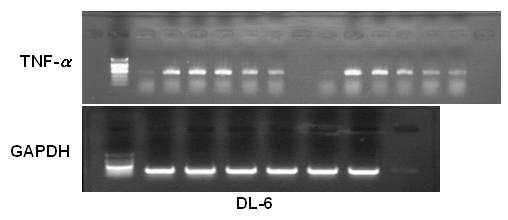
***

**Experimental section**

**Synthesis of 3**

*N,N’*-di-Boc-*N-*trifylguanidine (**2**) (4.27 g, 0.011 mol, 2.05 equiv.) in anhydrous DCM (30 mL) was added slowly to a stirred solution of norspermidine (**1**) (0.7 g, 0.005 mol, 2.05 equiv.) and triethylamine (3.0 mL, 0.021 mol, 4 equiv.) in dichloromethane (DCM) (20 mL) at 0 °C. After completion of addition, the temperature was slowly raised to room temperature and stirred for 6 h. Then the reaction mixture was quenched by the addition of water (30 mL) and extracted with DCM (30 mL x 3). The combined organic extracts were washed with brine (40 mL), dried over Na_2_SO_4_, and evaporated. The crude product was purified by silica gel column chromatography using DCM:MeOH(methanol):TEA(triethylamine) (20:1:0.1 ) mixture to afford **3** as pale brown color viscous oil (2.25g, 68%). ^1^H NMR (400 MHz, CDCl_3_) δ 11.49 (s, 2H), 8.56 (s, 2H), 3.51 (q, *J* = 6.2 Hz, 4H), 2.67 (t, *J* = 6.7 Hz, 4H), 1.77 (t, *J* = 6.7 Hz, 4H), 1.49 (d, *J* = 4.2 Hz, 36H). ^13^C NMR (100 MHz, CDCl_3_) δ 163.6, 156.2, 153.1, 82.9, 79.1, 47.4, 39.2, 29.2, 28.3, 28.1. Maldi-tof *m/z* calcd for C_28_H_53_N_7_O_8_: 615.3, found 616.3 (M+H)^+^

***Synthesis of 5***

To a stirred of solution of 2,4,6-trichloro-1,3,5-triazine (cyanuric chloride) (**4**) (0.89g, 0.005 mol, 1.2 equiv.) and DIEA (2.2 mL, 0.012 mol, 3 equiv.) in 20 mL DCM at 0 °C, **3** (2.50 g, 0.004 mol, 1 equiv.) in 20 mL DCM was added slowly by ensuring that temperature was maintained at 0 °C. After the addition was completed, further stirred at the same temperature for 3 h. The reaction mixture was quenched with water (30 mL) and extracted from DCM (2 x 20 mL). The combined organic layer extracts were washed with brine (30 mL) and dried over Na_2_SO_4_ and evaporated. The crude product was purified by column chromatography using DCM/MeOH (98:2) combination to obtain the shiny, pale yellow solid as a product **5** (3g, 97% ).^1^H NMR (400 MHz, CDCl_3_) δ 11.48 (s, 2H), 8.44 (s, 2H), 3.66 (t, *J* = 7.1 Hz, 4H), 3.47 (q, *J* = 6.4 Hz, 4H), 1.91 (p, *J* = 6.9 Hz, 4H), 1.50 (d, *J* = 2.4 Hz, 36H). ^13^C NMR (100 MHz, CDCl_3_) δ 170.1, 164.9, 163.5, 156.2, 153.2, 83.2, 79.3, 45.3, 38.0, 28.3, 28.1, 26.9.

**General Procedure A for the synthesis of compound 6a-6f**

Synthesis of **6a**

*n*-Butylamine (**a**) (24.2 mg, 0.330 mmol, 1.05 equiv.) in 10 mL DCM was added to the stirred solution of **5** (240 mg, 0.314 mmol, 1 equiv.) and DIEA (0.11 mL, 0.628 mmol, 2 equiv.) in 10 mL at 0 °C. Then, the temperature was raised to room temperature and stirred for 4 h. The reaction mixture was treated with water (15 mL) and extracted with DCM (30 mL). The combined extracts washed with brine (15 mL), dried over Na_2_SO_4_, and evaporated. The crude residue was column purified using hexane–ethylacetate (4:1) combination to yield the product **6a** as a semi solid (200 mg, 79%).^1^H NMR (400 MHz, CDCl_3_) δ 11.82 – 11.42 (m, 2H), 8.75 – 8.28 (m, 2H), 6.41 (t, *J* = 6.0 Hz, 1H), 3.75 – 3.19 (m, 10H), 1.97 – 1.71 (m, 4H), 1.60 – 1.44 (m, 36H), 1.41 – 1.24 (m, 4H), 0.92 (t, *J* = 7.4 Hz, 3H). ^13^C NMR (101 MHz, CDCl_3_) δ 169.6, 168.7, 166.0, 165.4, 163.6, 156.2, 153.1, 83.2, 79.3, 45.3, 44.7, 43.9, 42.9, 40.7, 38.8, 38.1, 37.2, 32.1, 31.4, 29.7, 28.3, 27.5, 20.0, 13.9.

*Synthesis of* ***6b***

The compound **6b** was synthesized by following the *general procedure A*, using cyclohexyl amine (**b**) (31 mg, 0.314 mmol) and **5** (240 mg, 0.314 mmol) in the presence of DIEA (0.11 mL) in DCM as a colorless semi solid (89 mg, 68%).^1^H NMR (400 MHz, CDCl_3_) δ 11.79 – 11.32 (m, 2H), 8.70 – 8.30 (m, 2H), 6.01 (d, *J* = 8.8 Hz, 1H), 3.98 – 3.68 (m, 1H), 3.68 – 3.23 (m, 8H), 2.04 – 1.83 (m, 5H), 1.81 – 1.71 (m, 3H), 1.62 – 1.42 (m, 36H), 1.41 – 1.32 (m, 2H), 1.27 – 1.16 (m, 4H). ^13^C NMR (101 MHz, CDCl_3_) δ 169.6, 168.8, 165.2, 164.5, 163.6, 156.2, 153.1, 83.0, 79.3, 49.2, 45.5, 44.8, 44.0, 42.8, 38.9, 38.1, 37.3, 33.5, 32.5, 31.6, 29.7, 28.3, 27.5, 25.1, 22.6.

*Synthesis of* ***6c****.*

The compound **6c** was synthesized by following the *general procedure A*, using phenylethylamine (**c**) (50 mg, 0.407 mmol ) and **5** (300 mg, 0.394 mmol) in the presence of DIEA (0.140 mL) in DCM as a colorless semi solid (220 mg, 66%). ^1^H NMR (400 MHz, CDCl_3_) δ 11.79 – 11.40 (m, 2H), 8.75 – 8.33 (m, 2H), 7.28 – 7.09 (m, 5H), 6.55 (t, *J* = 6.4 Hz, 1H), 3.69 – 3.53 (m, 6H), 3.51 – 3.35 (m, 4H), 2.86 (q, *J* = 6.4 Hz, 2H), 2.00 – 1.67 (m, 4H), 1.54 – 1.48 (m, 28H), 1.44 (s, 5H), 1.26 (s, 3H). ^13^C NMR (101 MHz, CDCl_3_) δ 169.7, 168.7, 166.0, 165.5, 165.0, 163.6, 156.2, 153.2, 139.0, 128.9, 128.4, 126.4, 83.0, 79.2, 45.3, 44.7, 43.9, 42.5, 38.9, 38.1, 36.9, 36.1, 35.6, 29.7, 27.5 (ethyl acetate is present in traces). Maldi-tof m*/z* calcd for C_39_H_62_ClN_11_O_8_: 847.4, found 860.3 (M+Na)^+^

*Synthesis of* ***6d****.*

The compound **6d** was synthesized by following the *general procedure A*, using phenybutylamine (**d**) (41 mg, 0.276 mmol ) and **5** (200 mg, 0.263 mmol) in the presence of DIEA (0.1 mL) in DCM as a colorless sticky solid (202 mg, 88%). ^1^H NMR (400 MHz, CDCl_3_) δ 11.83 – 11.37 (m, 2H), 8.74 – 8.24 (m, 2H), 7.37 – 7.22 (m, 3H), 7.17 (t, *J* = 7.4 Hz, 3H), 6.44 (t, *J* = 6.1 Hz, 1H), 3.69 – 3.23 (m, 10H), 2.63 (t, *J* = 7.3 Hz, 2H), 1.95 – 1.71 (m, 4H), 1.69 – 1.57 (m, 4H), 1.50 (s, 24H), 1.47 (s, 8H), 1.27 (s, 4H). ^13^C NMR (101 MHz, CDCl_3_) δ 169.6, 168.9, 166.1, 165.4, 163.6, 156.2, 153.3, 142.1, 128.4, 128.3, 125.8, 83.2, 79.3, 45.3, 44.7, 43.9, 42.9, 40.7, 38.9, 38.2, 37.1, 35.5, 29.7, 29.6, 28.6, 28.1, 27.5 (ethyl acetate is present in traces).

*Synthesis of* ***6e***

The compound **6e** was synthesized by following the *general procedure A*, using 1-naphthylmethylamine (**e**) (52 mg, 0.331 mmol ) and **5** (240 mg, 0.316 mmol) in the presence of DIEA (0.1 mL) in DCM as a colorless sticky solid (202 mg, 76%). ^1^H NMR (400 MHz, CDCl_3_) δ 11.68 – 11.37 (m, 2H), 8.73 (t, *J* = 5.9 Hz, 1H), 8.52-8.46 (m, 1H), 8.00 (t, *J* = 6.0 Hz, 1H), 7.89 – 7.81 (m, 1H), 7.77 (d, *J* = 8.0 Hz, 1H), 7.56 – 7.46 (m, 2H), 7.46 – 7.35 (m, 2H), 6.72 (t, *J* = 5.8 Hz, 1H), 5.18 – 4.91 (m, 2H), 3.69 – 3.07 (m, 8H), 1.98 – 1.80 (m, 2H), 1.81 – 1.68 (m, 2H), 1.50 (s, 27H), 1.39 (s, 3H), 1.26 (s, 1H), 1.10 (s, 5H). ^13^C NMR (101 MHz, CDCl_3_) δ 169.8, 168.9, 166.1, 165.5, 163.6, 156.2, 153.2, 133.8, 133.8, 133.6, 131.4, 128.6, 128.2, 126.4, 125.5, 123.4, 123.0, 83.0, 79.2, 45.4, 44.7, 43.0, 38.6, 38.1, 37.1, 29.7, 28.3, 28.1, 27.7, 26.9 (ethyl acetate is present in traces)

*Synthesis of* ***6f***

The compound **6f** was synthesized by following the *general procedure A*, using 3,3-diphenylpropylamine (**f**) (72 mg, 0.344 mmol ) and **5** (248 mg, 0.328 mmol) in the presence of DIEA (0.10 mL) in DCM as a colorless sticky solid (224 mg, 73 %). ^1^H NMR (400 MHz, CDCl_3_) δ 11.82 – 11.39 (m, 2H), 8.74 – 8.17 (m, 2H), 7.37 – 7.07 (m, 10H), 6.47 (t, *J* = 6.1 Hz, 1H), 4.09 – 3.89 (m, 1H), 3.67 – 3.05 (m, 10H), 2.33 (p, *J* = 7.4 Hz, 2H), 1.86 (t, *J* = 7.0 Hz, 2H), 1.76 (q, *J* = 7.1 Hz, 2H), 1.54 – 1.46 (m, 25H), 1.40 (s, 6H), 1.26 (s, 5H). ^13^C NMR (101 MHz, CDCl_3_) δ 169.6, 168.8, 166.1, 165.4, 163.6, 156.2, 155.7, 153.1, 144.3, 128.5, 127.8, 126.3, 83.2, 79.2, 48.8, 44.6, 44.0, 42.9, 39.8, 38.1, 37.2, 35.5, 34.9, 29.7, 28.1, 27.5, 22.7.

**General Procedure B for the synthesis of compound MG-(1-6)**

*1,1'-(3,3'-(4-(butylamino)-6-chloro-1,3,5-triazin-2-ylazanediyl)bis(propane-3,1-diyl)) diguanidine (****MG-1****)*

Compound **6a** (80 mg, 0.100 mmol ) was dissolved in trifluoroacetic acid (TFA)/DCM (9:3 mL) and stirred for 4 h. The reaction mixture was concentrated under vacuo and treated with cold diethyl ether. The precipitate formed was centrifuged and separated. The precipitate was redissolved in CH_3_CN purified by RP-HPLC to give final compound **MG-1** (17.6 mg, 44%) as a white solid. ^1^H NMR (400 MHz, MeOD) δ 3.68 – 3.44 (m, 4H), 3.25 (d, *J* = 7.2 Hz, 2H), 3.18 – 3.04 (m, 4H), 1.94 – 1.72 (m, 4H), 1.56 – 1.37 (m, 2H), 1.29 (p, *J* = 7.4 Hz, 2H), 0.85 (t, *J* = 7.3 Hz, 3H). ^13^C NMR (100 MHz, MeOD) δ 168.3, 165.2, 165.0, 160.9, 157.3, 44.5, 43.9, 40.2, 38.9, 38.4, 31.1, 26.8, 26.5, 19.7, 12.8. Maldi-tof *m/z* calcd for C_15_H_30_ClN_11_: 399.2, found 400.1 (M+H)^+^

*1,1'-(3,3'-(4-chloro-6-(cyclohexylamino)-1,3,5-triazin-2-ylazanediyl)bis(propane-3,1-diyl)) diguanidine (****MG-2****)*

**MG-2** was synthesized from **6b** (59 mg, 0.071 mmol) by following the *general procedure B.* The resultant crude product was purified by RP-HPLC to give final compound **MG-2** (21.7 mg, 72 %) as a white solid. ^1^H NMR (400 MHz, MeOD) δ 3.71 – 3.61 (m, 1H), 3.61 – 3.37 (m, 4H), 3.19 – 3.02 (m, 4H), 1.93 – 1.73 (m, 6H), 1.68 (d, *J* = 12.8 Hz, 2H), 1.55 (d, *J* = 12.0 Hz, 1H), 1.35 – 1.07 (m, 5H). ^13^C NMR (100 MHz, MeOD) δ 169.2, 168.3, 164.9, 161.0, 157.3, 49.9, 49.4, 47.0, 44.7, 44.0, 38.4, 32.1, 26.7, 25.3, 24.7, 24.3. Maldi-tof *m/z* calcd for C_17_H_32_ClN_11_: 425.2, found 426.1 (M+H)^+^

*1,1'-(3,3'-(4-chloro-6-(phenethylamino)-1,3,5-triazin-2-ylazanediyl)bis(propane-3,1-diyl)) diguanidine* *(****MG-3****)*

**MG-3** was synthesized from **6c** (90 mg, 0.106 mmol) was synthesized by following the *general procedure B*. The resultant crude product was purified by RP-HPLC to give final compound **MG-3** (43 mg, 90 %) as a white solid. ^1^H NMR (400 MHz, MeOD) δ 7.24 – 7.03 (m, 5H), 3.63 – 3.40 (m, 6H), 3.11 (q, *J* = 6.7 Hz, 4H), 2.85 – 2.71 (m, 2H), 1.90 – 1.70 (m, 4H). ^13^C NMR (100 MHz, MeOD) δ 169.2, 168.3, 164.8, 161.1, 157.3, 139.2, 128.5, 128.1, 126.0, 44.6, 44.0, 43.6, 42.0, 38.9, 38.4, 35.2, 26.5. Maldi-tof *m/z* calcd for C_19_H_30_ClN_11_: 447.2, found 448.2 (M+H)^+^

*1,1'-(3,3'-(4-chloro-6-(4-phenylbutylamino)-1,3,5-triazin-2-ylazanediyl)bis(propane-3,1-diyl)) diguanidine (****MG-4****)*

**MG-4** was synthesized from **6d** (110 mg, 0.125 mmol) was synthesized by following the *general procedure B*. The resultant crude product was purified by RP-HPLC to give final compound **MG-4** (55 mg, 92 %) as a white solid. ^1^H NMR (400 MHz, MeOD) δ 7.26 (t, *J* = 7.5 Hz, 2H), 7.22 – 7.10 (m, 3H), 3.77 – 3.55 (m, 4H), 3.39 (t, *J* = 6.5 Hz, 2H), 3.22 (t, *J* = 6.9 Hz, 4H), 2.66 (t, *J* = 7.2 Hz, 2H), 1.93 (dq, *J* = 14.6, 7.0 Hz, 4H), 1.75 – 1.55 (m, 4H). ^13^C NMR (100 MHz, MeOD) δ 169.2, 168.1, 164.8, 161.0, 157.3, 142.1, 128.0, 127.9, 125.4, 44.6, 44.0, 40.3, 38.9, 38.4, 35.2, 28.8, 28.4, 26.8, 26.5. Maldi-tof *m/z* calcd for C_21_H_34_ClN_11_: 475.2, found 476.1 (M+H)^+^

*1,1'-(3,3'-(4-chloro-6-(naphthalen-1-ylmethylamino)-1,3,5-triazin-2-ylazanediyl)bis (propane-3,1-diyl))diguanidine* *(****MG-5****)*

**MG-5** was synthesized from **6e** (80 mg, 0.090 mmol) was synthesized by following the *general procedure B*. The resultant crude product was purified by RP-HPLC to give final compound **MG-5** (36mg, 82 %) as a white solid. ^1^H NMR (400 MHz, MeOD) δ 8.00 (d, *J* = 8.1 Hz, 1H), 7.86 – 7.77 (m, 1H), 7.76 – 7.66 (m, 1H), 7.52 – 7.29 (m, 4H), 4.92 (d, *J* = 3.1 Hz, 2H), 3.50 (q, *J* = 8.3, 7.1 Hz, 3H), 3.38 (t, *J* = 7.3 Hz, 1H), 3.08 (t, *J* = 6.8 Hz, 3H), 2.65 (t, *J* = 7.3 Hz, 1H), 1.77 (dq, *J* = 13.1, 6.5 Hz, 3H), 1.54 (p, *J* = 7.0 Hz, 1H). ^13^C NMR (100 MHz, MeOD) δ 168.6, 165.3, 164.9, 160.6, 157.3, 133.9, 131.1, 128.5, 127.5, 125.9, 125.5, 125.1, 124.4, 122.7, 117.8, 44.5, 44.0, 42.1, 38.4, 26.8, 26.2. Maldi-tof *m/z* calcd for C_22_H_30_ClN_11_: 483.2, found 484.2 (M+H)^+^

*1,1'-(3,3'-(4-chloro-6-(3,3-diphenylpropylamino)-1,3,5-triazin-2-ylazanediyl)bis(propane-3,1-diyl))diguanidine (****MG-6****)*

**MG-6** was synthesized from **6f** (100 mg, 0.106 mmol) was synthesized by following the *general procedure B*. The resultant crude product was purified by RP-HPLC to give final compound **MG-6** (45 mg, 78 %) as a white solid. ^1^H NMR (500 MHz, MeOD) δ ^1^H NMR (400 MHz, MeOD) δ 7.16 (d, *J* = 5.0 Hz, 8H), 7.09 – 7.02 (m, 2H), 3.91 (t, *J* = 7.8 Hz, 1H), 3.48 (t, *J* = 7.1 Hz, 3H), 3.37 (t, *J* = 7.0 Hz, 1H), 3.24 (d, *J* = 7.1 Hz, 1H), 3.21 – 3.16 (m, 1H), 3.09 (t, *J* = 6.7 Hz, 2H), 2.94 (t, *J* = 7.2 Hz, 2H), 2.24 (p, *J* = 6.8, Hz, 2H), 1.85 – 1.67 (m, 4H). ^13^C NMR (101 MHz, MeOD) δ 169.2, 168.3, 165.1, 164.8, 157.3, 144.6, 128.1, 128.1, 127.5, 125.9, 48.8, 48.5, 44.3, 43.9, 39.1, 38.4, 34.5, 26.8, 26.4. Maldi-tof *m/z* calcd for C_26_H_36_ClN_11_: 537.3, found 538.3 (M+H)^+^

*Synthesis of tert-butyl 3,3'-azanediylbis(propane-3,1-diyl) dicarbamate (****7****)*

To a stirred solution of 3,3’-diaminodipropylamine ( 7.05 mL, 0.051 mol, 1.0 equiv.) and DIEA (25 mL, 0.142 mol, 2.8 equiv.) in tetrahydrofuran (THF) (125 mL) at 0 ^o^C, was added 1-(tert-butoxycarbonyloxyimino)-2-phenylacetonitlie (BOC-ON) ( 25g, 0.100 mol, 2.0 equiv.) in THF (75 mL) drop wise over 60 minutes. After the addition, the resultant mixture was stirred at 0 ^o^C for three hours and warmed to room temperature, and stirred for 20 h at room temperature. The solvent was evaporated under vacuo, the resultant crude residue was redissolved in DCM (100 mL) and washed with 10% NaOH (3x60 mL) followed by brine (100 mL) and dried over Na_2_SO_4._ The oily product formed after evaporation was treated with hexane (125 mL) and methanol (1 mL) and kept in the freezer for overnight. The resultant white solid was filtered and washed with hexane, and dried to yield the product **7** as a white solid. (13 g, 81%). ^1^H NMR (400 MHz, CDCl_3_) δ 5.19 (s, 2H), 3.21 (q, *J* = 6.3 Hz, 4H), 2.65 (t, *J* = 6.5 Hz, 4H), 1.65 (p, *J* = 6.5 Hz, 4H), 1.44 (s, 18H). ^13^C NMR (100 MHz, CDCl_3_) δ 156.1, 78.9, 47.4, 38.9, 29.8. Maldi-tof *m/z* calcd for C_16_H_33_N_3_O_4_: 331.2, found 332.2 (M+H)^+^

*Synthesis of tert-butyl 3,3'-(4,6-dichloro-1,3,5-triazin-2-ylazanediyl)bis(propane-3,1-diyl)dicarbamate (****8****)*

To a stirred solution of **4** (1 g, 5.43 mmol, 1 equiv.) and DIEA (2.8 mL, 16.3 mmol, 3 equiv.) in DCM (30 mL) at 0 ^o^C, was added **7** (1.83g, 5.54 mmol, 1.02 equiv.) in DCM (20 mL) drop wise over 30 min, ensuring that the temperature was maintained at 0 ^o^C, and stirred further at 0 ^o^C for 3 h. The reaction mixture was quenched with water (50 mL) and extracted from DCM (2 x 30 mL). The combined organic layer extracts were washed with brine (50 mL) and dried over Na_2_SO_4_ and evaporated. The resultant residue was purified by silica gel column chromatography (Hexane-Ethyl acetate, 3:1) to afford **8** as a white solid (2.26g, 87%). ^1^H NMR (400 MHz, CDCl_3_) δ 5.05 (t, *J* = 6.0 Hz, 2H), 3.63 (t, *J* = 6.9 Hz, 4H), 3.13 (q, *J* = 6.4 Hz, 4H), 1.81 (p, *J* = 6.7 Hz, 4H), 1.45 (s, 18H). ^13^C NMR (100 MHz, CDCl_3_) δ 170.1, 164.7, 156.0, 79.3, 45.0, 37.4, 28.4, 27.7. Maldi-tof *m/z* calcd for C_19_H_32_Cl_2_N_6_O_4_: 478.1, found 501.2 (M+Na)^+^

**General procedure C for the synthesis of 9(a-f)**

*synthesis of* *tert-butyl 3,3'-(4,6-bis(4-phenylbutylamino)-1,3,5-triazin-2-ylazanediyl)bis (propane-3,1-diyl)dicarbamate (****9a****)*

Phenylbutylamine **(a)** (0.151 mL, 1.014 mmol, 3.00 equiv.) in 1,4-dioxane (10 mL) was added slowly to the stirred solution of **8** (162 mg, 0.338 mmol, 1 equiv.) and DIEA (0.175 ml, 1.014 mmol, 3 equiv.) in 20 mL 1,4-dioxane and the reaction mixture was stirred at room temperature for an hour. Then, the temperature was increased to reflux condition for 13 h. The solvent was evaporated under vacuo and treated with DCM (40 mL) and water (30 mL). The organic extract was washed with brine and the resultant residue was purified by silica gel column chromatography (Hexane-Ethyl acetate, 7:3) to afford **9a** as a viscous liquid (176 mg, 74 %). ^1^H NMR (400 MHz, CDCl_3_) δ 7.29 (d, *J* = 6.6 Hz, 5H), 7.19 (t, *J* = 6.6 Hz, 5H), 5.33 – 4.76 (m, 2H), 3.66 – 3.47 (m, 4H), 3.48 – 3.33 (m, 4H), 3.08 (q, *J* = 6.3 Hz, 4H), 2.66 (t, *J* = 7.5 Hz, 4H), 2.17 – 1.99 (m, 2H), 1.72 (t, *J* = 7.5 Hz, 8H), 1.67 – 1.57 (m, 4H), 1.46 (s, 18H). ^13^C NMR (100 MHz, CDCl_3_) δ 195.6, 165.2, 156.0, 142.2, 128.4, 128.4, 128.3, 125.8, 78.9, 43.5, 42.2, 40.6, 36.9, 35.6, 31.5, 28.5, 27.5, 25.6.

*synthesis of* *tert-butyl 3,3'-(4,6-bis(butylamino)-1,3,5-triazin-2-ylazanediyl)bis(propane-3,1-diyl)dicarbamate (****9b****)*

Synthesis of **9b** was synthesized from *n*-butylamine **(b)** (0.74 mg, 1.023 mmol, 3.00 equiv.), **8** (162 mg, 0.314 mmol, 1 equiv.) and DIEA (0.185 mL, 1.023 mmol, 3 equiv.) in refluxing 1,4-dioxane by following the *general procedure c*. The resultant crude product was purified by silica gel column chromatography (Hexane-Ethyl acetate, 3:1) to afford **9b** as a viscous liquid (175 mg, 93 %). ^1^H NMR (500 MHz, CDCl_3_) δ 4.81 (s, 2H), 3.57 (s, 4H), 3.41 (s, 4H), 3.09 (s, 4H), 1.73 (s, 4H), 1.58 (dt, *J* = 14.1, 6.6 Hz, 4H), 1.48 (s, 18H), 1.54 – 1.43 (m, 5H), 0.96 (t, *J* = 7.3 Hz, 6H). ^13^C NMR (100 MHz, CDCl_3_) δ 174.0, 165.7, 156.0, 78.8, 42.2, 40.4, 36.8, 32.0, 28.5, 28.4, 27.5, 20.1, 13.9.

*Synthesis of* *tert-butyl 3,3'-(4,6-bis(cyclohexylamino)-1,3,5-triazin-2-ylazanediyl)bis(propane-3,1-diyl)dicarbamate (****9c****)*

Synthesis of **9c** was synthesized from cyclohexylamine **(c)** (0.137 mg, 1.386 mmol, 3.00 equiv.), **8** (220 mg, 0.462 mmol, 1 equiv.) and DIEA (0.245 mL, 1.386 mmol, 3 equiv.) in refluxing 1,4-dioxane by following the *general procedure c*. The resultant crude product was purified by silica gel column chromatography (Hexane-Ethyl acetate, 3:1) to afford **9c** as a brown oil (193 mg, 69 %). ^1^H NMR (500 MHz, CDCl_3_) δ 4.71 (s, 2H), 3.84 – 3.77 (m, 2H), 3.64 – 3.40 (m, 4H), 3.23 – 2.93 (m, 4H), 2.06 – 1.91 (m, 4H), 1.87 – 1.61 (m, 10H), 1.54 – 1.43 (m, 18H), 1.39 (q, *J* = 12.8, 12.4 Hz, 5H), 1.25 – 1.05 (m, 5H). ^13^C NMR (100 MHz, CDCl_3_) δ 165.1, 156.0, 79.0, 72.8, 49.3, 49.1, 36.4, 34.0, 33.5, 28.6, 27.4, 25.7, 24.7.

*Synthesis of* *tert-butyl 3,3'-(4,6-bis(phenethylamino)-1,3,5-triazin-2-ylazanediyl)bis(propane-3,1-diyl)dicarbamate (****9d****)*

Synthesis of **9d** was synthesized from phenethylamine **(d)** (0.167 mg, 1.329 mmol, 3 equiv.), **8** (211 mg, 0.443 mmol, 1 equiv.) and DIEA (0.245 mL, 1.329 mmol, 3 equiv.) in refluxing 1,4-dioxane by following the *general procedure c*. The resultant crude product was purified by silica gel column chromatography (Hexane-Ethyl acetate, 3:1) to afford **9d** as a brown oil (242 mg, 85 %). ^1^H NMR (500 MHz, CDCl_3_) δ 7.32 (t, *J* = 7.5 Hz, 4H), 7.24 (t, *J* = 7.2 Hz, 6H), 6.88 (s, 1H), 5.21 (s, 1H), 4.91 (s, 1H), 3.77 – 3.45 (m, 8H), 3.23 – 3.02 (m, 4H), 3.01 – 2.80 (m, 4H), 1.99 (s, 1H), 1.85 – 1.64 (m, 4H), 1.44 (s, 18H). ^13^C NMR (100 MHz, CDCl_3_) δ 165.8, 156.0, 139.3, 128.8, 128.6, 126.4, 79.4, 51.8, 42.5, 29.9, 28.5, 27.4, 25.6 (ethyl acetate is present in trace).

*Synthesis of* *tert-butyl 3,3'-(4,6-bis(3,3-diphenylpropylamino)-1,3,5-triazin-2-ylazanediyl) bis(propane-3,1-diyl)dicarbamate (****9e****)*

Synthesis of **9e** was synthesized from diphenylpropylamine **(e)** (0.660 mg, 3.131 mmol, 3 equiv.), **8** (500 mg, 1.043 mmol, 1 equiv.) and DIEA (0.577 mL, 3.131 mmol, 3 equiv.) in refluxing 1,4-dioxane by following the *general procedure c*. The resultant crude product was purified by silica gel column chromatography (Hexane-Ethyl acetate, 3:1) to afford **9e** as a white solid (765 mg, 85 %). ^1^H NMR (400 MHz, CDCl_3_) δ 7.26 – 7.04 (m, 20H), 6.77 (s, 1H), 4.91 (s, 2H), 4.00 – 3.83 (m, 2H), 3.48 – 3.30 (m, 4H), 3.30 – 3.12 (m, 4H), 3.04 – 2.84 (m, 4H), 2.25 (q, *J* = 7.3 Hz, 4H), 1.68 – 1.51 (m, 4H), 1.45 – 1.26 (m, 18H). ^13^C NMR (100 MHz, CDCl_3_) δ 165.9, 156.0, 144.5, 128.5, 127.9, 126.3, 78.8, 48.6, 42.6, 39.3, 37.0, 35.7, 28.5, 27.7 (ethyl acetate is present in trace). Maldi-tof m*/z* calcd for C_49_H_64_N_8_O_4_: 828.1, found 829.0 (M+H)^+^ , 851.0 (M+Na)^+^ .

*Synthesis of* *tert-butyl 3,3'-(4,6-bis(naphthalen-1-ylmethylamino)-1,3,5-triazin-2-*

*ylazanediyl)bis(propane-3,1-diyl)dicarbamate (****9f****)*

Synthesis of **9f** was synthesized from 1-naphthylmethylamine **(f)** (0.491 mg, 3.131 mmol, 3. equiv.), **8** (500 mg, 1.043 mmol, 1 equiv.) and DIEA (0.577 mL, 3.131 mmol, 3 equiv.) in refluxing 1,4-dioxane by following the *general procedure c*. The resultant crude product was purified by silica gel column chromatography (Hexane-Ethyl acetate, 3:1) to afford **9f** as a white solid (640 mg, 86 %). ^1^H NMR (500 MHz, CDCl_3_) δ 8.16 – 8.04 (m, 2H), 7.94 – 7.85 (m, 2H), 7.81 (d, *J* = 8.0 Hz, 2H), 7.61 – 7.37 (m, 8H), 6.77 (s, 1H), 5.47 – 5.15 (m, 2H), 5.11 (s, 4H), 3.67 – 3.43 (m, 4H), 3.19 – 2.90 (m, 4H), 1.86 – 1.73 (m, 4H), 1.40 – 1.15 (m, 18H). ^13^C NMR (100 MHz, CDCl_3_) δ 193.6, 165.9, 165.5, 156.0, 134.4, 133.8, 131.5, 128.7, 128.1, 126.4, 125.8, 125.4, 123.5, 78.7, 42.7, 36.7, 29.7, 27.5.

***General Procedure D* for the synthesis of compound DL-(1-6)**

*Synthesis of N^2^,N^2^-bis(3-aminopropyl)-N^4^,N^6^-bis(4-phenylbutyl)-1,3,5-triazine-2,4,6-triamine (****DL-1)***

Compound **9a** (210 mg, 0.298 mmol ) was dissolved in TFA/DCM (7.5:2.5 mL) and stirred for 4 h. The reaction mixture was concentrated under vacuo and treated with cold diethyl ether. The precipitate formed was centrifuged and separated. The precipitate was redissolved in CH_3_CN purified by RP-HPLC to result **DL-1** (110 mg, 73 %) as a pale brown solid. ^1^H NMR (500 MHz, MeOD) δ 7.27 (t, *J* = 7.4 Hz, 4H), 7.21 (d, *J* = 7.6 Hz, 4H), 7.17 (t, *J* = 7.4 Hz, 2H), 4.16 (s, 1H), 3.86 – 3.64 (m, 5H), 3.55 – 3.35 (m, 4H), 3.16 – 2.92 (m, 5H), 2.69 (t, *J* = 7.2 Hz, 4H), 2.05 (d, *J* = 9.2 Hz, 4H), 1.80 – 1.57 (m, 8H). ^13^C NMR (100 MHz, MeOD) δ 161.0, 154.4, 142.0, 128.2, 128.0, 125.5, 56.3, 44.9, 40.6, 37.4, 35.1, 28.5, 25.8. Maldi-tof *m/z* calcd for C_29_H_44_N_8_: 504.3, found 505.2 (M+H)^+^

*Synthesis of N^2^,N^2^-bis(3-aminopropyl)-N^4^,N^6^-dibutyl-1,3,5-triazine-2,4,6-triamine* ***(DL-2)***

**DL-2** was synthesized from **9b** (68 mg, 0.298 mmol) and TFA/DCM (3:1) following the *general procedure D* as a white solid (31 mg, 86%). ^1^H NMR (500 MHz, MeOD) δ 3.78 (t, *J* = 7.2 Hz, 4H), 3.46 (t, *J* = 6.9 Hz, 3H), 3.37 (d, *J* = 5.3 Hz, 1H), 3.02 (t, *J* = 7.8 Hz, 4H), 2.16 – 1.99 (m, 4H), 1.64 (p, *J* = 7.1 Hz, 4H), 1.45 (h, *J* = 6.9 Hz, 4H), 1.00 (t, *J* = 7.3 Hz, 6H). ^13^C NMR (100 MHz, MeOD) δ 162.8, 154.5, 44.8, 40.4, 37.4, 30.7, 25.8, 19.7, 12.8. Maldi-tof m*/z* calcd for C_17_H_36_N_8_: 352.3, found 353.1 (M+H)^+^

*Synthesis of N^2^,N^2^-bis(3-aminopropyl)-N^4^,N^6^-dicyclohexyl-1,3,5-triazine-2,4,6-triamine*

***(DL-3)***

**DL-3** was synthesized from **9c** (57 mg, 0.095 mmol) and TFA/DCM (3:1) following the *general procedure D* as a white solid (32 mg, 83%). ^1^H NMR (500 MHz, MeOD) δ 4.00 – 3.59 (m, 6H), 3.02 (t, *J* = 7.7 Hz, 4H), 2.15 – 2.03 (m, 4H), 2.00 (dd, *J* = 10.9, 5.9 Hz, 4H), 1.89 – 1.77 (m, 4H), 1.74 – 1.59 (m, 2H), 1.54 – 1.22 (m, 10H). ^13^C NMR (100 MHz, MeOD) δ 162.7, 153.6, 50.1, 44.9, 37.5, 31.9, 25.9, 25.2, 24.3. Maldi-tof m*/z* calcd for C_21_H_40_N_8_: 404.3, found 405.2 (M+H)^+^

*Synthesis of N^2^,N^2^-bis(3-aminopropyl)-N^4^,N^6^-diphenethyl-1,3,5-triazine-2,4,6-triamine* ***(DL-4)***

**DL-4** was synthesized from **9d** (73 mg, 0.113 mmol) and TFA/DCM (3:1) following the *general procedure D* as a white solid (51 mg, 84%). ^1^H NMR (500 MHz, MeOD) δ 7.37 – 7.26 (m, 8H), 7.24 (t, *J* = 7.3 Hz, 2H), 3.73 (dt, *J* = 20.1, 7.0 Hz, 8H), 3.00 (t, *J* = 8.0 Hz, 4H), 2.95 (t, *J* = 7.0 Hz, 4H), 2.13 – 2.00 (m, 4H). ^13^C NMR (100 MHz, MeOD) δ 162.2, 157.3, 154.5, 138.6, 128.6, 128.3, 126.3, 45.5, 41.9, 39.0, 34.9, 26.8. Maldi-tof m*/z* calcd for C_25_H_36_N_8_: 448.3, found 449.2 (M+H)^+^

*Synthesis of N^2^,N^2^-bis(3-aminopropyl)-N^4^,N^6^-bis(3,3-diphenylpropyl)-1,3,5-triazine-2,4,6-triamine*  ***(DL-5)***

**DL-5** was synthesized from **9e** (77 mg, 0.092 mmol) and TFA/DCM (3:1) following the *general procedure D* as a white solid (58 mg, 72%). ^1^H NMR (400 MHz, MeOD) δ 7.26 – 7.12 (m, 16H), 7.07 (d, *J* = 7.5 Hz, 4H), 3.96 (t, *J* = 7.7 Hz, 2H), 3.63 – 3.23 (m, 7H), 3.00 – 2.60 (m, 4H), 2.36 – 2.18 (m, 4H), 1.99 – 1.73 (m, 4H). ^13^C NMR (100 MHz, MeOD) δ 162.5, 154.4, 144.4, 128.3, 128.3, 127.5, 126.1, 48.5, 44.5, 39.2, 37.2, 34.4, 25.7. Maldi-tof m*/z* calcd for C_39_H_48_N_8_: 628.4, found 629.4 (M+H)^+^

*Synthesis of N^2^,N^2^-bis(3-aminopropyl)-N^4^,N^6^-bis(naphthalen-1-ylmethyl)-1,3,5-triazine-2,4,6-triamine* ***(DL-6)***

**DL-6** was synthesized from **9f** (66 mg, 0.092 mmol) and TFA/DCM (3:1) following the *general procedure D* as a white solid (39 mg, 81 %). ^1^H NMR (400 MHz, MeOD) δ 8.13 (d, *J* = 8.4 Hz, 2H), 7.96 (d, *J* = 8.0 Hz, 2H), 7.89 (d, *J* = 8.0 Hz, 2H), 7.67 – 7.41 (m, 8H), 5.13 (s, 4H), 3.67 (t, *J* = 7.2 Hz, 4H), 3.10 – 2.95 (m, 1H), 2.72 (t, *J* = 7.5 Hz, 3H), 1.96 (tt, *J* = 15.1, 9.1 Hz, 4H). ^13^C NMR (100 MHz, MeOD) δ 162.6, 154.6, 134.0, 132.4, 131.1, 128.6, 128.2, 126.4, 125.8, 125.5, 125.2, 122.7, 44.8, 42.4, 37.0, 25.5. Maldi-tof m*/z* calcd for C_31_H_36_N_8_: 520.3, found 521.0 (M+H)^+^ (rotomer existence were present in both proton and carbon)

***General Procedure E* for the synthesis of compound 10-(a-e)**

*Synthesis of (****10a)***

Compound **9a** (156 mg, 0.221 mmol) was treated with TFA/DCM (7.5:2.5 mL) and stirred for 4 h. The reaction mixture is concentrated under vacuo and dissolved in anhydrous DCM (20 mL)*.* The reaction mixture is cooled to 0 °C and triethylamine (0.32 mL, 2.21 mmol, 10 equiv.) in DCM (5 mL) was added drop wise. After the addition, *N,N’*-di-Boc-*N*-trifylguanidine (216 mg, 0.552 mmol, 2.5 equiv.) in anhydrous DCM (5 mL) was added to the reaction mixture at 0 °C . The reaction temperature was increased to room temperature and stirred for 6 h. Then the reaction mixture was quenched by the addition of water (20 mL) and extracted with DCM (10 mL x 3). The combined organic extracts were washed with brine (20 mL), dried over Na_2_SO_4_, and evaporated. The resultant crude product was purified by silica gel column chromatography (Hexane-Ethyl acetate, 3:1) to afford **10a** as a colorless liquid (209 mg, 96 %). ^1^H NMR (400 MHz, CDCl_3_) δ ^1^H NMR (400 MHz, CDCl_3_) δ 11.73 (s, 2H), 8.86 (s, 2H), 7.24 (t, *J* = 7.3 Hz, 4H), 7.20 – 7.08 (m, 6H), 6.06 (s, 2H), 3.50 (t, *J* = 5.9 Hz, 4H), 3.46 – 3.26 (m, 8H), 2.61 (t, *J* = 7.3 Hz, 4H), 1.78 – 1.56 (m, 12H), 1.52 – 1.45 (m, 36H). ^13^C NMR (100 MHz, CDCl_3_) δ 166.3, 163.9, 156.7, 155.7, 153.1, 142.4, 128.4, 128.3, 125.7, 83.0, 79.2, 40.5, 35.7, 30.0, 28.9, 28.4, 28.3, 28.1, 27.9. Maldi-tof m*/z* calcd for C_51_H_80_N_12_O_8_: 988.6, found 788.1 (M-200)^+^(2 Boc cleaved)

*Synthesis of (****10b)***

Compound **10b** was synthesized from **9b** (113 mg, 0.205 mmol), TFA/DCM followed by *N,N’*-di-Boc-*N*-trifylguanidine (200 mg, 0.513 mmol, 2.5 equiv) following the G*eneral procedure E* to afford the crude product, which was purified by column chromatography to yield (Hexane-Ethyl acetate, 3:1) to afford **10b** as a colorless liquid (162 mg, 94 %). ^1^H NMR (400 MHz, CDCl_3_) δ 8.85 (s, 2H), 8.39 (s, 1H), 6.02 (s, 1H), 3.63 – 3.25 (m, 12H), 1.93 – 1.65 (m, 4H), 1.54 (s, 9H), 1.53 (s, 9H), 1.50-1.48 (m,27H), 1.35 (h, *J* = 7.4 Hz, 4H), 0.91 (t, *J* = 7.3 Hz, 6H). ^13^C NMR (101 MHz, CDCl_3_) δ 166.1, 163.6, 155.8, 153.2, 83.1, 79.4, 40.4, 37.3, 32.4, 29.7, 28.3, 28.1, 27.6, 20.1, 13.9. Maldi-tof m*/z* calcd for C_39_H_72_N_12_O_8_: 836.56, found 837.0(M+H)^+^, (M-100)^+^(1 Boc cleaved).

*Synthesis of (****10c****)*

Compound **10c** was synthesized from **9c** (115 mg, 0.190 mmol), TFA/DCM followed by *N,N’*-di-Boc-*N*-trifylguanidine (186 mg, 0.477 mmol, 2.5 equiv.) following the G*eneral procedure E* to afford the crude product, which was purified by column chromatography to yield (Hexane-Ethyl acetate, 3:1) to afford **10c** as a colorless liquid (147 mg, 87 %). ^1^H NMR (400 MHz, CDCl_3_) δ 11.67 (s, 2H), 8.87 – 8.26 (m, 2H), 5.56 (s, 2H), 3.90 – 3.63 (m, 2H), 3.63 – 3.20 (m, 8H), 2.11-1.91 (m, 4H), 1.77-1.65 (m, 6H), 1.58 – 1.42 (m, 34H), 1.38 – 1.24 (m, 8H), 1.22 – 1.09 (m, 6H), 0.93 – 0.79 (m, 2H). ^13^C NMR (101 MHz, CDCl_3_) δ 165.4, 163.9, 155.8, 153.0, 82.9, 79.1, 49.6, 41.3, 37.4, 33.6, 29.7, 28.3, 28.2, 27.8, 25.9, 25.4, 22.7, 14.1.

*Synthesis of (****10d****)*

Compound **10d** was synthesized from **9d** (146 mg, 0.226 mmol), TFA/DCM followed by *N,N’*-di-Boc-*N*-trifylguanidine (220 mg, 0.565 mmol, 2.5 equiv) following the G*eneral procedure E* to afford the crude product, which was purified by column chromatography to yield (Hexane-Ethyl acetate, 3:1) to afford **10d** as a white solid (172 mg, 82 %). ^1^H NMR (500 MHz, CDCl_3_) δ 8.87 (s, 2H), 7.29 – 7.21 (m, 4H), 7.19 (d, *J* = 6.9 Hz, 2H), 7.14 (d, *J* = 7.4 Hz, 4H), 6.35 – 5.98 (m, 2H), 3.69 – 3.48 (m, 8H), 3.48 – 3.28 (m, 4H), 2.91 (t, *J* = 7.5 Hz, 4H), 1.78 – 1.61 (m, 4H), 1.51 (d, *J* = 4.2 Hz, 18H), 1.47 (s, 18H). ^13^C NMR (101 MHz, CDCl_3_) δ 166.2, 163.9, 155.7, 153.1, 148.7, 139.8, 128.8, 128.4, 126.2, 82.9, 79.2, 42.7, 41.1, 37.0, 36.7, 29.7, 28.4, 28.1. Maldi-tof m*/z* calcd for C_47_H_72_N_12_O_8_: 932.5, found (M-200)^+^ (2 Boc cleaved)

*Synthesis of* (***10e***)

Compound **10e** was synthesized from **9e** (153 mg, 0.184 mmol), TFA/DCM followed by *N,N’*-di-Boc-*N*-trifylguanidine (181 mg, 0.462 mmol, 2.5 equiv) following the G*eneral procedure E* to afford the crude product, which was purified by column chromatography to yield (Hexane-Ethyl acetate, 3:1) to afford **10e** as a white solid (181 mg, 88 %).^1^H NMR (400 MHz, CDCl_3_) δ 11.72 (s, 2H), 8.84 (s, 2H), 7.28 – 7.19 (m, 11H), 7.15 (d, *J* = 7.4 Hz, 9H), 6.04 (s, 2H), 3.90 (s, 2H), 3.61 – 3.33 (m, 8H), 3.32 – 3.13 (m, 4H), 2.31 (q, *J* = 7.6 Hz, 4H), 1.66 (d, *J* = 19.7 Hz, 6H), 1.50 (s, 18H), 1.39 (s, 16H) ^13^C NMR (100 MHz, CDCl_3_) δ 171.2, 166.3, 163.9, 155.7, 153.1, 144.7, 128.4, 127.8, 126.1, 83.0, 79.2, 60.4, 48.8, 41.3, 39.6, 37.2, 35.8, 28.4, 27.7, 21.1, 14.2. (ethyl acetate is present in traces). Maldi-tof m*/z* calcd for C_61_H_84_N_12_O_8_: 1112.65, found 813.0 (M-300)^+^(3 Boc groups cleaved).

*Synthesis of (****10f****)*

Compound **10f** was synthesized from **9f** (132 mg, 0.182 mmol), TFA/DCM followed by *N,N’*-di-Boc-*N*-trifylguanidine (178 mg, 0.456 mmol, 2.5 equiv) following the G*eneral procedure E* to afford the crude product, which was purified by column chromatography to yield (Hexane-Ethyl acetate, 3:1) to afford **10f** as a white solid (163 mg, 89 %). ^1^H NMR (500 MHz, CDCl_3_) δ 11.64 (s, 2H), 8.93 (d, *J* = 6.0 Hz, 2H), 8.06 (d, *J* = 8.3 Hz, 2H), 7.91 – 7.81 (m, 2H), 7.74 (d, *J* = 8.3 Hz, 2H), 7.56 – 7.31 (m, 8H), 6.42 (d, *J* = 6.3 Hz, 2H), 5.08 (dd, *J* = 17.6, 9.3 Hz, 4H), 3.58 (t, *J* = 6.1 Hz, 4H), 3.52 – 3.29 (m, 4H), 1.90 – 1.66 (m, 4H), 1.56 – 1.50 (m, 18H), 1.31 – 1.26 (m, 4H), 1.23 – 1.19 (m, 11H), 0.93 – 0.90 (m, 3H). (4 Boc groups exist as rotomer) ^13^C NMR (100 MHz, CDCl_3_) δ 166.4, 163.9, 155.7, 153.0, 135.1, 133.7, 131.5, 128.5, 127.7, 126.1, 125.6, 125.5, 125.4, 123.5, 83.0, 79.1, 42.6, 41.4, 37.2, 28.4, 27.8 (dichloromethane present in traces in ^13^C NMR).

***General Procedure F* for the synthesis of compound DG-(1-6)**

Synthesis of *1,1'-(3,3'-(4,6-bis(4-phenylbutylamino)-1,3,5-triazin-2-ylazanediyl)bis(propane-3,1-diyl))diguanidine (****DG-1)***

Compound **10a** (110 mg, 0.111 mmol) was dissolved in TFA/DCM (7.5:2.5 mL) and stirred for 4 h. The reaction mixture was concentrated under vacuo and treated with cold diethyl ether. The precipitate formed was centrifuged and separated. The precipitate was redissolved in CH_3_CN purified by RP-HPLC to result **DG-1** (40 mg, 65 %) as a pale brown solid. ^1^H NMR (500 MHz, MeOD) δ 7.26 (t, *J* = 7.5 Hz, 4H), 7.22 – 7.13 (m, 6H), 3.70 (t, *J* = 7.4 Hz, 4H), 3.45 (d, *J* = 6.7 Hz, 4H), 3.25 (t, *J* = 6.9 Hz, 4H), 2.68 (t, *J* = 7.2 Hz, 4H), 1.96 (p, *J* = 7.1 Hz, 4H), 1.79 – 1.57 (m, 8H). ^13^C NMR (100 MHz, MeOD) δ 162.4, 157.3, 154.4, 142.0, 128.1, 128.0, 125.5, 45.3, 40.4, 38.9, 35.1, 28.4, 28.1, 26.8. Maldi-tof m*/z* calcd for C_31_H_48_N_12_: 588.4, found 589.1 (M+H)^+^

*Synthesis of 2,2'-(3,3'-(4,6-bis(butylamino)-1,3,5-triazin-2-ylazanediyl)bis(propane-3,1-diyl))diguanidine* ***(DG-2)***

**DG-2** was synthesized from **10b** (140 mg, 0.167 mmol) and TFA/DCM (3:1) following the *general procedure F* as a white solid (50 mg, 68%).^1^H NMR (500 MHz, MeOD) δ 7.61 (s, 1H), 3.76 (t, *J* = 7.3 Hz, 4H), 3.45 (t, *J* = 6.8 Hz, 4H), 3.29 (d, *J* = 6.5 Hz, 4H), 2.15 – 1.84 (m, 4H), 1.62 (q, *J* = 7.2 Hz, 4H), 1.44 (q, *J* = 7.5 Hz, 4H), 0.99 (t, *J* = 7.3 Hz, 6H). ^13^C NMR (100 MHz, MeOD) δ 162.5, 157.3, 154.5, 45.4, 40.3, 38.9, 30.8, 26.8, 19.7, 12.8. Maldi-tof m*/z* calcd for C_19_H_40_N_12_: 436.3 found 437.7 (M+H)^+^

*Synthesis of 2,2'-(3,3'-(4,6-bis(cyclohexylamino)-1,3,5-triazin-2-ylazanediyl)bis(propane-3,1-diyl))diguanidine* ***(DG-3)***

**DG-3** was synthesized from **10c** (110 mg, 0.123 mmol) and TFA/DCM (3:1) following the *general procedure F* as a white solid (34 mg, 57%).^1^H NMR (500 MHz, MeOD) δ 3.98 – 3.82 (m, 2H), 3.79 – 3.63 (m, 4H), 3.28 (d, *J* = 6.3 Hz, 4H), 1.99 (t, *J* = 11.0 Hz, 8H), 1.87 – 1.76 (m, 4H), 1.72 – 1.62 (m, 2H), 1.50 – 1.25 (m, 10H). ^13^C NMR (100 MHz, MeOD) δ 162.4, 157.3, 153.5, 50.0, 45.6, 39.0, 31.9, 26.9, 25.1, 24.3. Maldi-tof m*/z* calcd for C_23_H_44_N_12_: 488.3, found 490.8 (M+H)^+^

*Synthesis of 2,2'-(3,3'-(4,6-bis(phenethylamino)-1,3,5-triazin-2-ylazanediyl)bis(propane-3,1-diyl))diguanidine* ***(DG-4)***

**DG-4** was synthesized from **10d** (140 mg, 0.150 mmol) and TFA/DCM (3:1) following the *general procedure F* as a white solid (45 mg, 56 %). ^1^H NMR (500 MHz, MeOD) δ 7.62 (s, 1H), 7.31 (t, *J* = 7.4 Hz, 4H), 7.29 – 7.18 (m, 6H), 3.71 (q, *J* = 7.3 Hz, 8H), 3.31 – 3.19 (m, 4H), 2.94 (t, *J* = 6.9 Hz, 4H), 1.98 (t, *J* = 7.3 Hz, 4H). ^13^C NMR (100 MHz, MeOD) δ 162.2, 157.3, 154.5, 138.6, 128.6, 128.3, 126.3, 45.5, 41.9, 39.0, 34.9, 26.8. Maldi-tof m*/z* calcd for C_27_H_40_N_12_: 532.3, found 533.8 (M+H)^+^

*Synthesis of 2,2'-(3,3'-(4,6-bis(3,3-diphenylpropylamino)-1,3,5-triazin-2-ylazanediyl) bis(propane-3,1-diyl))diguanidine* ***(DG-5)***

**DG-5** was synthesized from **10e** (120 mg, 0.108 mmol) and TFA/DCM (3:1) following the *general procedure F* as a white solid (55.2 mg, 72 %). ^1^H NMR (500 MHz, MeOD) δ 7.30 (d, *J* = 6.8 Hz, 16H), 7.18 (t, *J* = 6.9 Hz, 5H), 4.12 – 4.02 (m, 2H), 3.69 – 3.53 (m, 5H), 3.44 (t, *J* = 7.0 Hz, 4H), 3.12 (t, *J* = 7.1 Hz, 4H), 2.42 (q, *J* = 7.2 Hz, 5H), 2.02 – 1.89 (m, 2H), 1.86 (t, *J* = 7.2 Hz, 4H). ^13^C NMR (100 MHz, MeOD) δ 162.2, 157.2, 154.4, 144.4, 128.2, 127.5, 126.0, 48.5, 45.2, 39.1, 38.9, 34.3, 26.7. Maldi-tof m*/z* calcd for C_41_H_52_N_12_: 712.4, found 713.4 (M+H)^+^ (exist as rotomer)

*Synthesis of 2,2'-(3,3'-(4,6-bis(phenethylamino)-1,3,5-triazin-2-ylazanediyl)bis(propane-3,1-diyl))diguanidine* ***(DG-6)***

**DG-6** was synthesized from **10f** (110 mg, 0.109 mmol) and TFA/DCM (3:1) following the *general procedure F as* a white solid (52 mg, 78 %). ^1^H NMR (500 MHz, MeOD) δ 8.11 (d, *J* = 8.4 Hz, 2H), 7.95 (d, *J* = 8.1 Hz, 2H), 7.88 (d, *J* = 8.2 Hz, 2H), 7.61 (t, *J* = 7.5 Hz, 2H), 7.58 – 7.52 (m, 4H), 7.49 (t, *J* = 7.5 Hz, 2H), 5.16 – 5.09 (m, 4H), 3.67 (t, *J* = 7.4 Hz, 4H), 3.30 – 3.18 (m, 1H), 2.98 (t, *J* = 7.1 Hz, 3H), 1.95 (s, 1H), 1.81 (t, *J* = 7.8 Hz, 4H). ^13^C NMR (100 MHz, MeOD) δ 162.3, 157.1, 154.5, 134.0, 132.5, 131.1, 128.6, 128.2, 126.3, 125.7, 125.5, 125.1, 122.7, 45.4, 42.3, 38.7, 26.6. Maldi-tof m*/z* calcd for C_41_H_52_N_12_: 604.3, found 605.3 (M+H)^+^

***DL-6-FITC***

To the stirred solution of FITC (20mg, 0.0514 mmol, 1 equiv) in DMF (1 mL) at -10 ^o^C, DL-6 (28.1 mg, 0.0539 mmol, 1.05 equiv.) and DIEA (50 µl, 0.2699 mmol, 5 equiv) in DMF(1mL) was added dropwise and stirred for 3 h at -10 ^o^C. The resulting crude was purified using RP-HPLC to achieve the Dl-6-FITC as an orange color solid. ^1^H NMR (400 MHz, MeOD) δ 8.14 – 7.93 (m, 3H), 7.90 – 7.58 (m, 5H), 7.54 – 7.28 (m, 8H), 6.88 (d, *J* = 8.2 Hz, 1H), 6.75 – 6.64 (m, 2H), 6.60 (d, *J* = 8.8 Hz, 2H), 6.52 (dd, *J* = 8.8, 2.4 Hz, 2H), 5.01 (d, *J* = 12.1 Hz, 4H), 3.72 – 3.30 (m, 6H), 2.65 – 2.41 (m, 3H), 1.98 – 1.69 (m, 4H). ^13^C NMR (101 MHz, MeOD) δ 169.3, 162.6, 154.7, 153.7, 141.0, 133.9, 132.4, 131.2, 131.0, 129.5, 129.4, 128.6, 128.5, 128.3, 128.3, 128.2, 128.2, 127.8, 126.4, 126.2, 125.8, 125.7, 125.3, 125.2, 125.1, 124.9, 122.9, 122.6, 113.3, 111.0, 102.1, 74.5, 45.6, 44.7, 42.3, 41.6, 39.0, 36.9, 26.9, 25.6. Maldi-tof m*/z* calcd for C_53_H_49_N_9_O_4_S:907.3, found 908.3(M+H)^+^


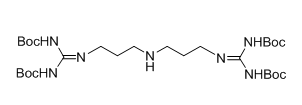

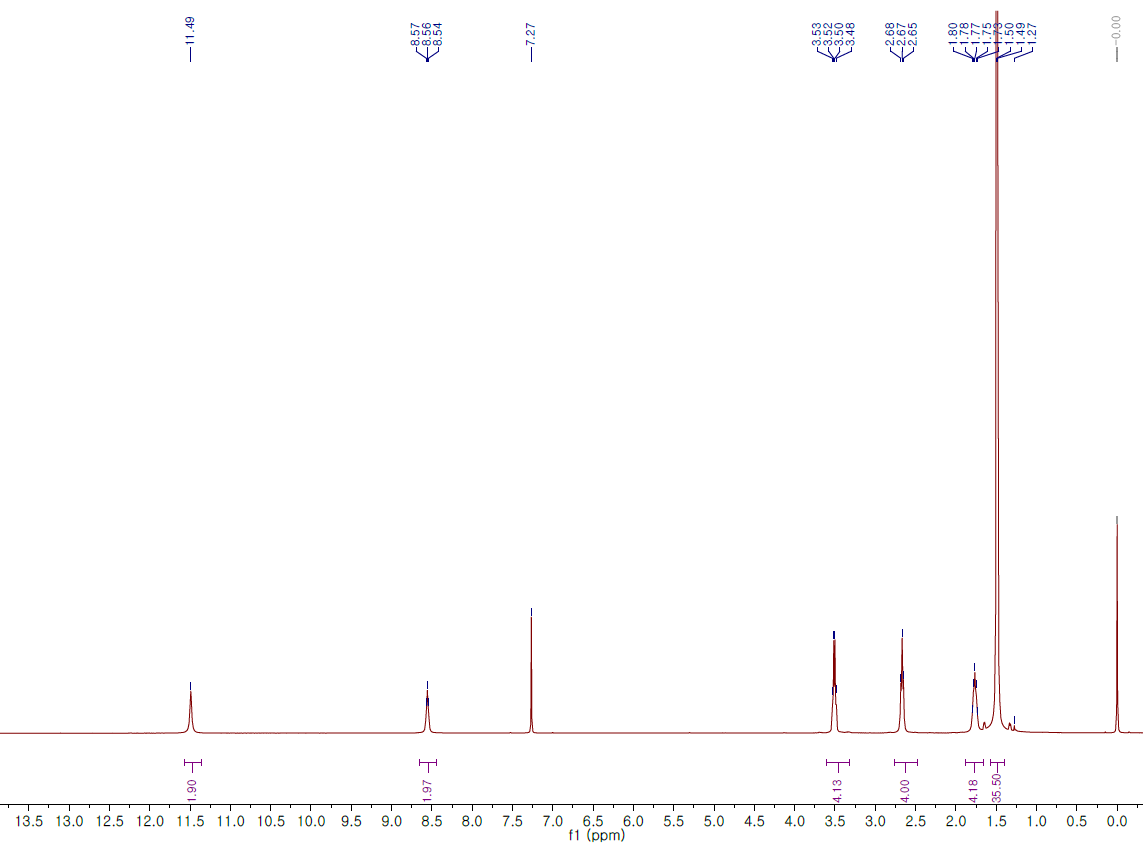


^1^H NMR spectrum of **3** (CDCl_3_, 400 MHz)


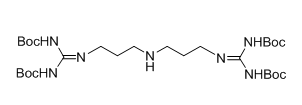


^13^C NMR spectrum of **3** (CDCl_3_, 101 MHz)


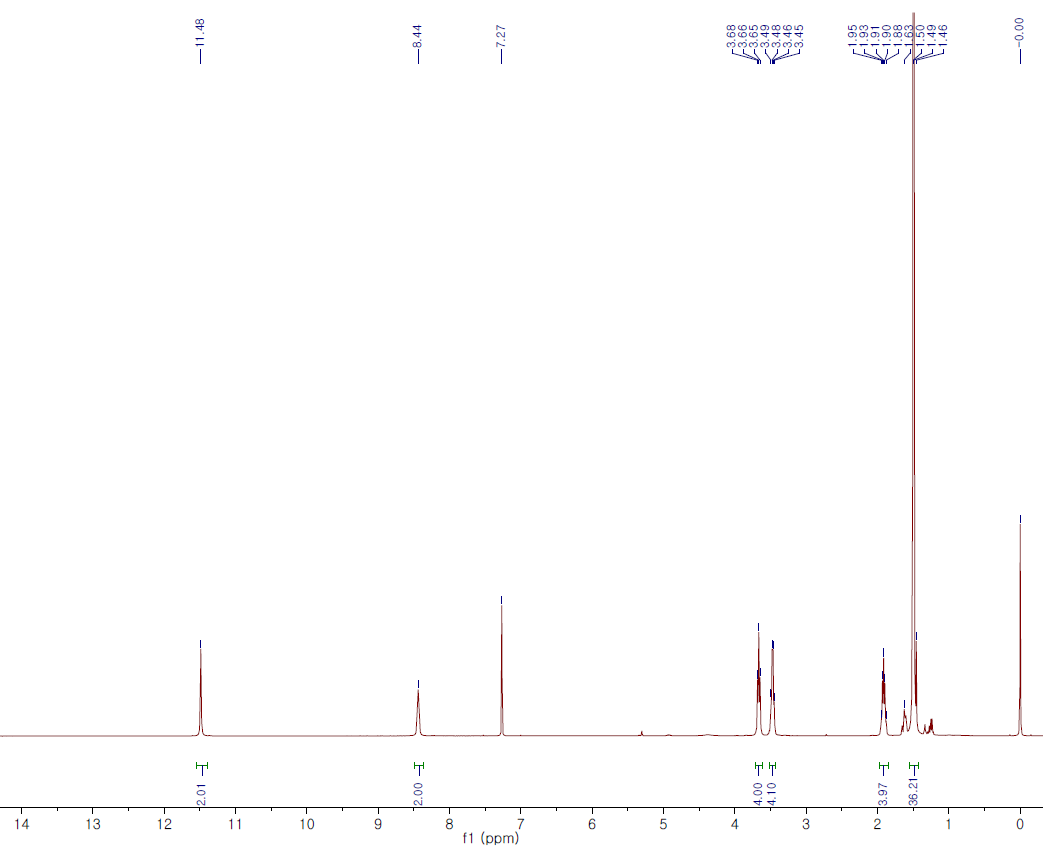


^1^H NMR spectrum of **5** (CDCl_3_, 400 MHz)


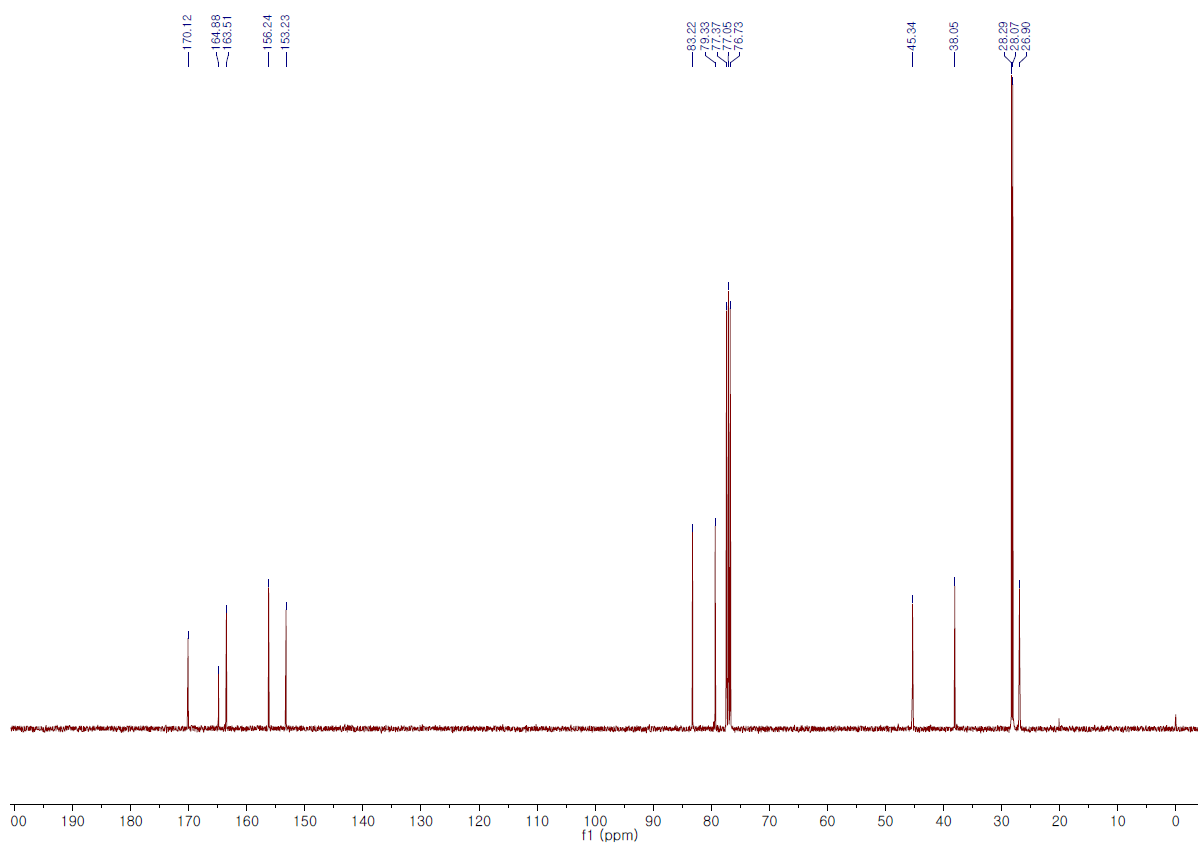


^13^C NMR spectrum of **5** (CDCl_3_, 101 MHz)


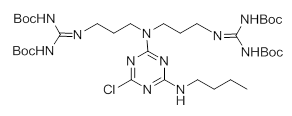

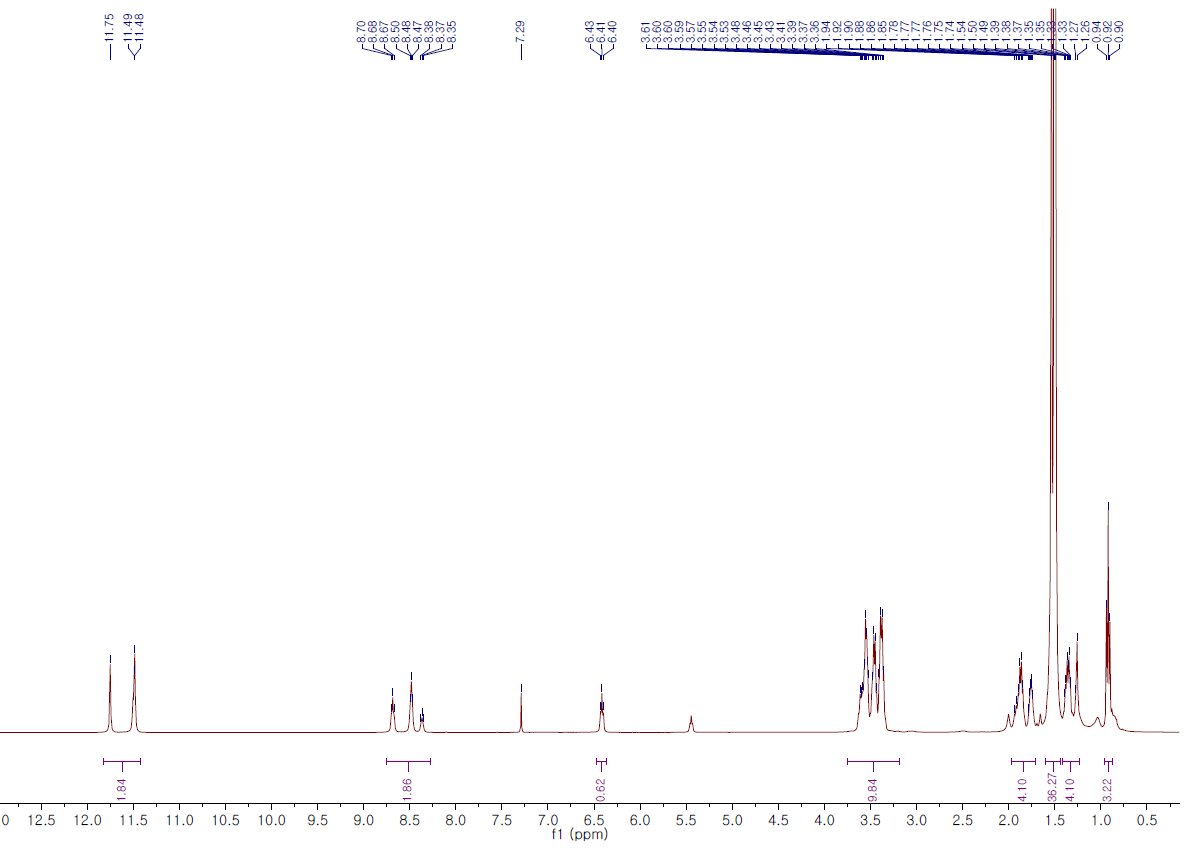


^1^H NMR spectrum of **6a** (CDCl_3_, 400 MHz)


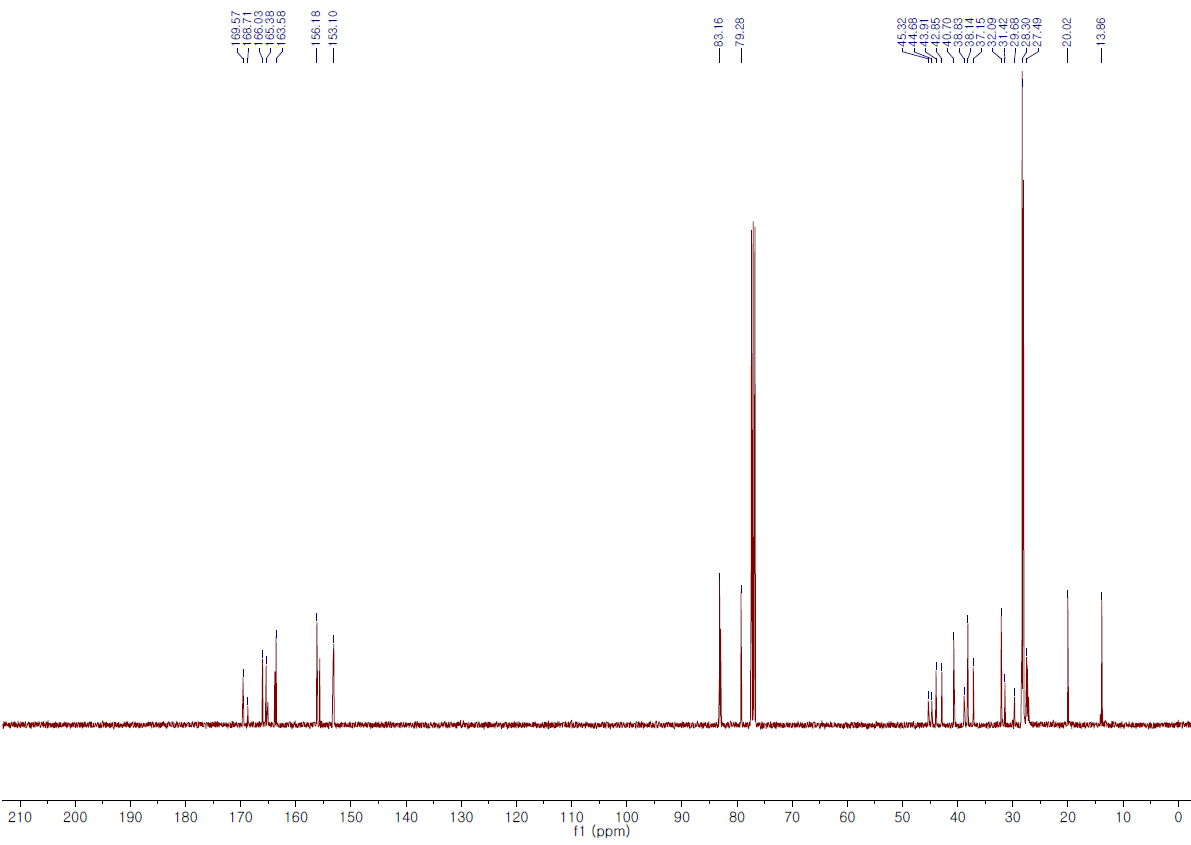


^1^H NMR spectrum of **6a** (CDCl_3_, 101 MHz)


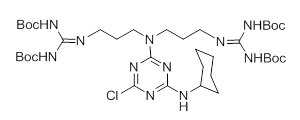

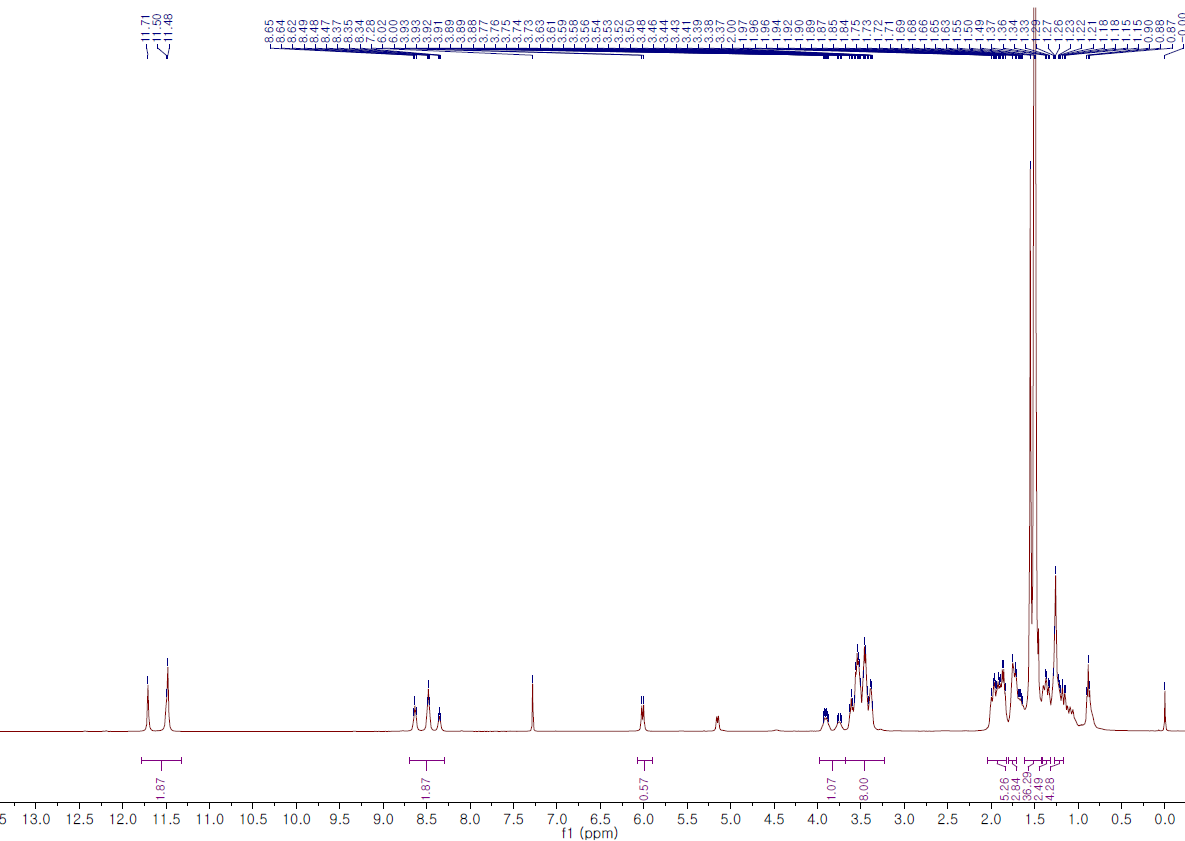


^1^H NMR spectrum of **6b** (CDCl_3_, 400 MHz)


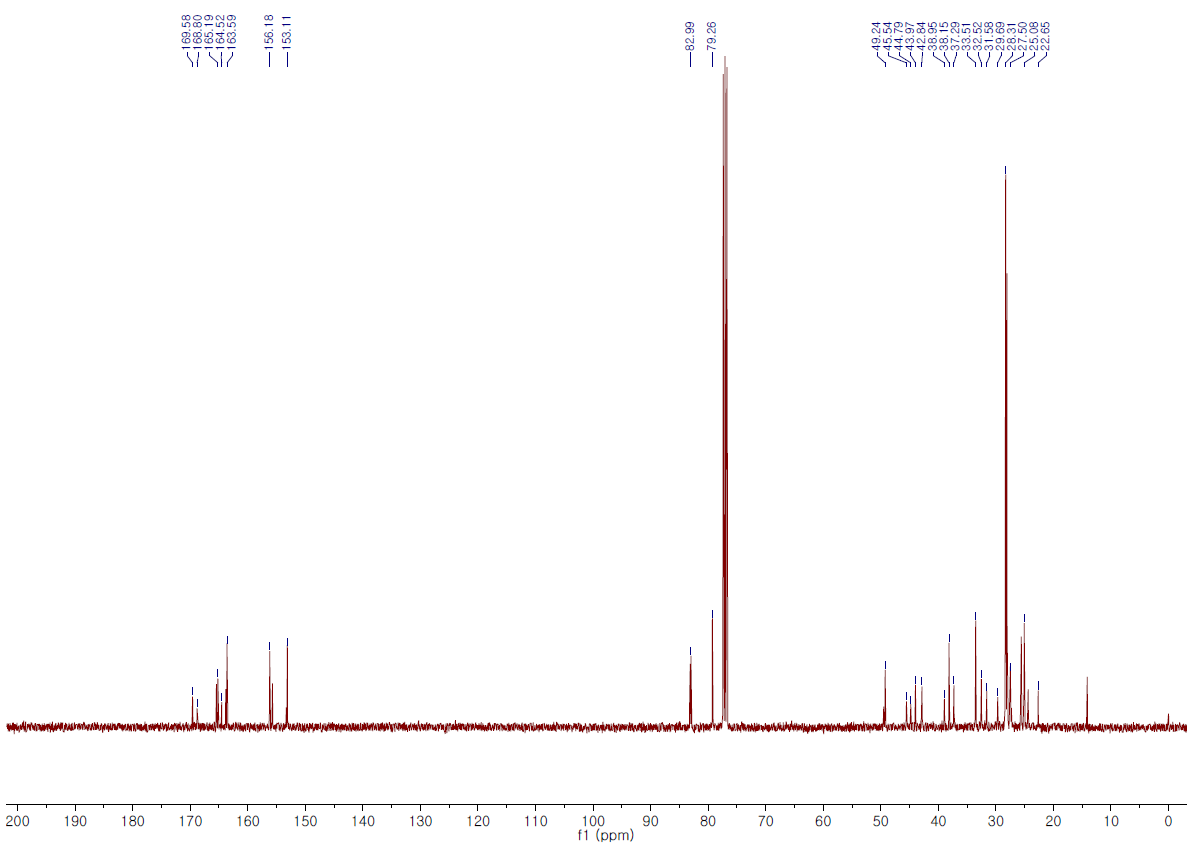


^1^H NMR spectrum of **6b** (CDCl_3_, 101 MHz)


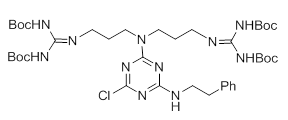

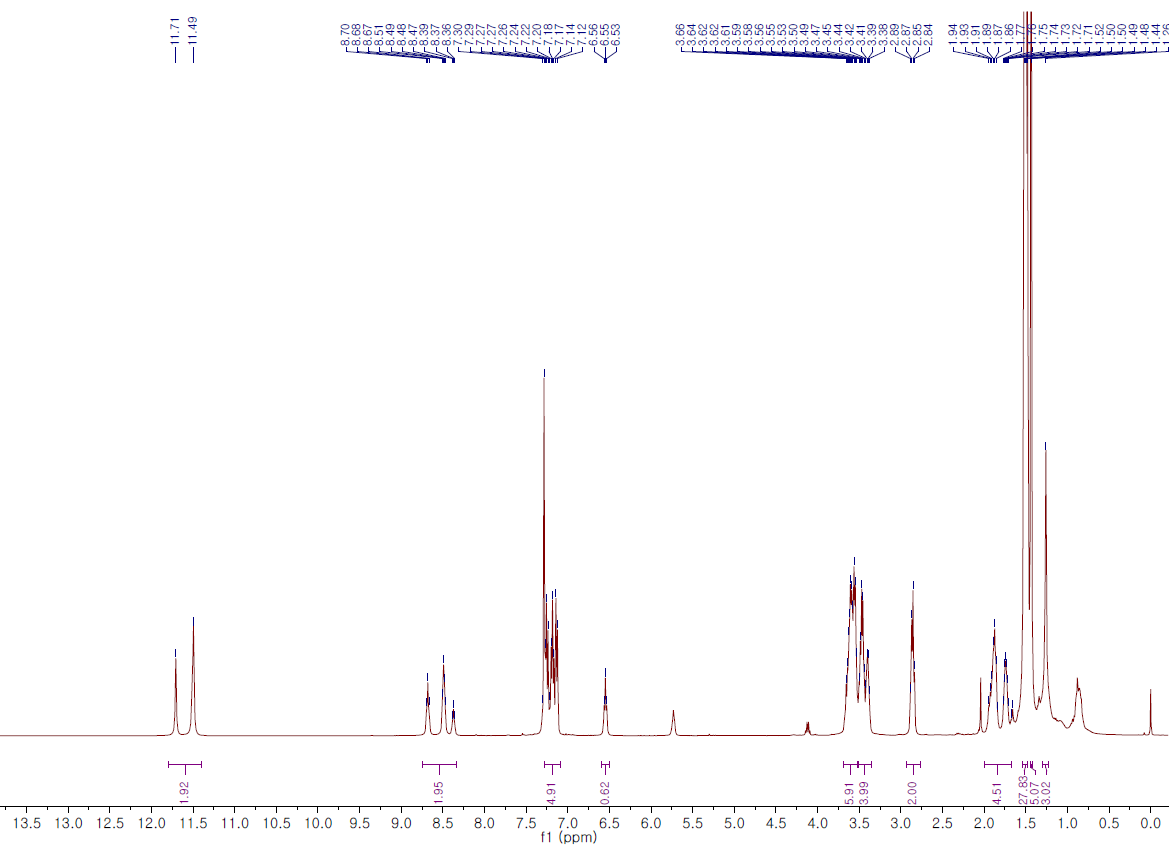


^1^H NMR spectrum of **6c** (CDCl_3_, 400 MHz)
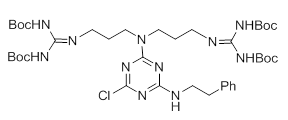

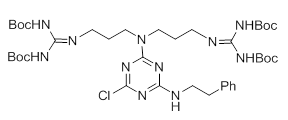

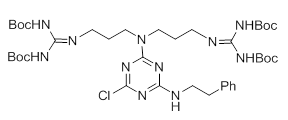

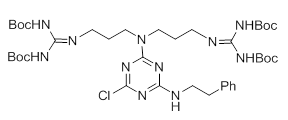

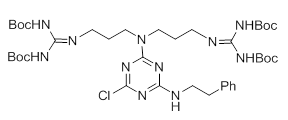


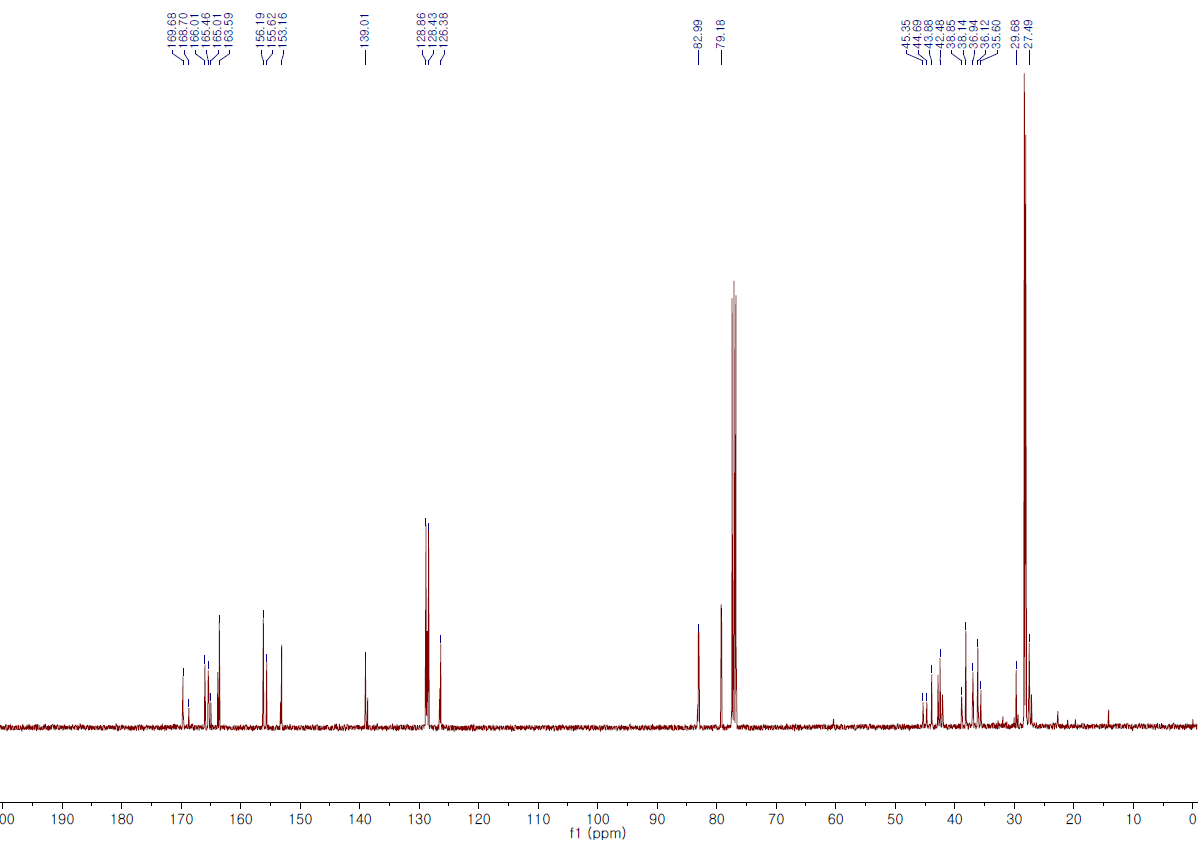


^1^H NMR spectrum of **6c** (CDCl_3_, 101 MHz)


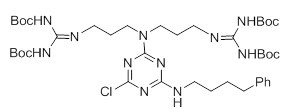

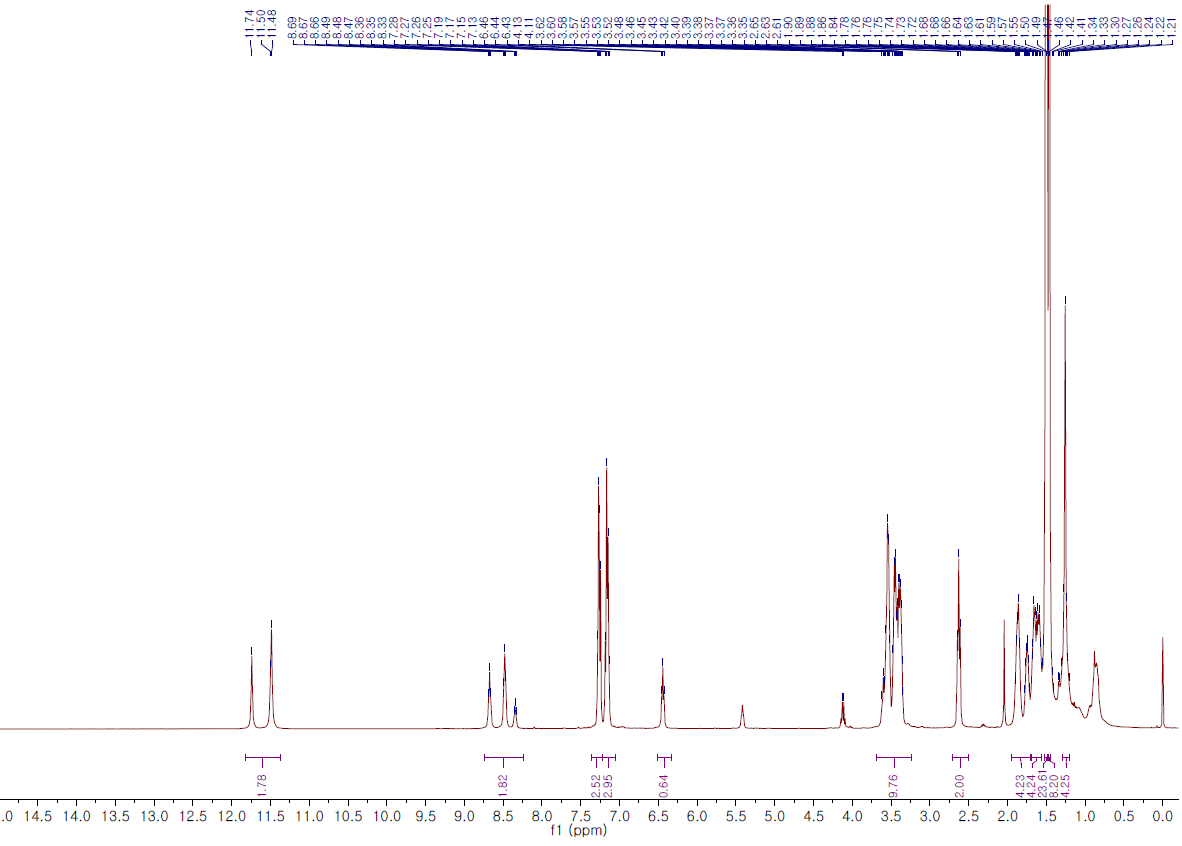


^1^H NMR spectrum of **6d** (CDCl_3_, 400 MHz)


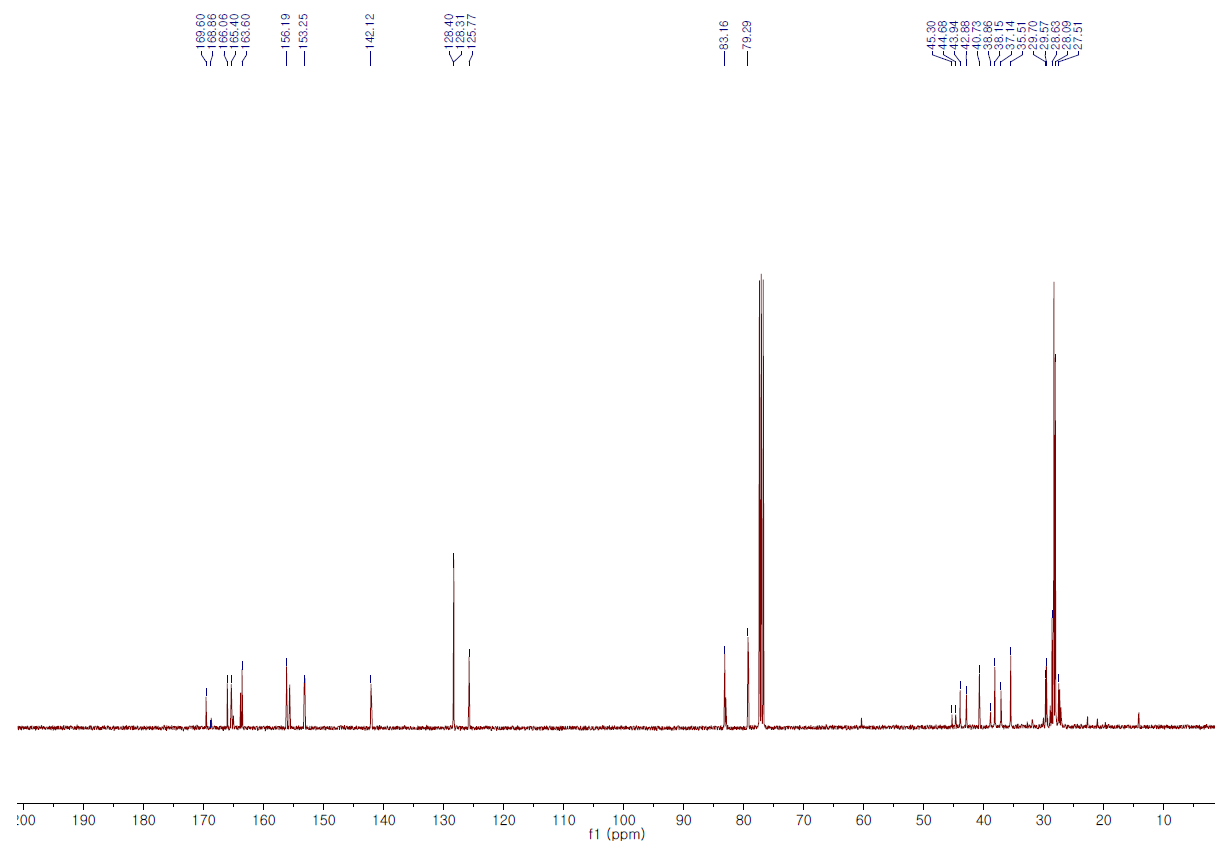


^1^H NMR spectrum of **6d** (CDCl_3_, 101 MHz)


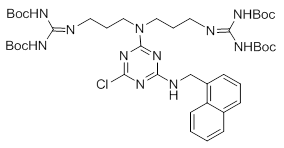

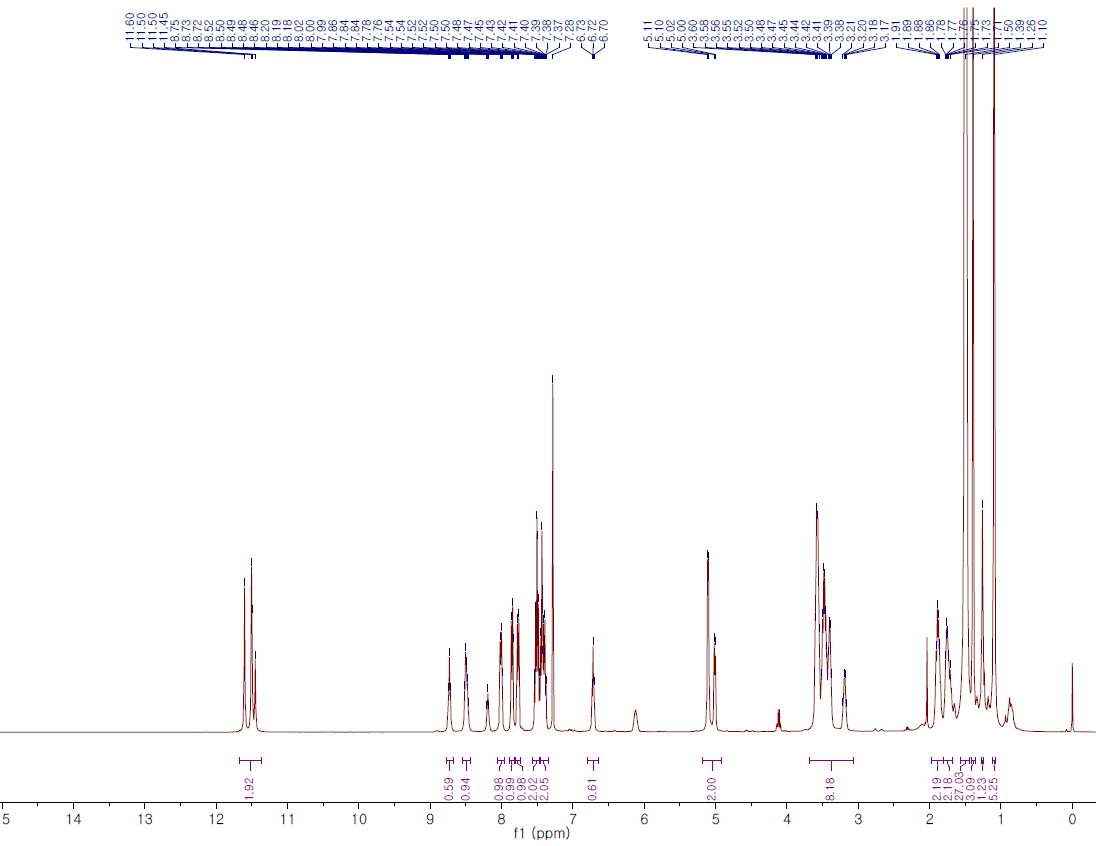


^1^H NMR spectrum of **6e** (CDCl_3_, 400 MHz)
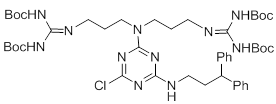


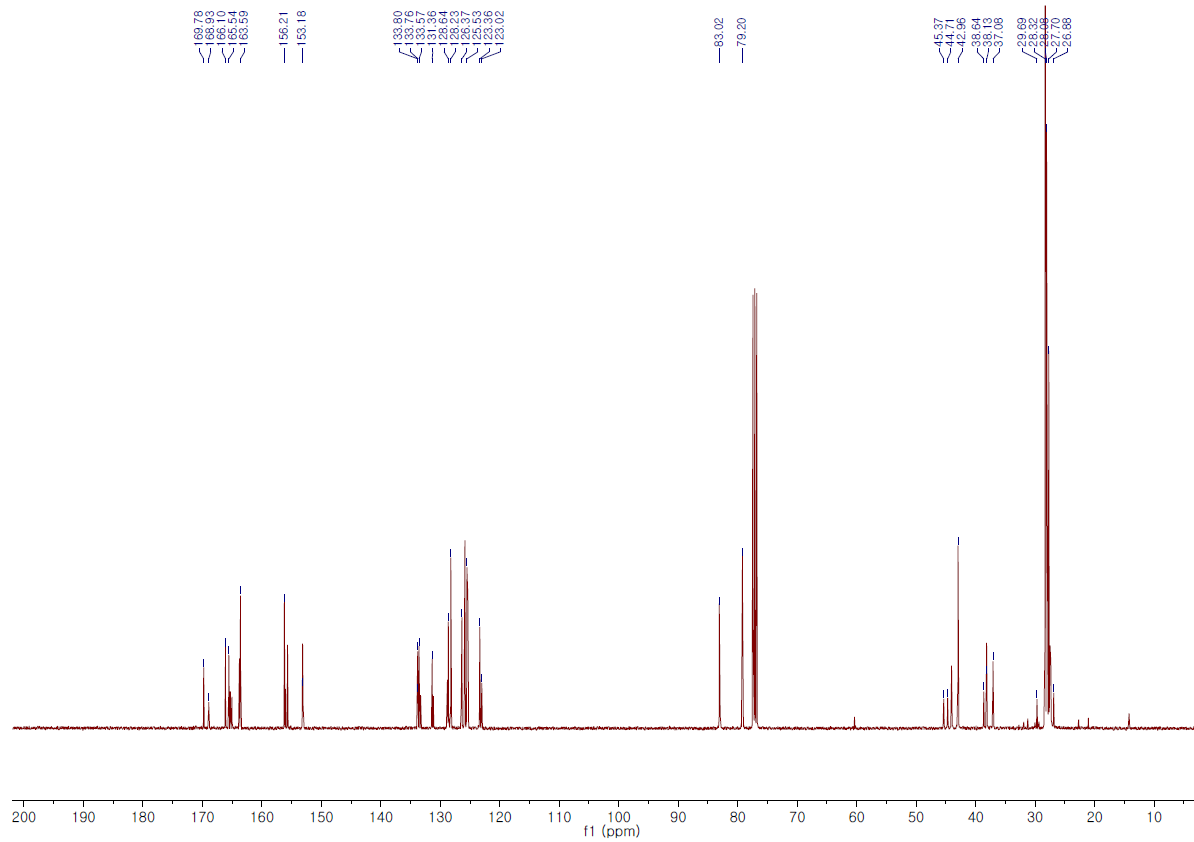


^1^H NMR spectrum of **6e** (CDCl_3_, 101 MHz)


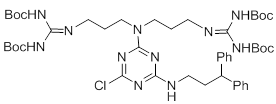

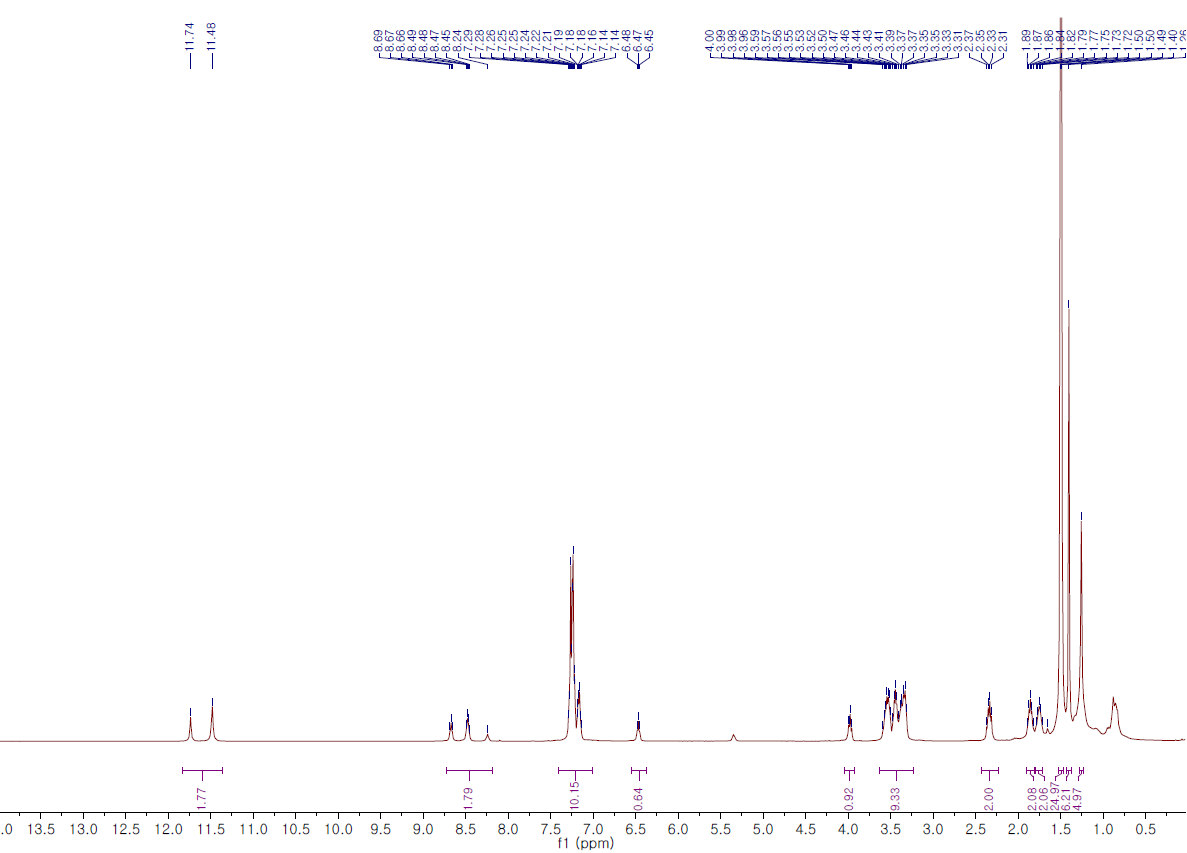


^1^H NMR spectrum of **6f** (CDCl_3_, 400 MHz)


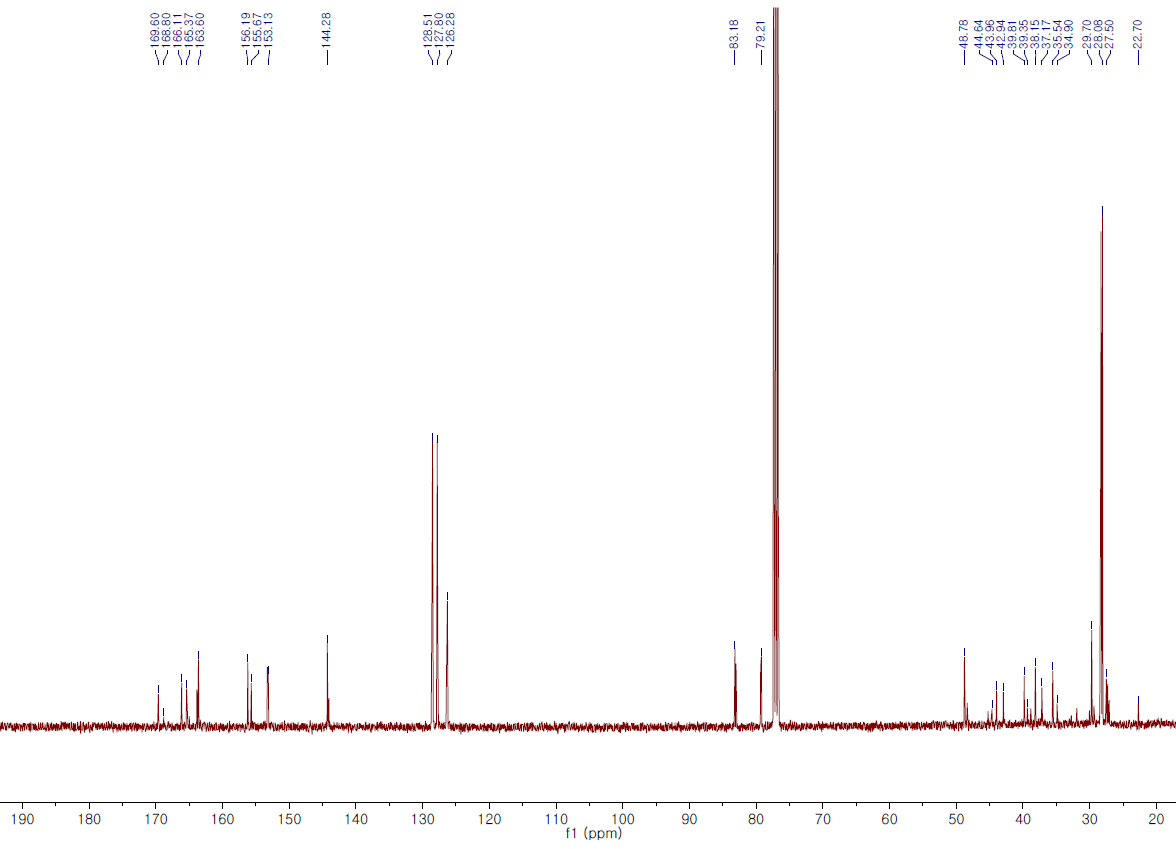


^1^H NMR spectrum of **6f** (CDCl_3_, 101 MHz)


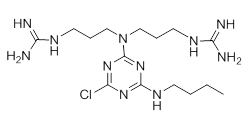

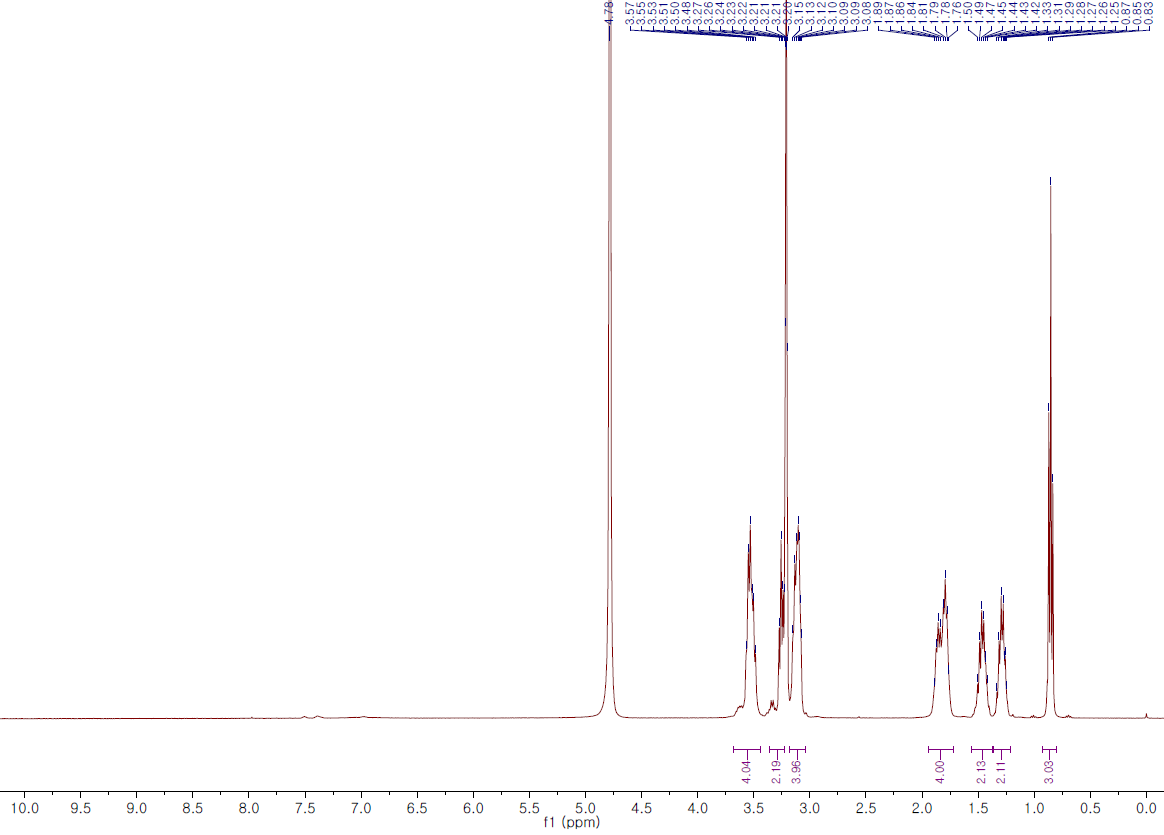


^1^H NMR spectrum of **MG-1** (MeOD, 400 MHz)


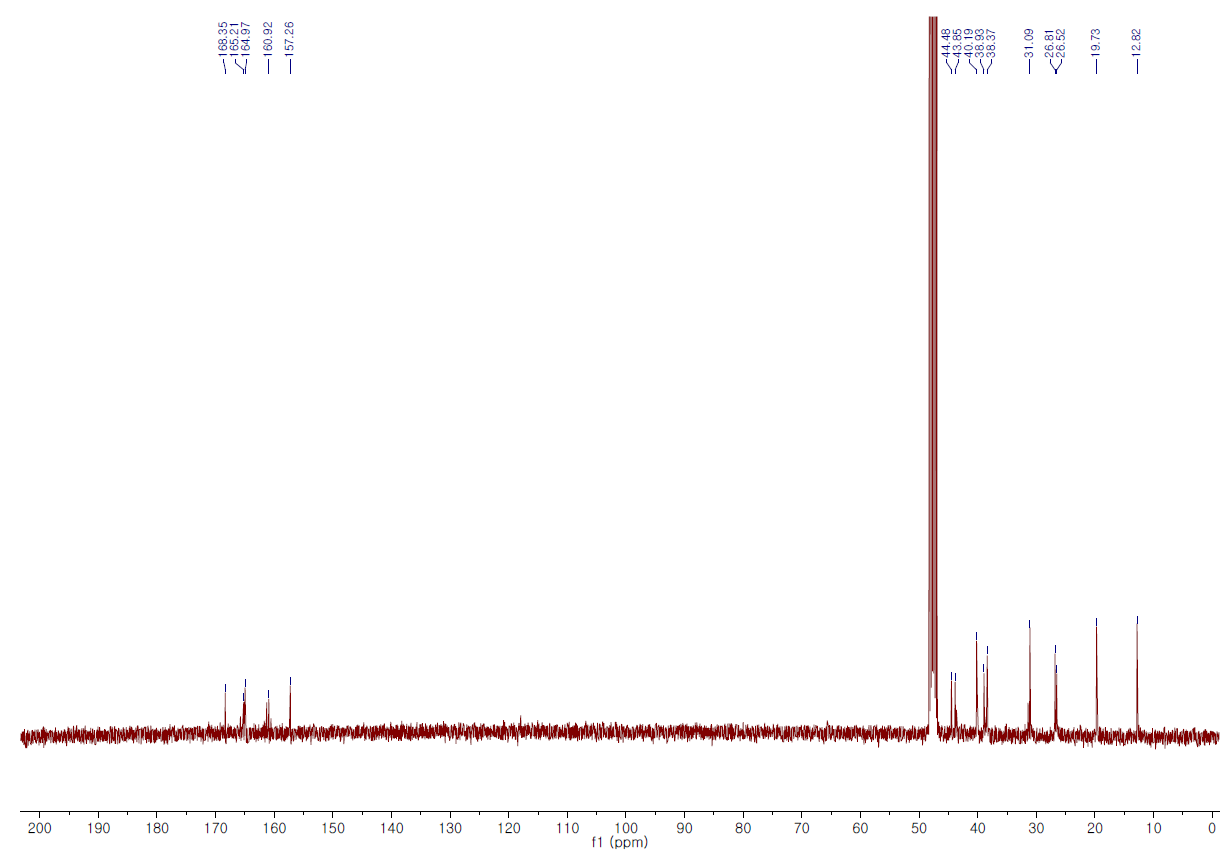


^13^C NMR spectrum of **MG-1** (MeOD, 101 MHz)


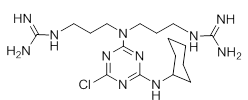

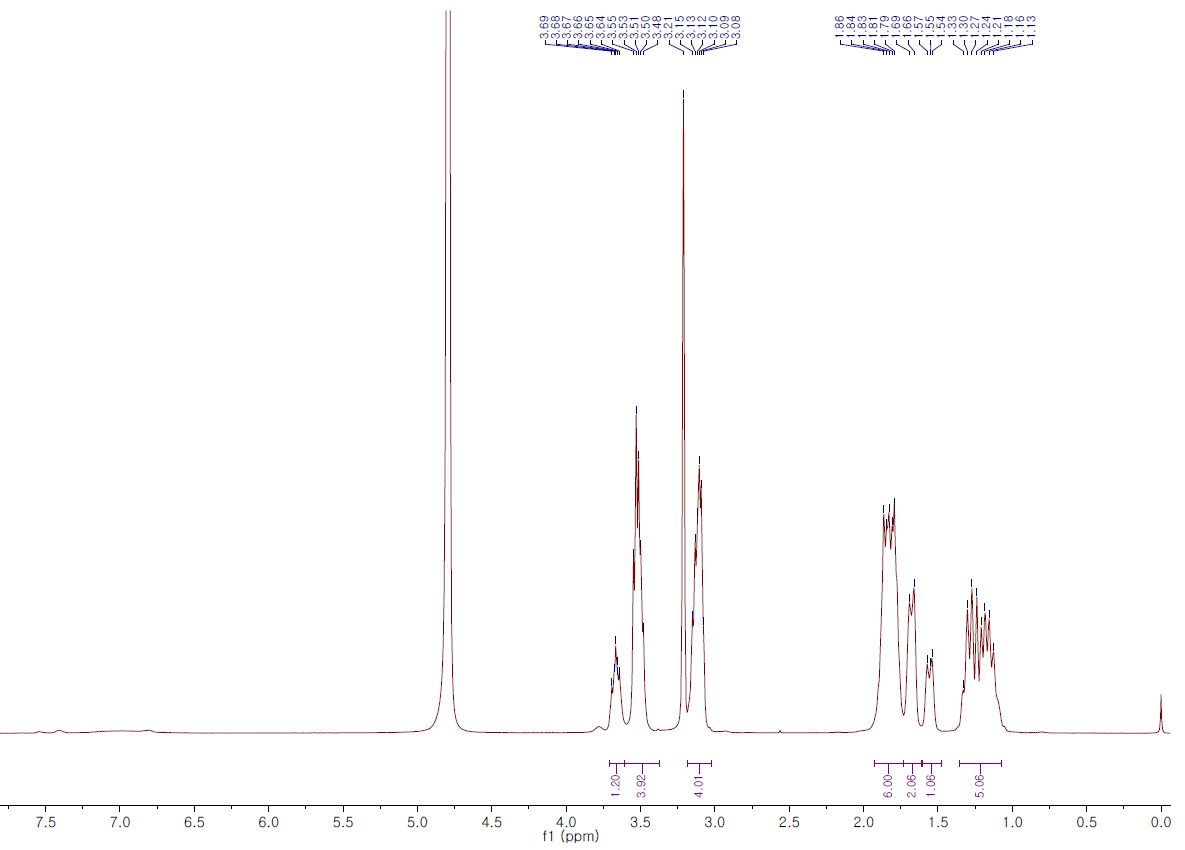


^1^H NMR spectrum of **MG-2** (MeOD, 400 MHz)


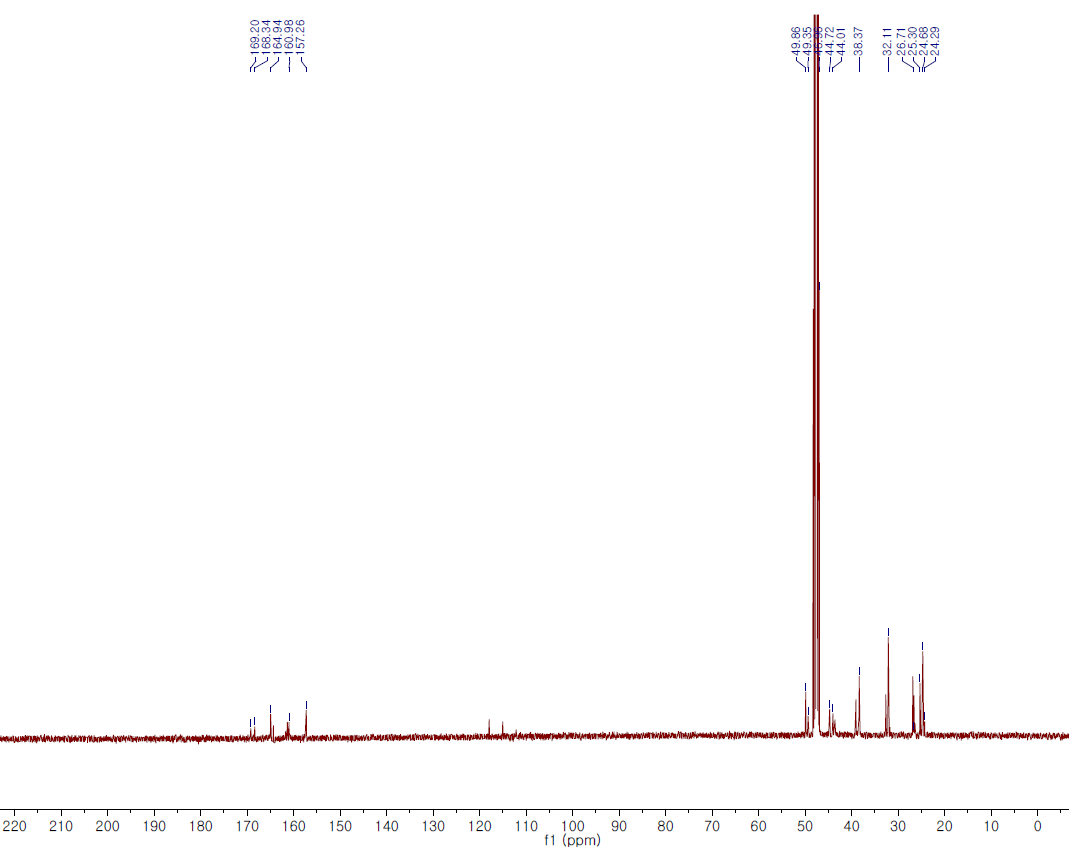


^13^C NMR spectrum of **MG-2** (MeOD, 101 MHz)


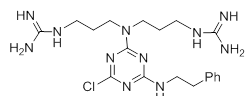

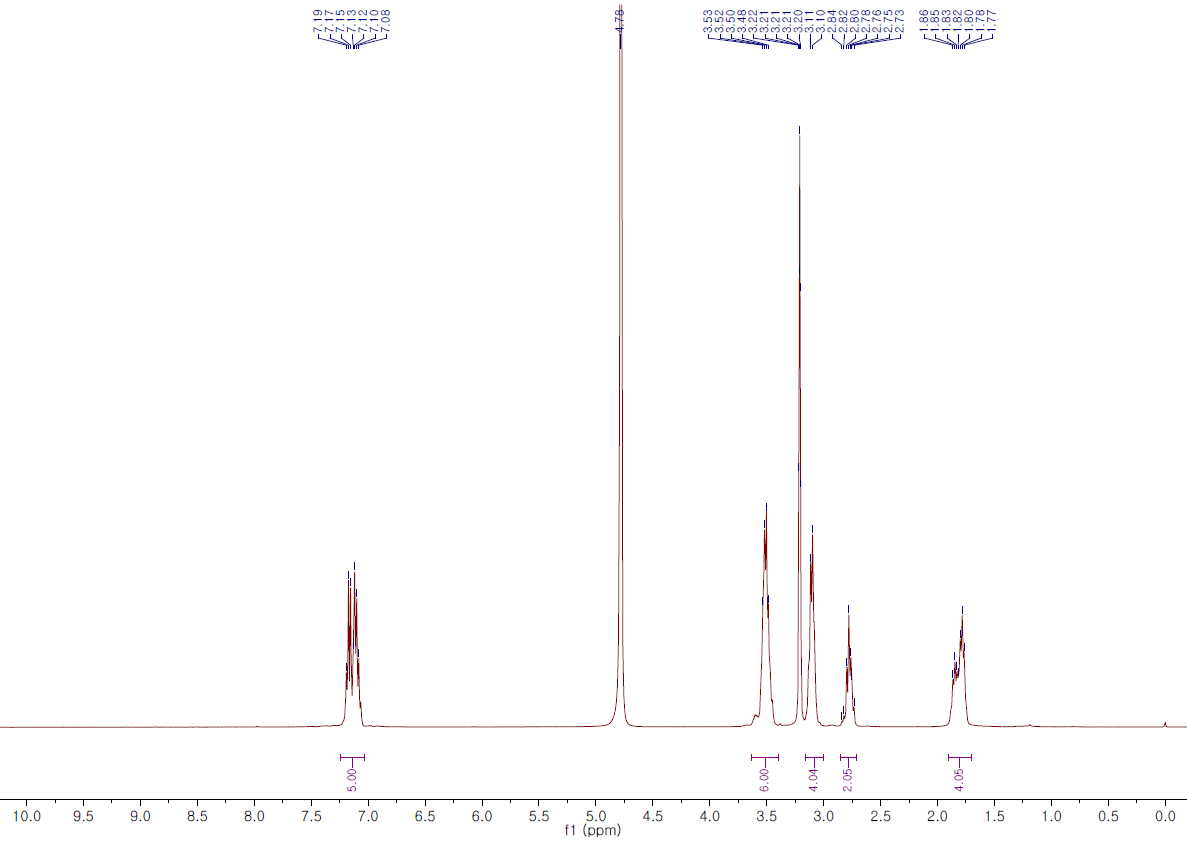


^1^H NMR spectrum of **MG-3** (MeOD, 400 MHz)


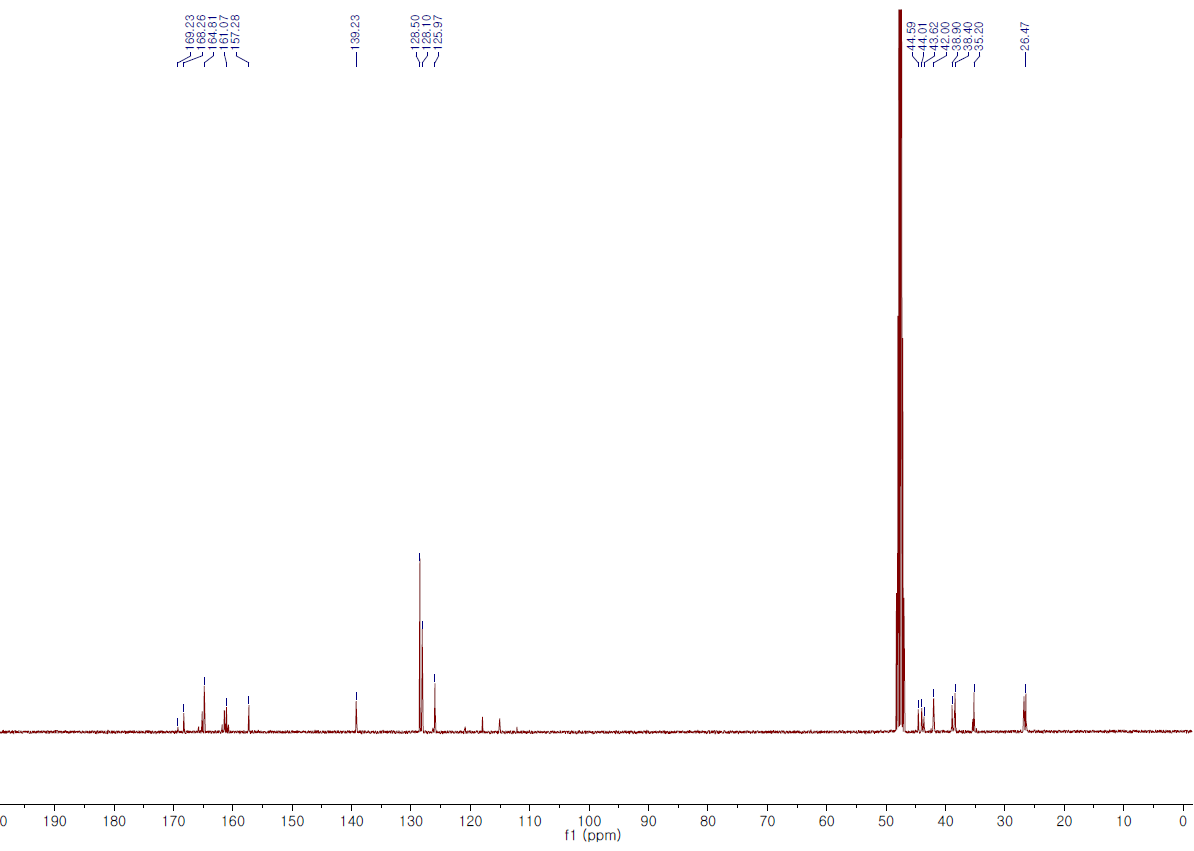


^13^C NMR spectrum of **MG-3** (MeOD, 101 MHz)


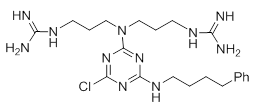

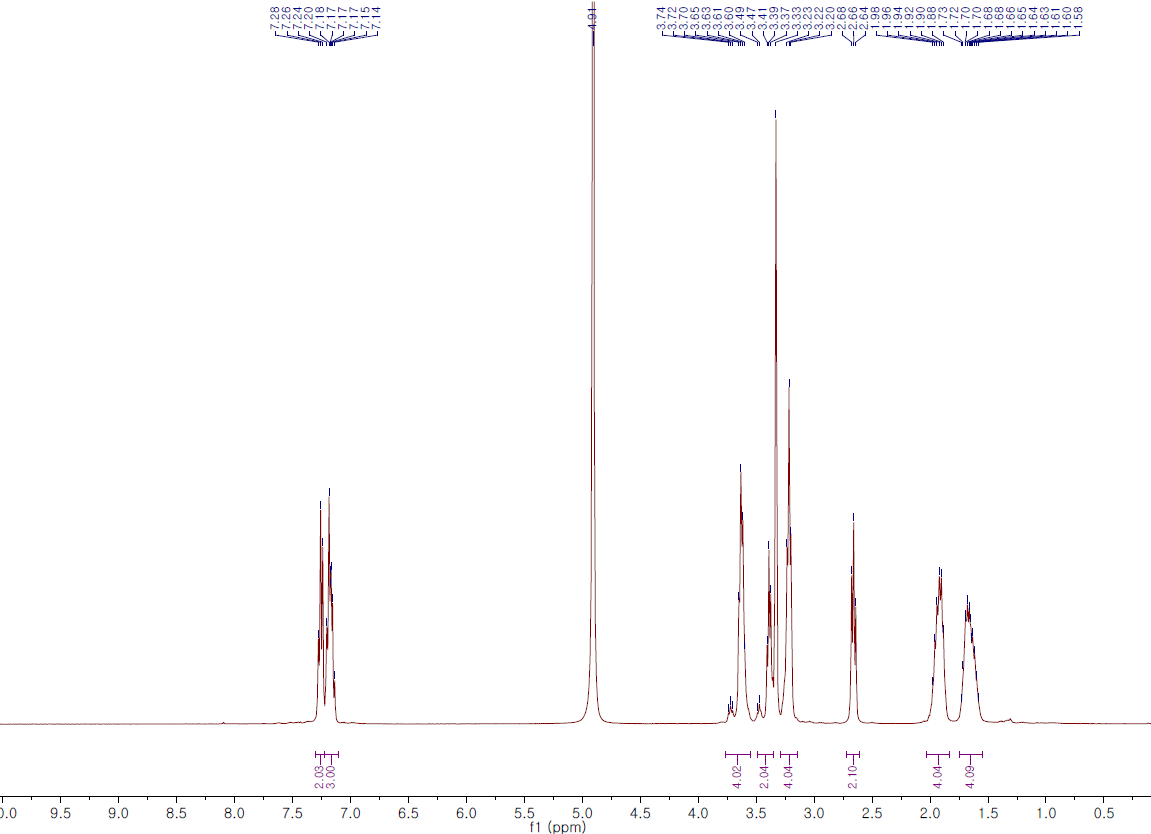


^1^H NMR spectrum of **MG-4** (MeOD, 400 MHz)


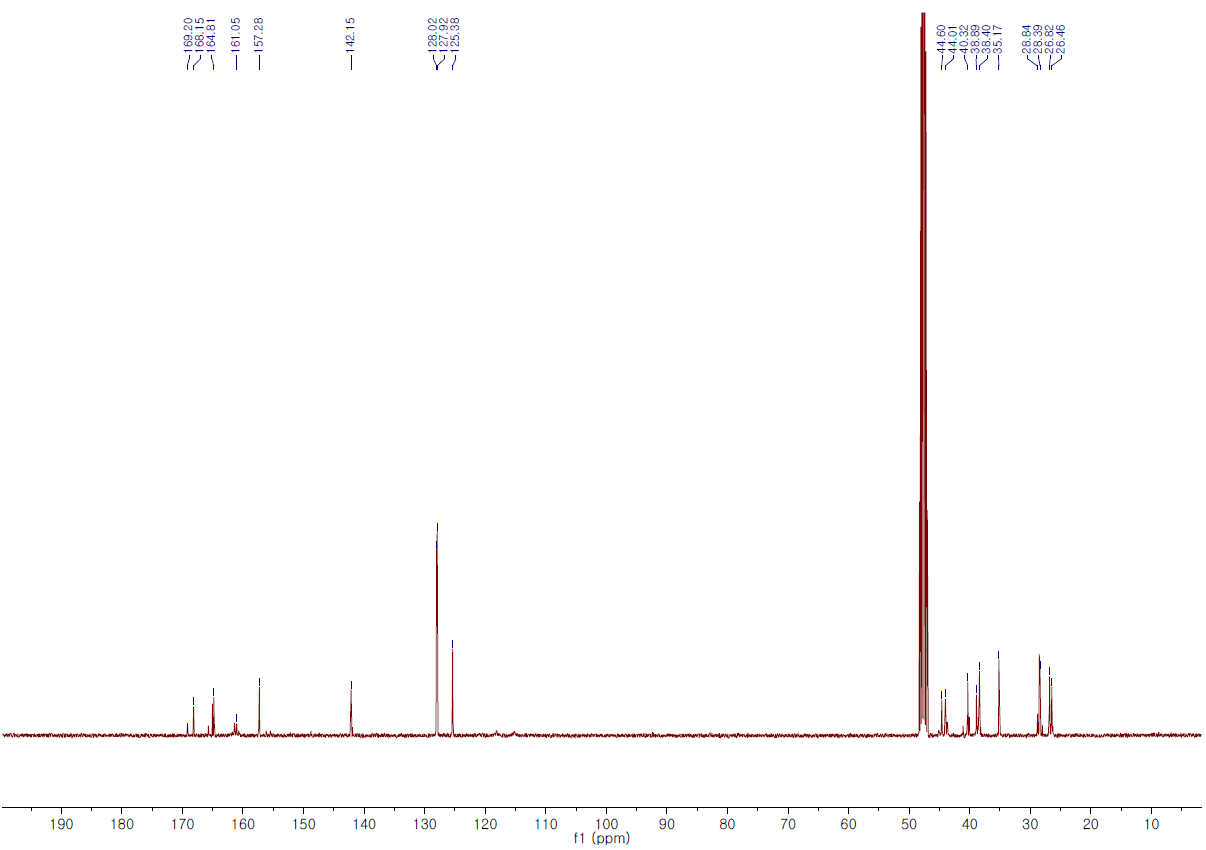


^13^C NMR spectrum of **MG-4** (MeOD, 101 MHz)


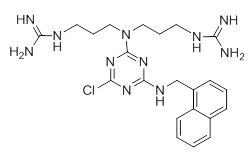

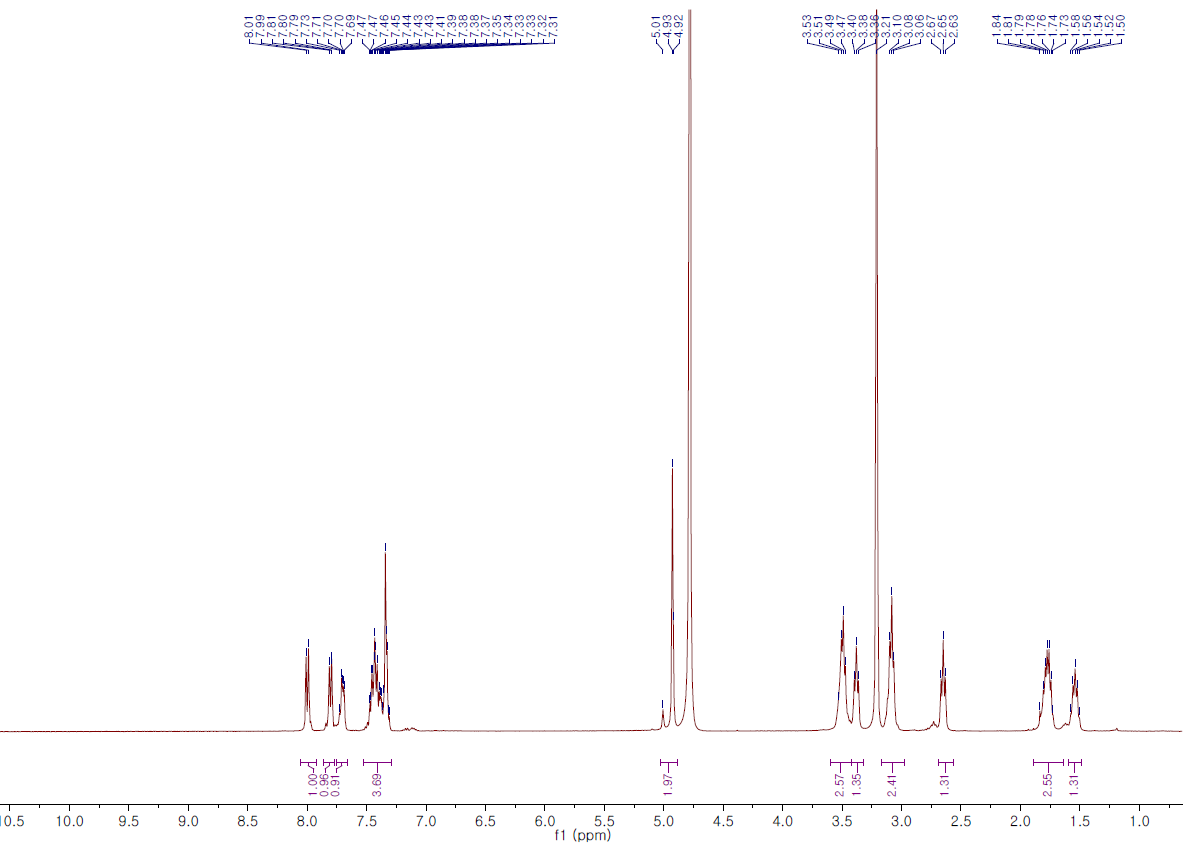


^1^H NMR spectrum of **MG-5** (MeOD, 400 MHz)


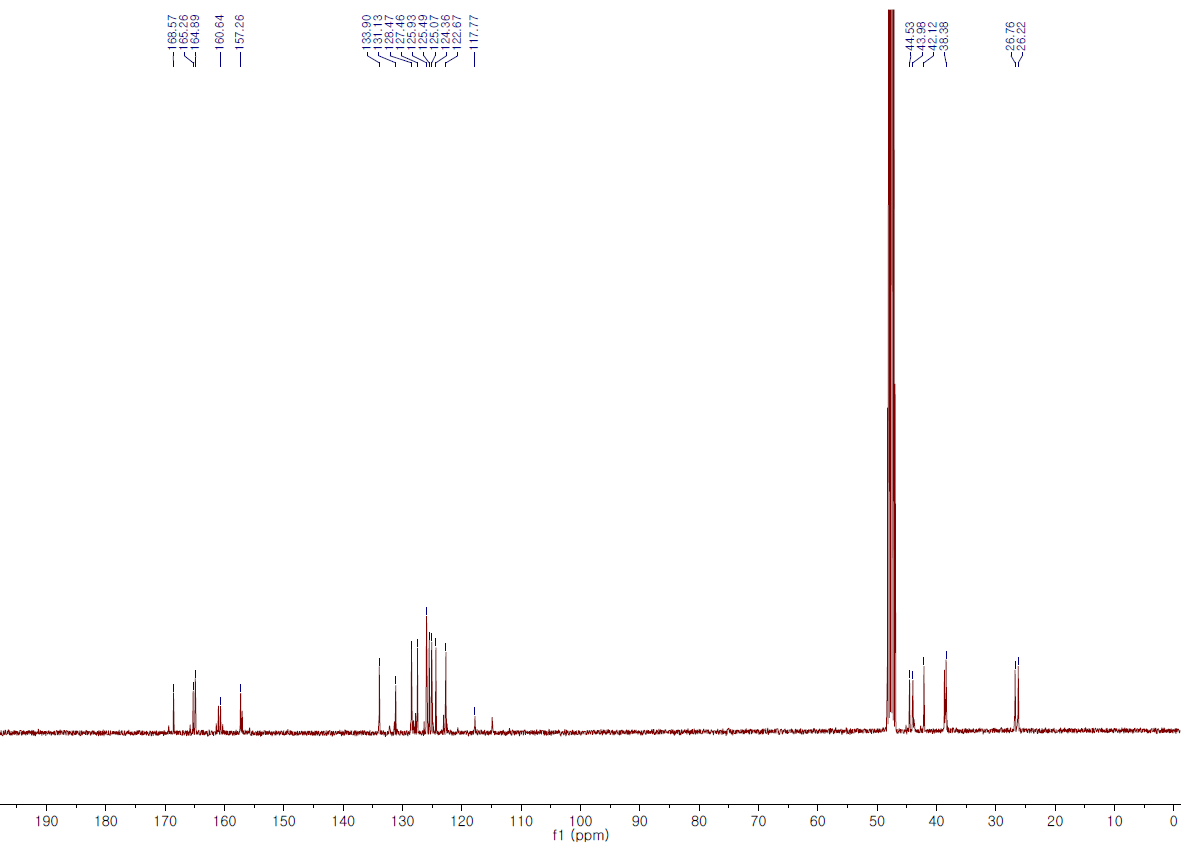


^13^C NMR spectrum of **MG-5** (MeOD, 101 MHz)


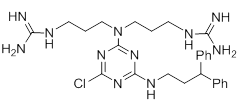

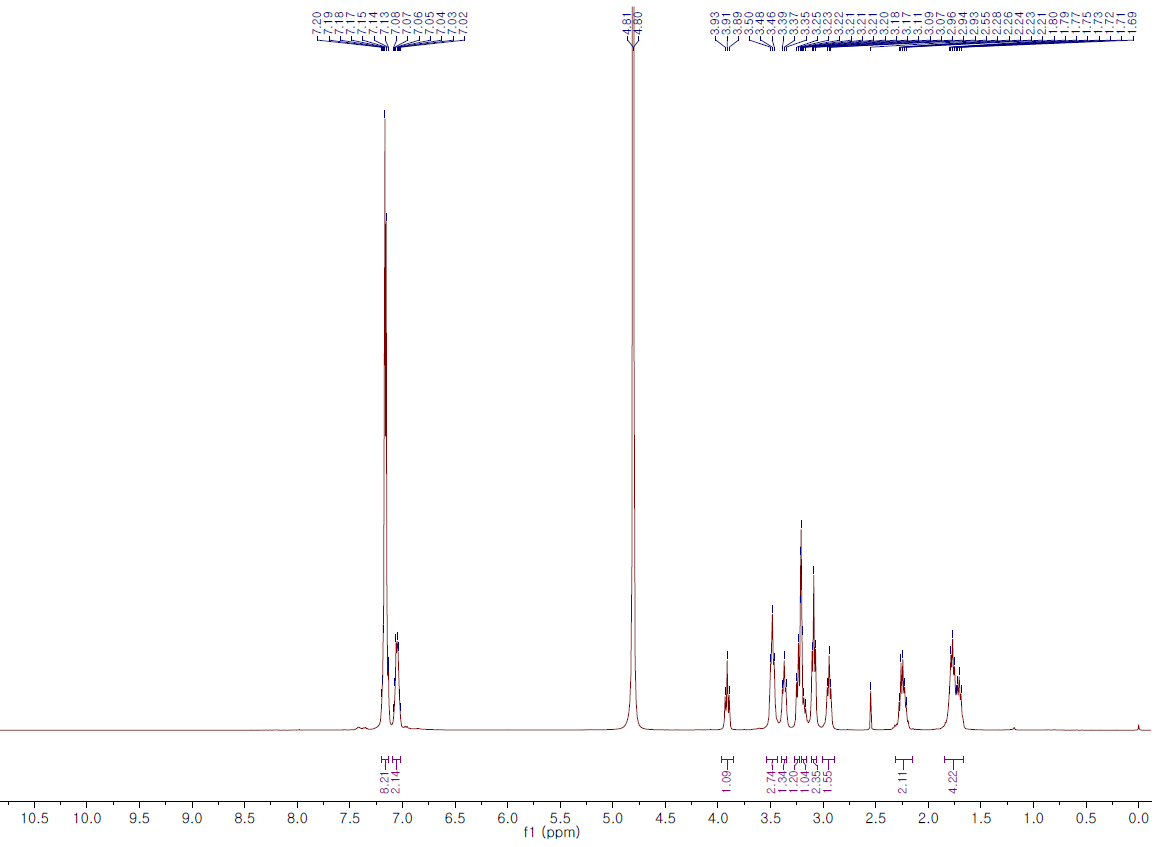


^1^H NMR spectrum of **MG-6** (MeOD, 400 MHz)


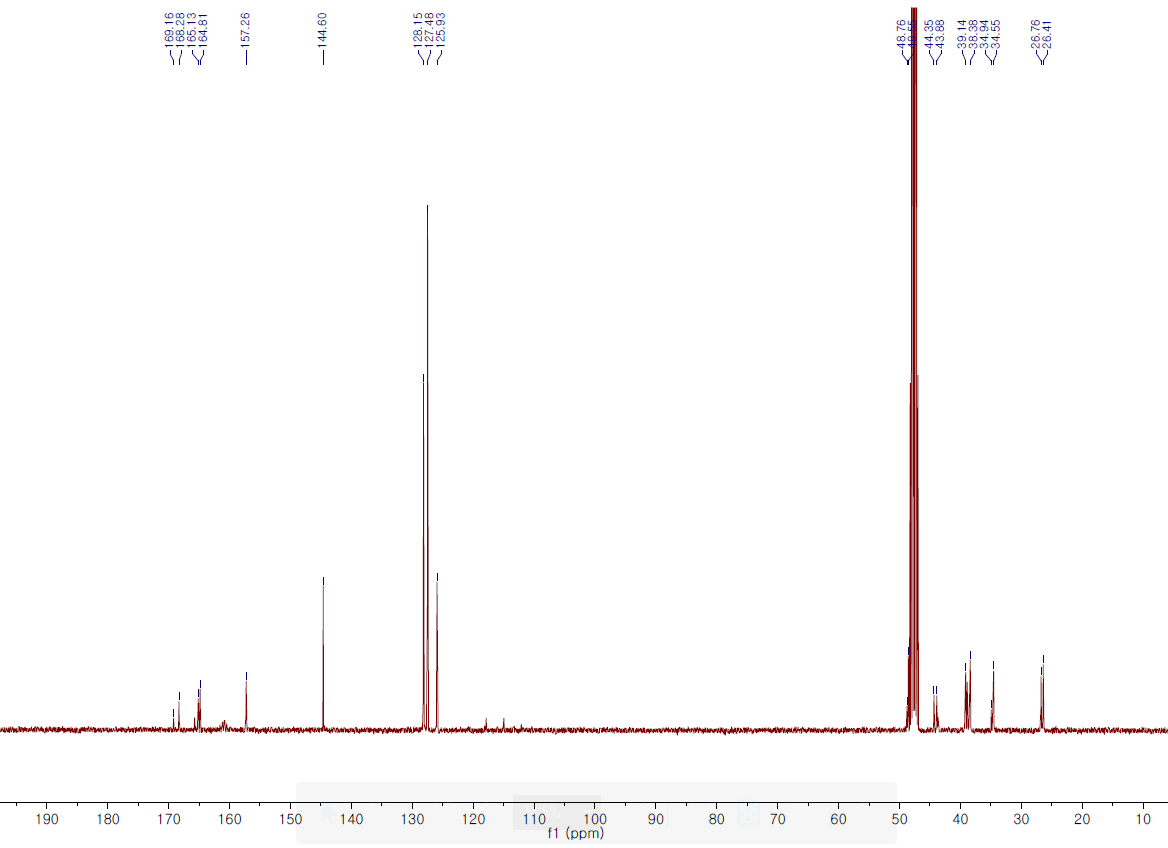


^13^C NMR spectrum of **MG-6** (MeOD, 101 MHz)


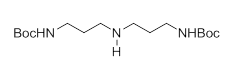

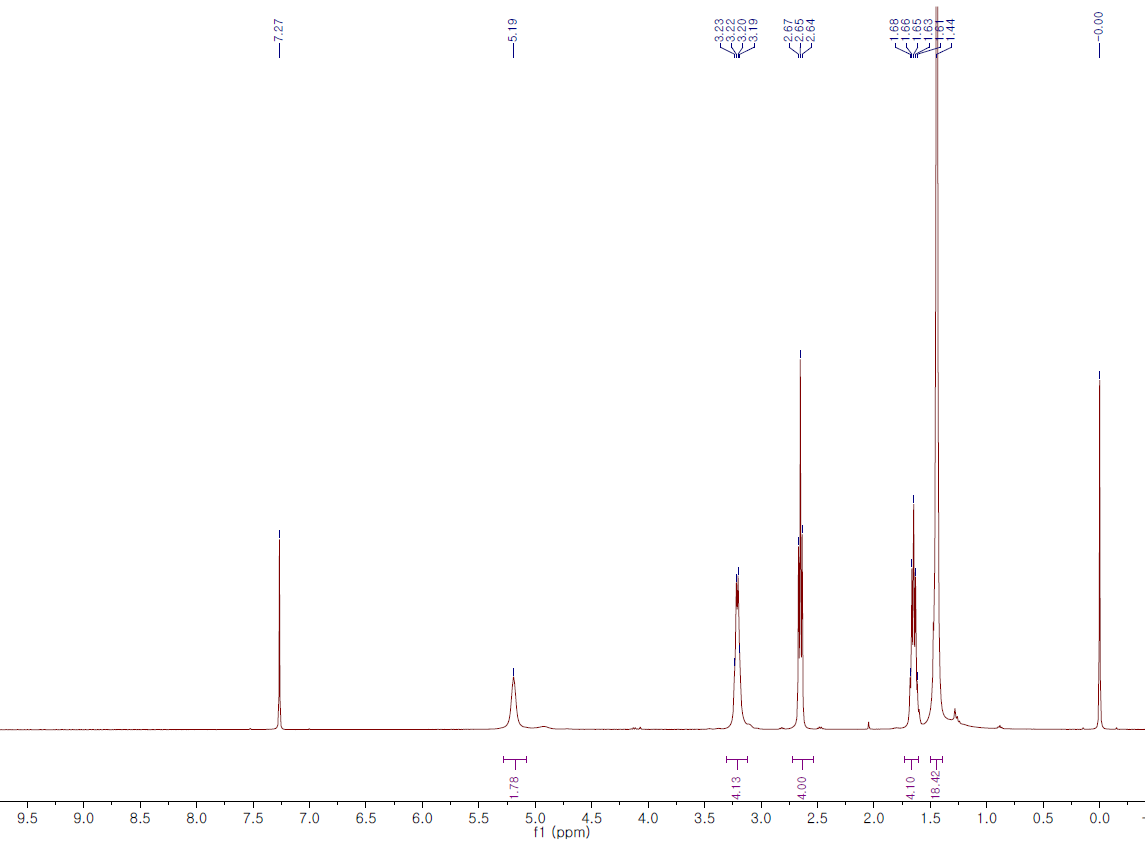


^1^H NMR spectrum of **7** (CDCl_3_, 400 MHz)


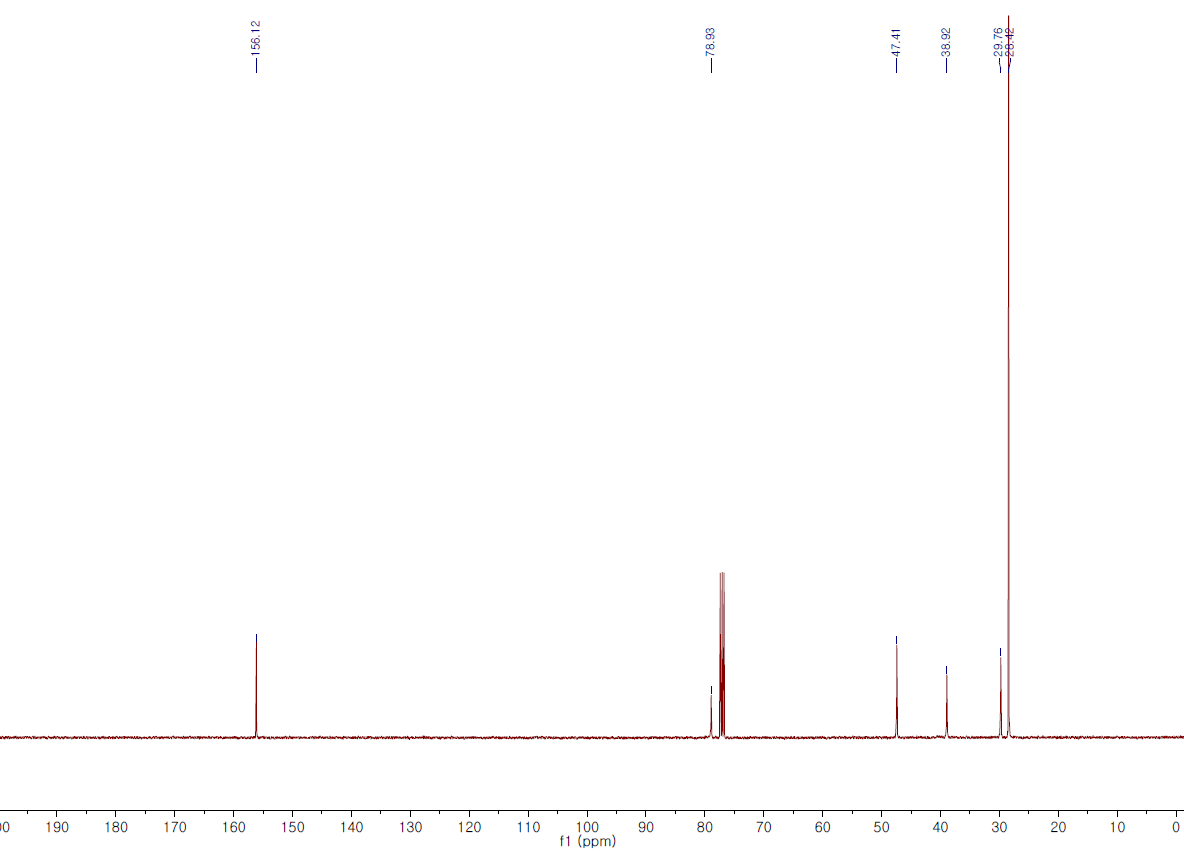


^13^C NMR spectrum of **7** (CDCl_3_, 101 MHz)


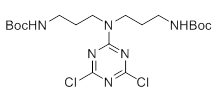

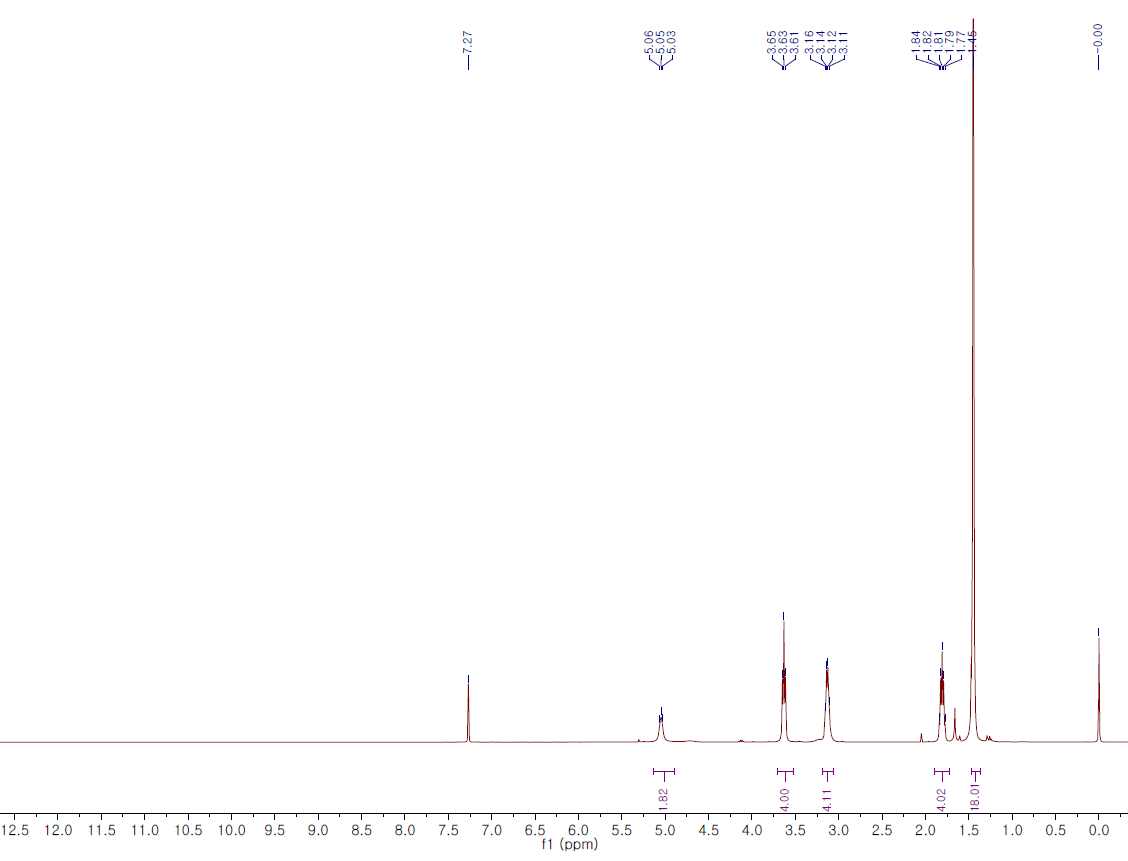


^1^H NMR spectrum of **8** (CDCl_3_, 400 MHz)


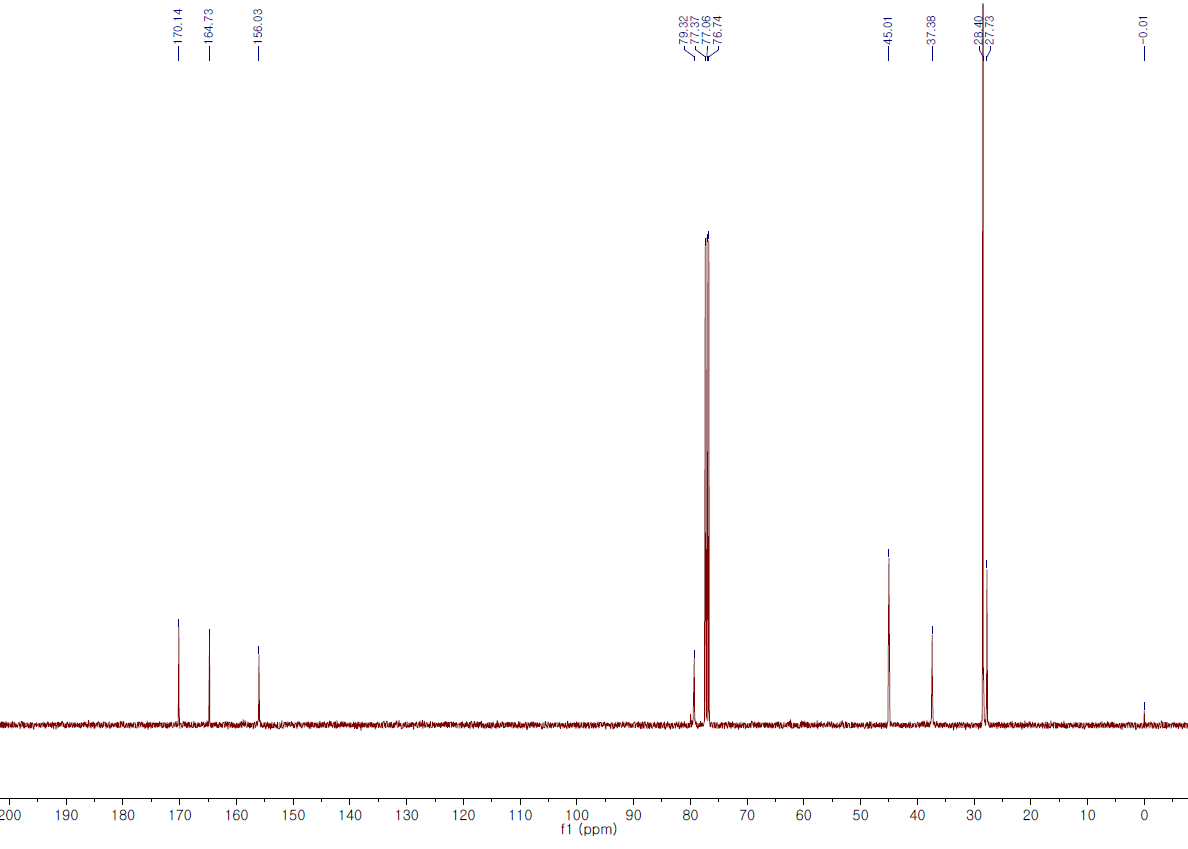


^13^C NMR spectrum of **8** (CDCl_3_, 101 MHz)


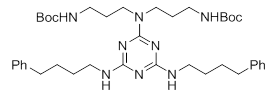

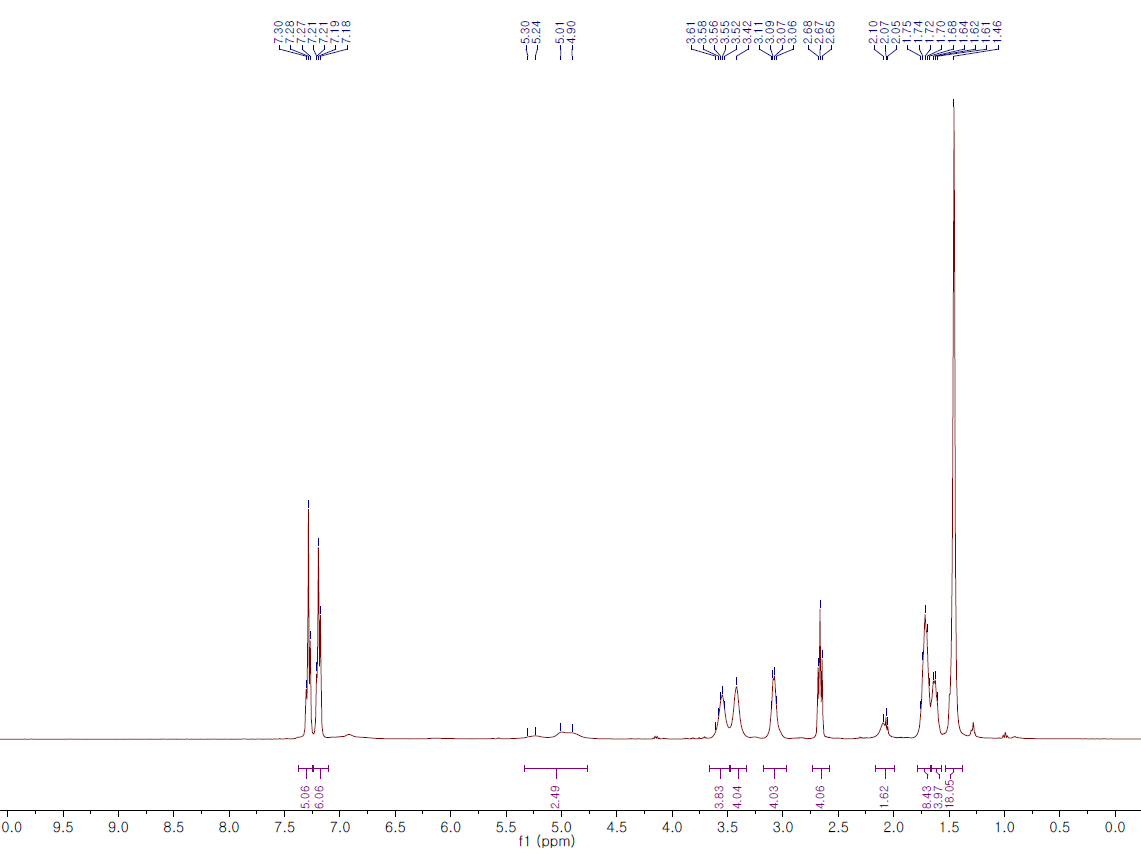


^1^H NMR spectrum of **9a** (CDCl_3_, 400 MHz)


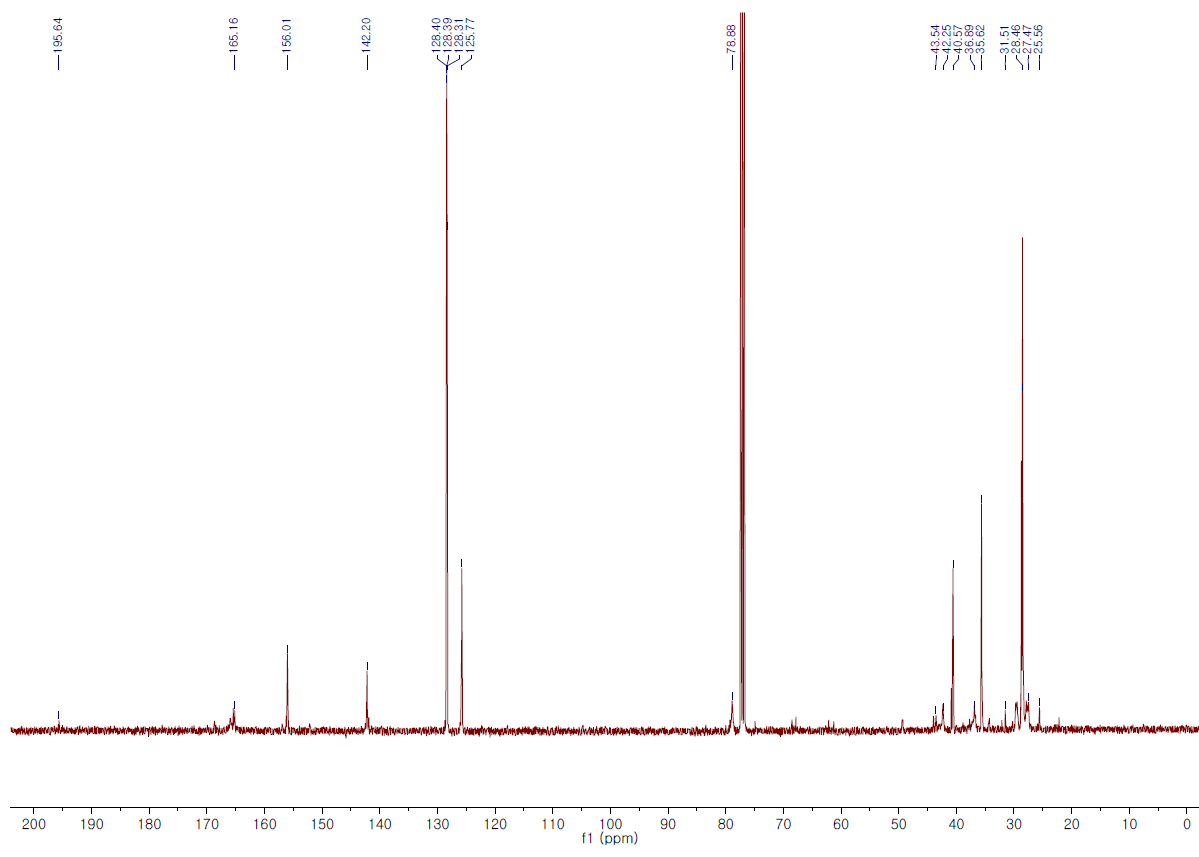


^13^C NMR spectrum of **9a** (CDCl_3_, 101 MHz)


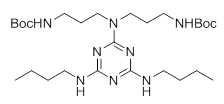

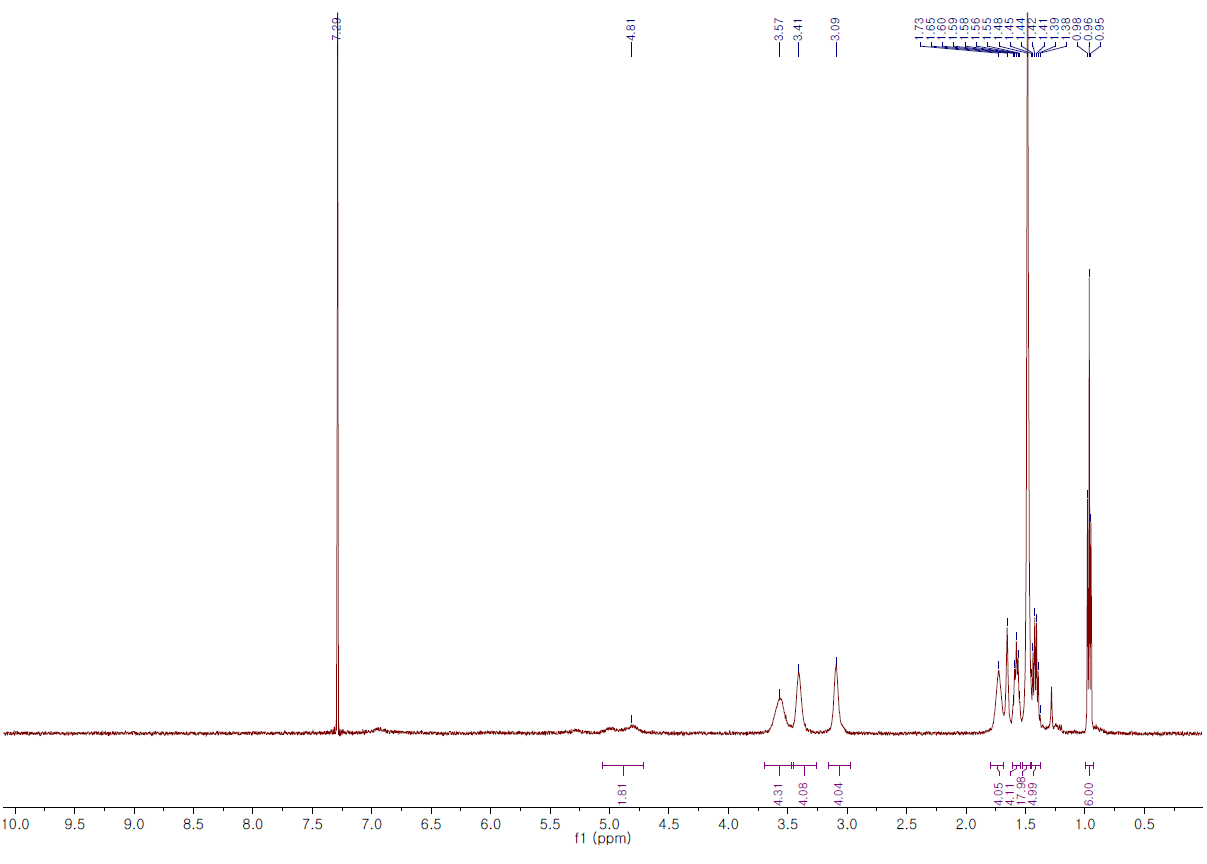


^1^H NMR spectrum of **9b** (CDCl_3_, 400 MHz)


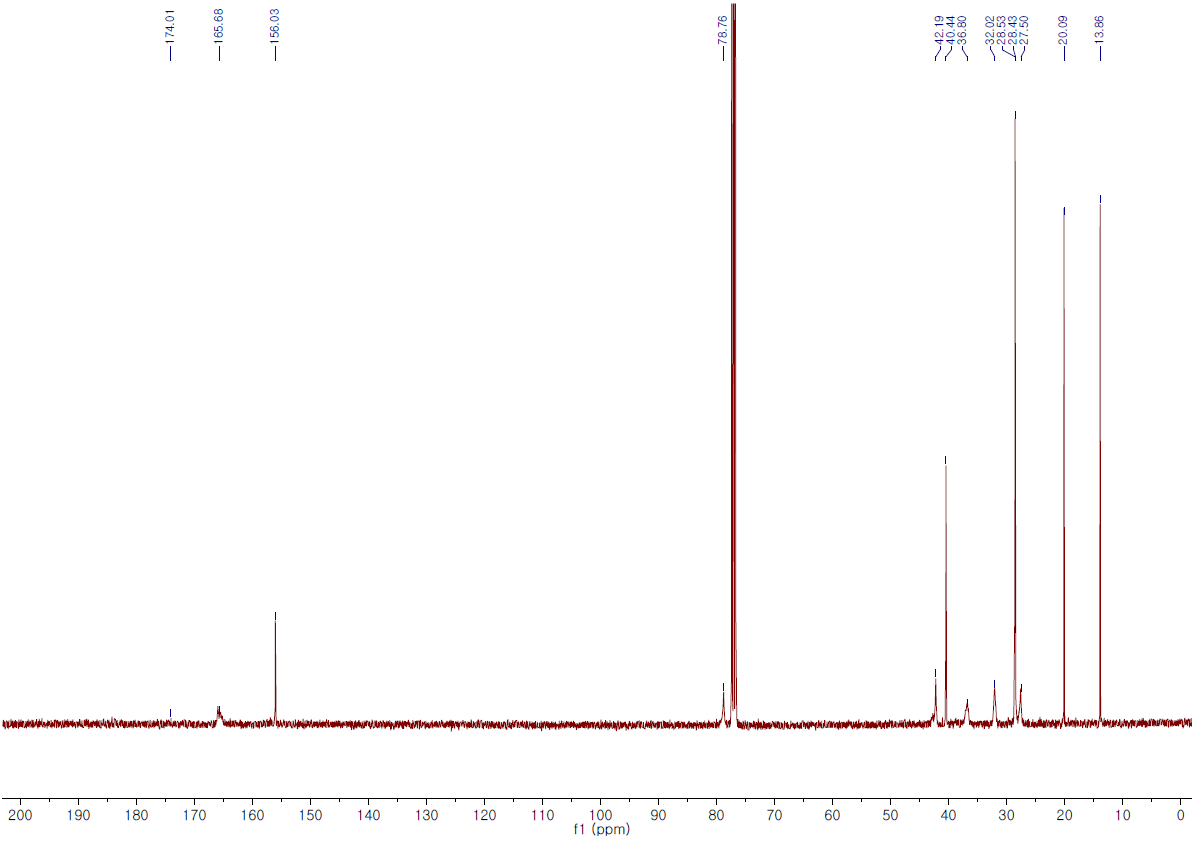


^13^C NMR spectrum of **9b** (CDCl_3_, 101 MHz)


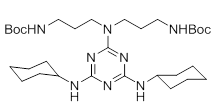

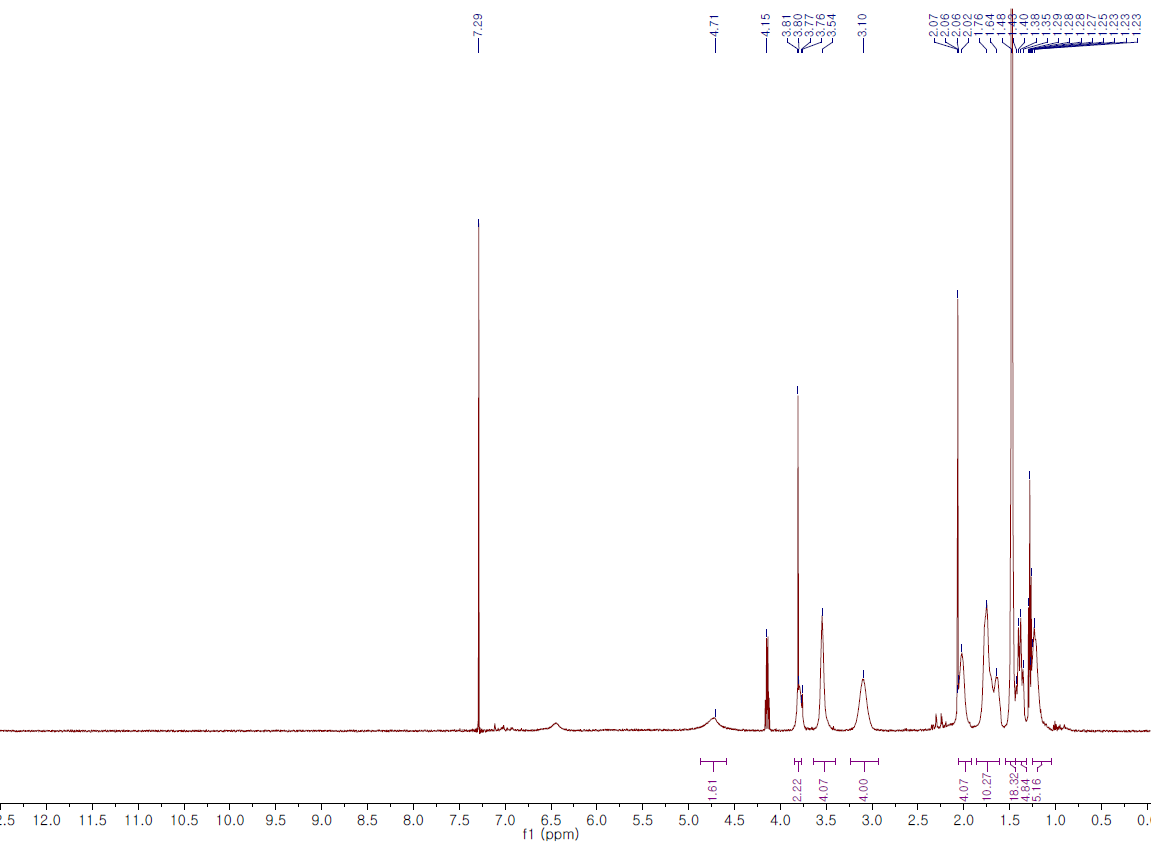


^1^H NMR spectrum of **9c** (CDCl_3_, 400 MHz)


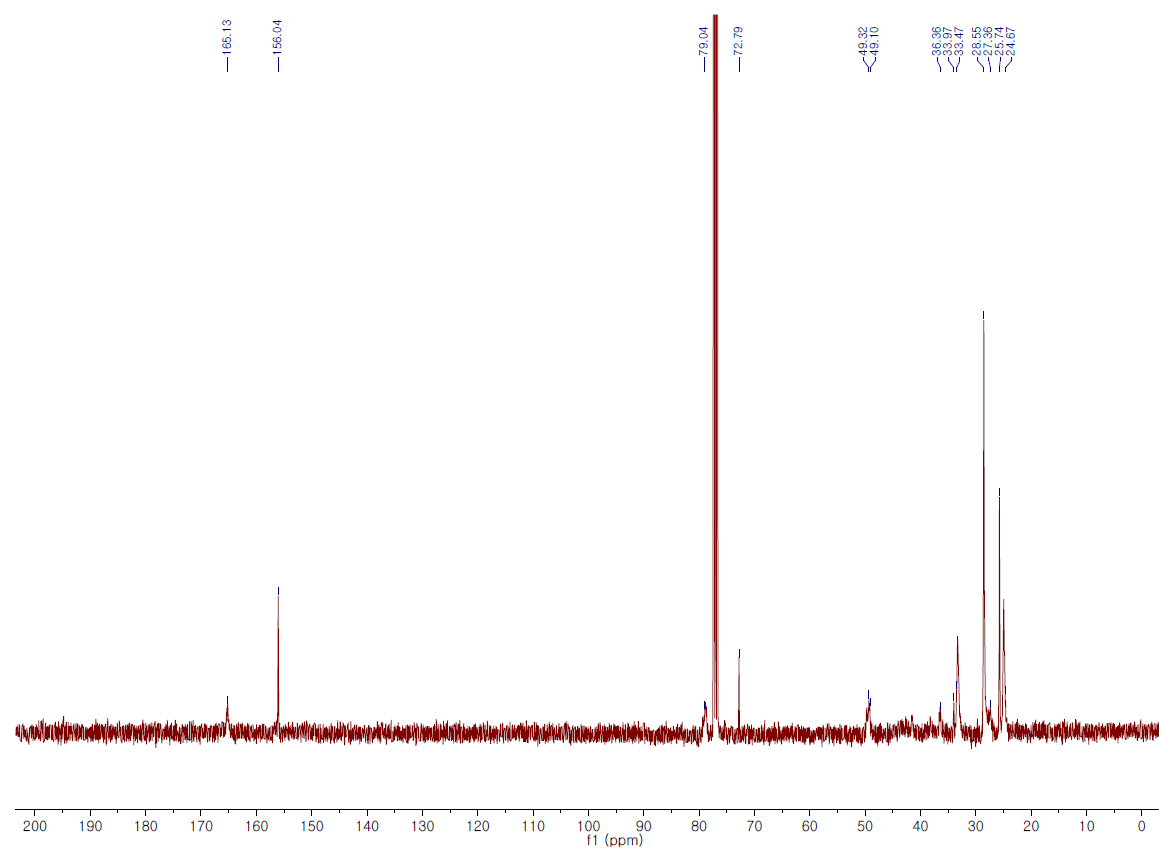


^13^C NMR spectrum of **9c** (CDCl_3_, 101 MHz)


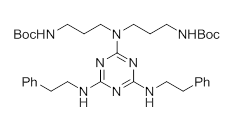

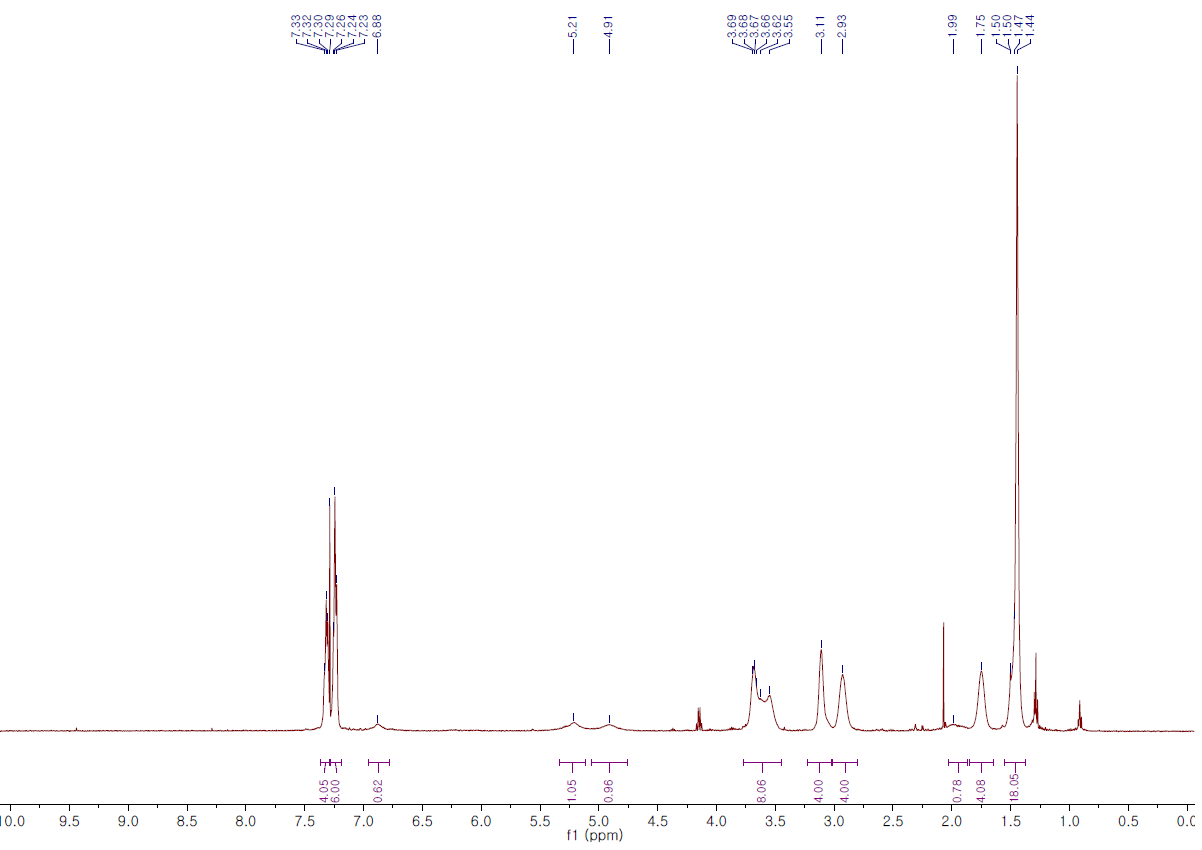


^1^H NMR spectrum of **9d** (CDCl_3_, 400 MHz)


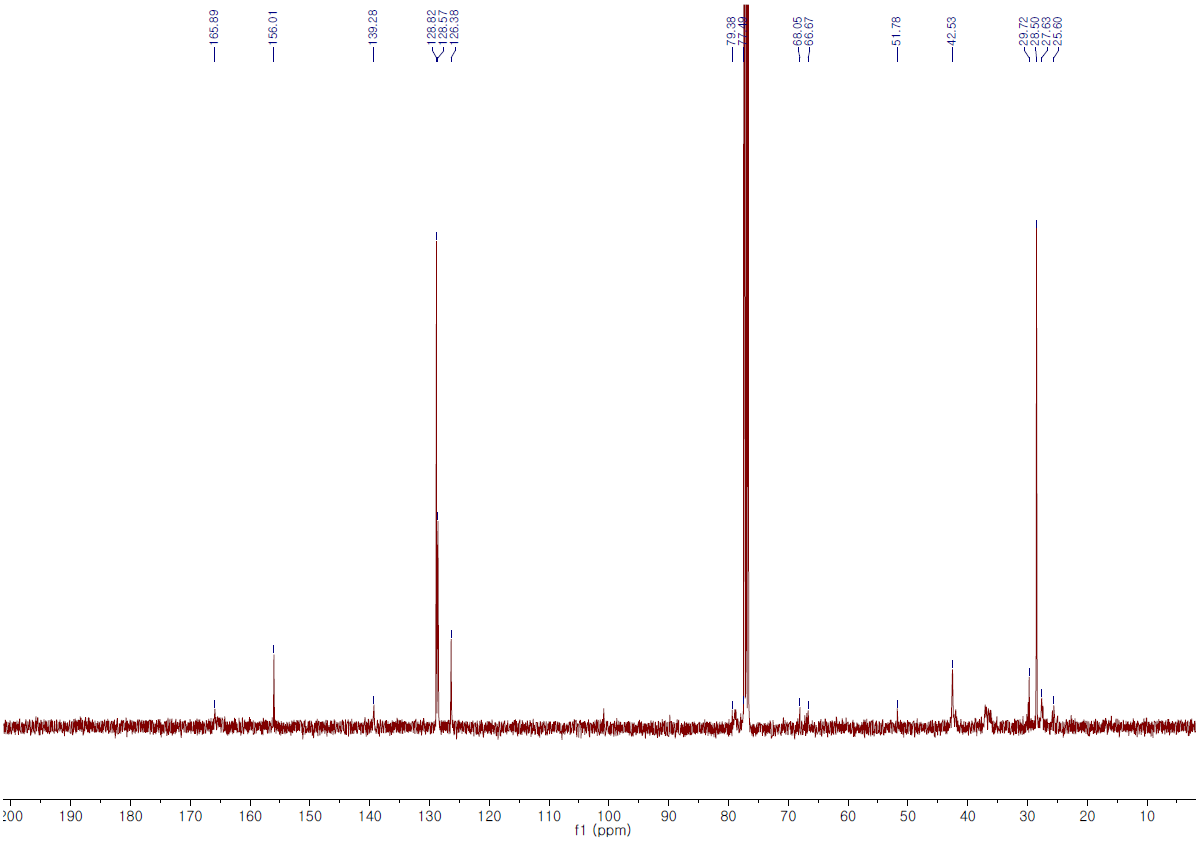


^13^H NMR spectrum of **9d** (CDCl_3_, 101 MHz)


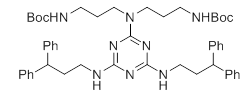

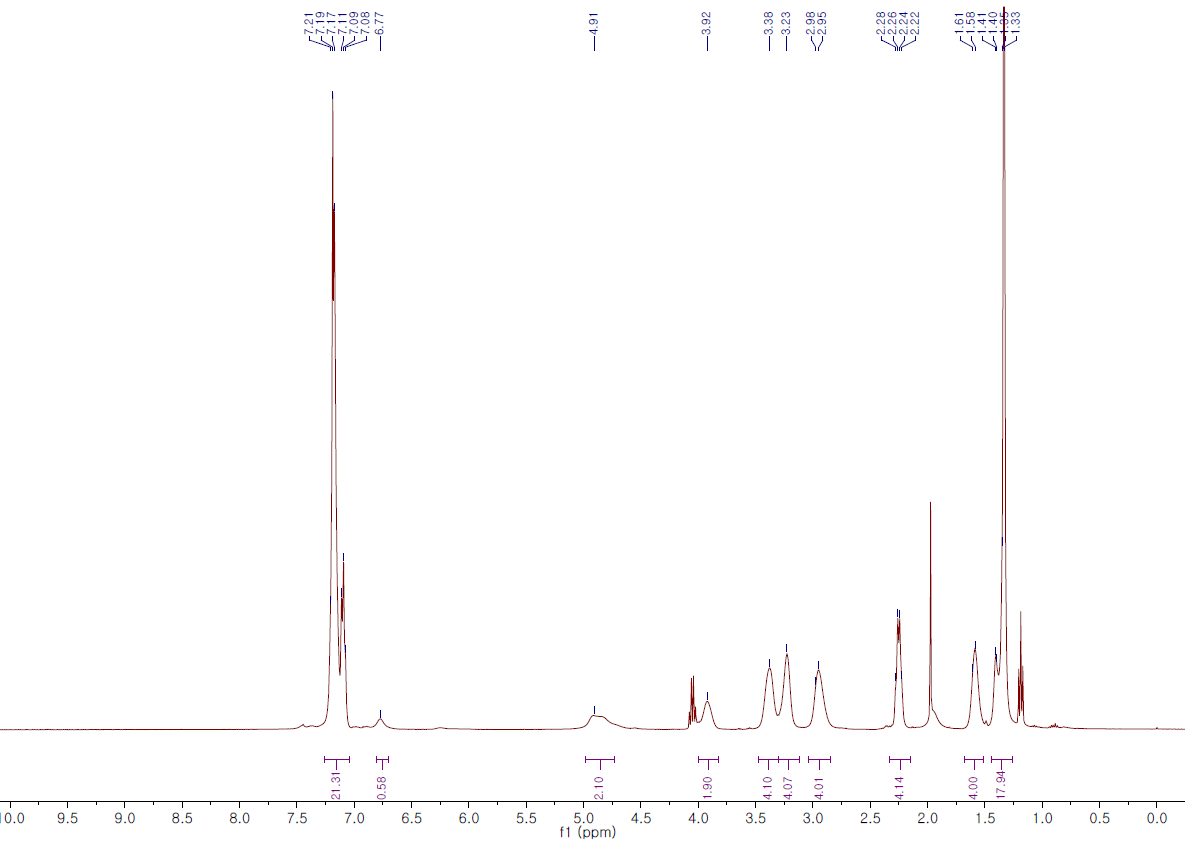


^1^H NMR spectrum of **9e** (CDCl_3_, 400 MHz)


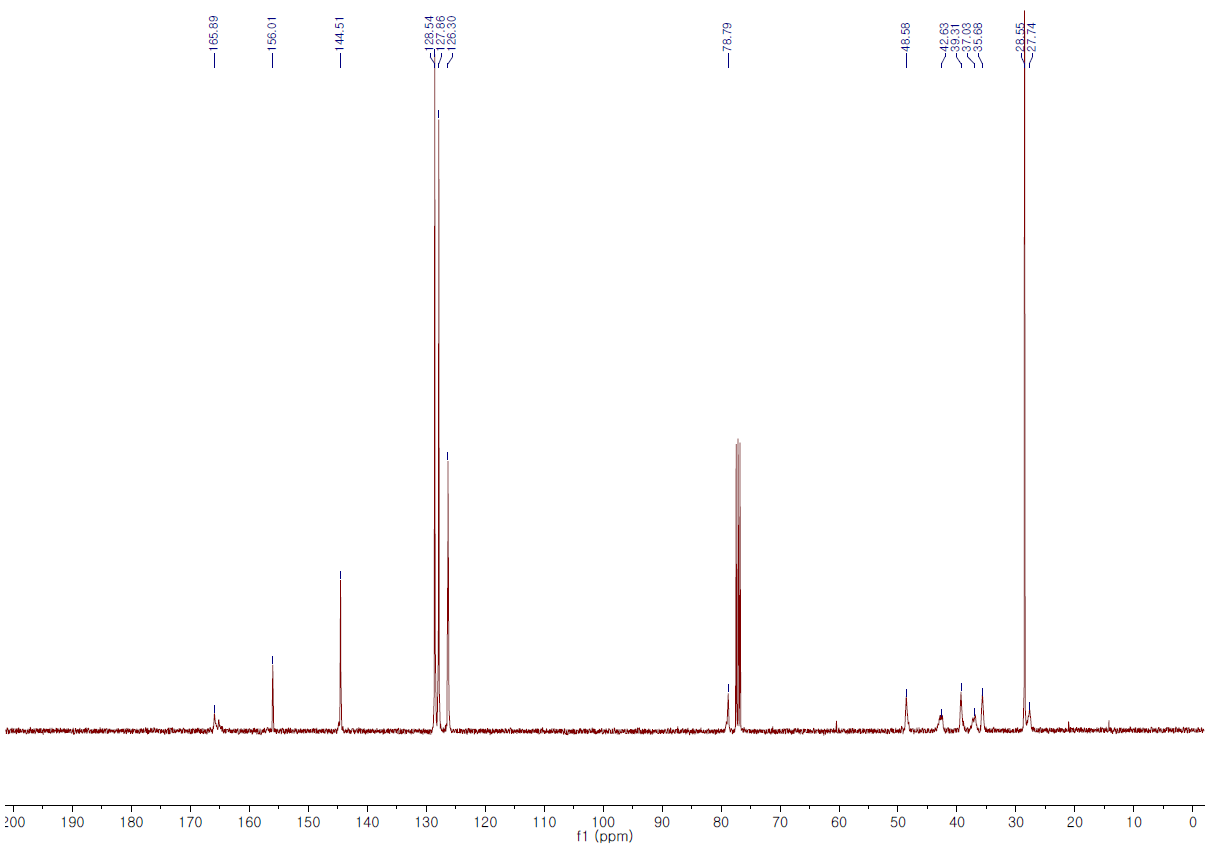


^13^C NMR spectrum of **9e** (CDCl_3_, 101 MHz)


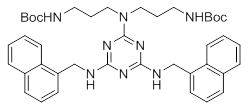

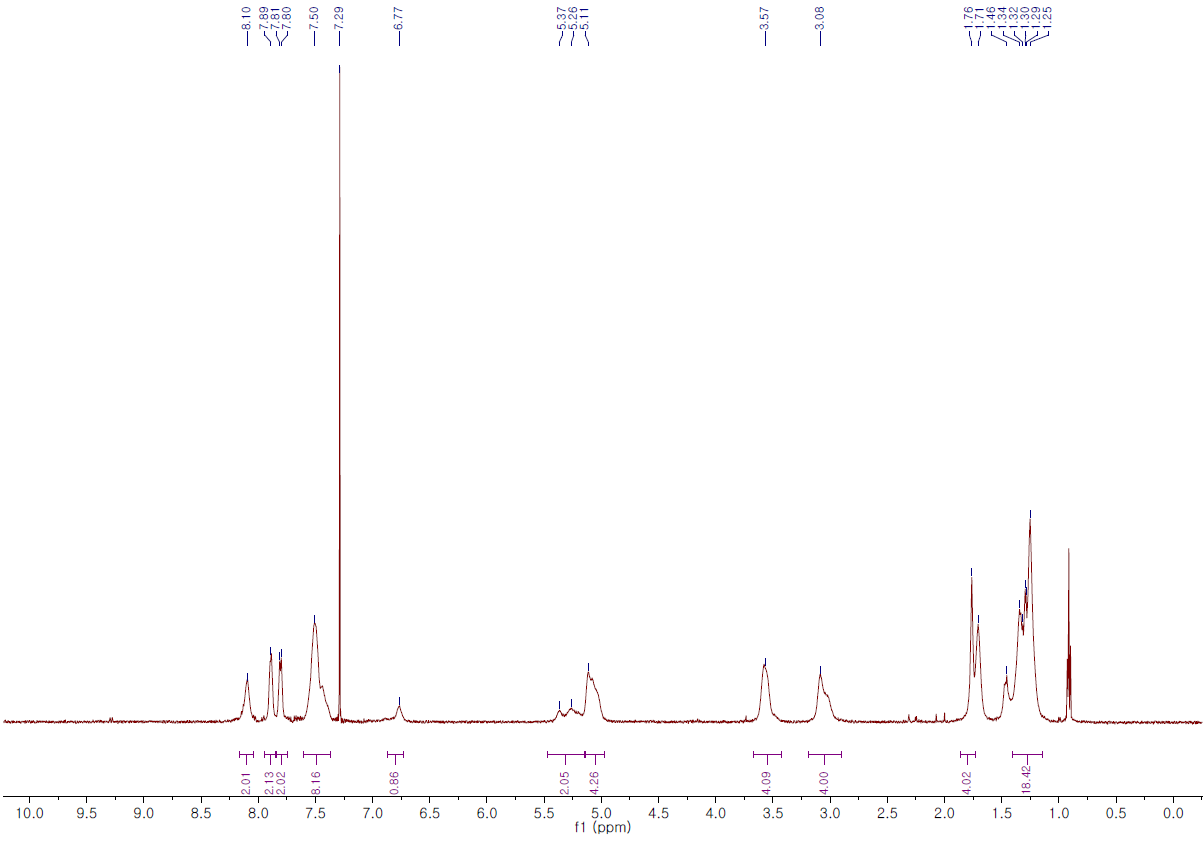


^1^H NMR spectrum of **9f** (CDCl_3_, 400 MHz)


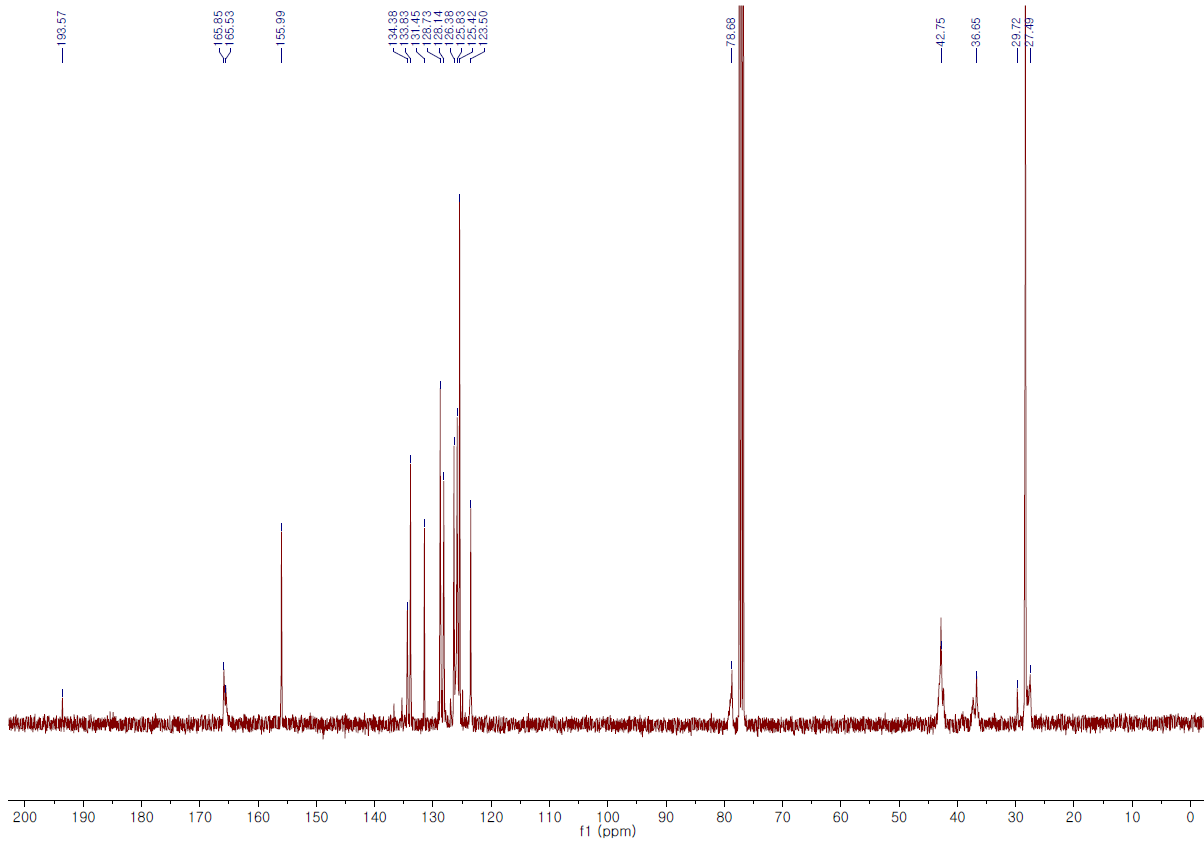


^13^C NMR spectrum of **9f** (CDCl_3_, 101 MHz)


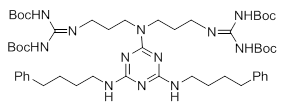

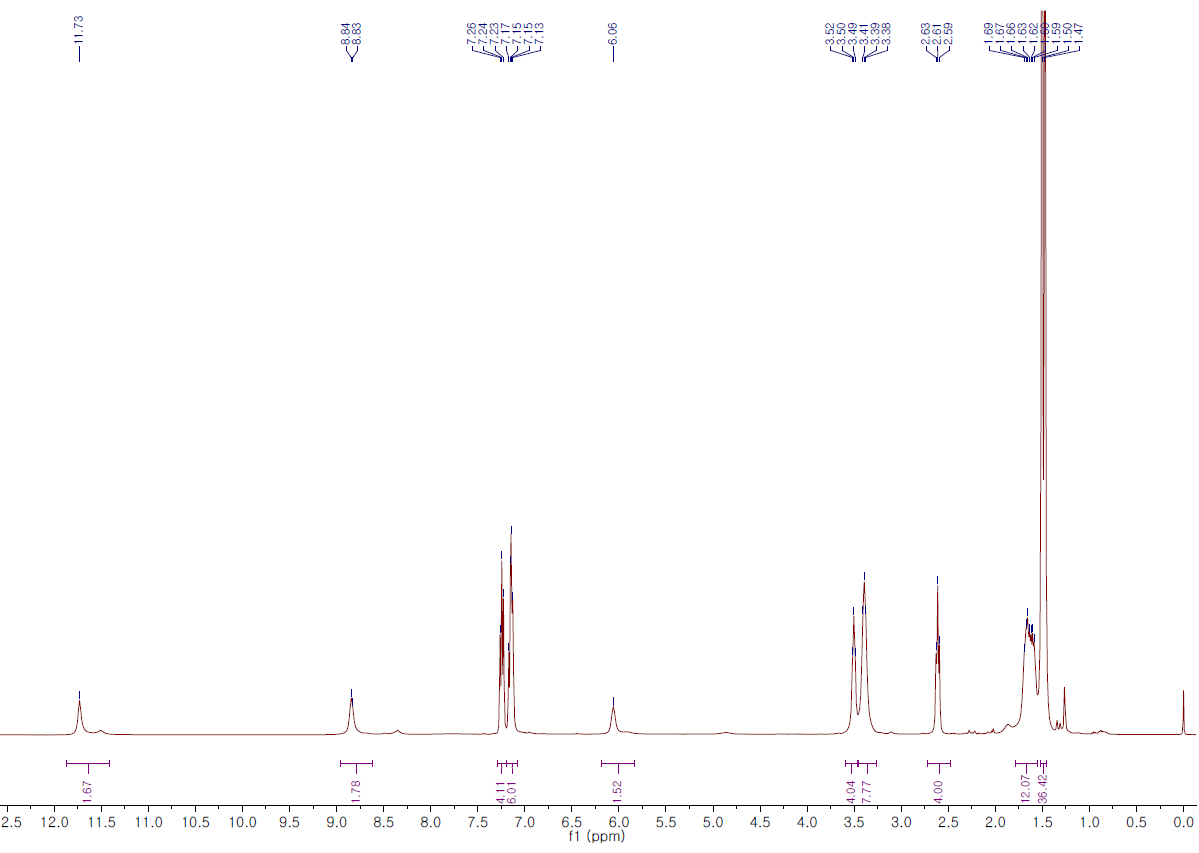


^1^H NMR spectrum of **10a** (CDCl_3_, 400 MHz)


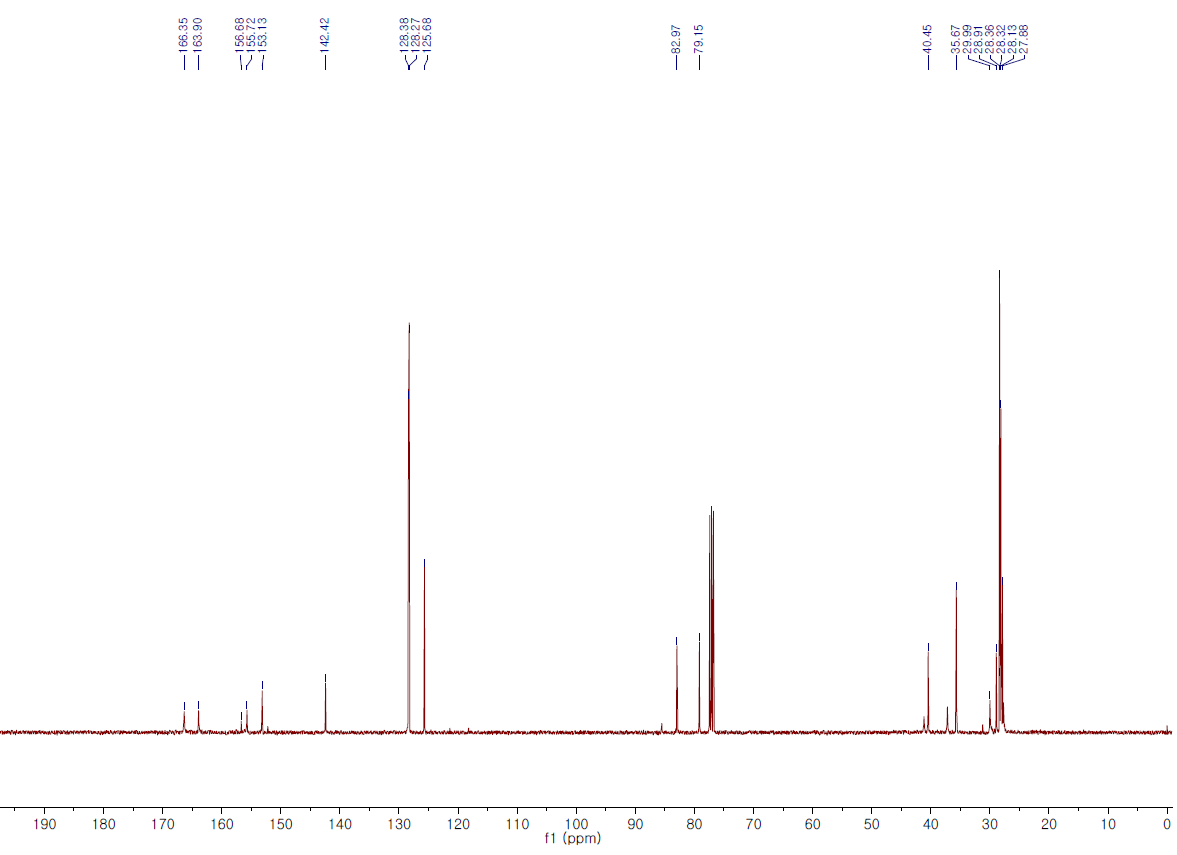


^1^H NMR spectrum of **10a** (CDCl_3_, 100 MHz)


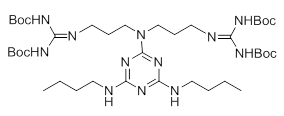

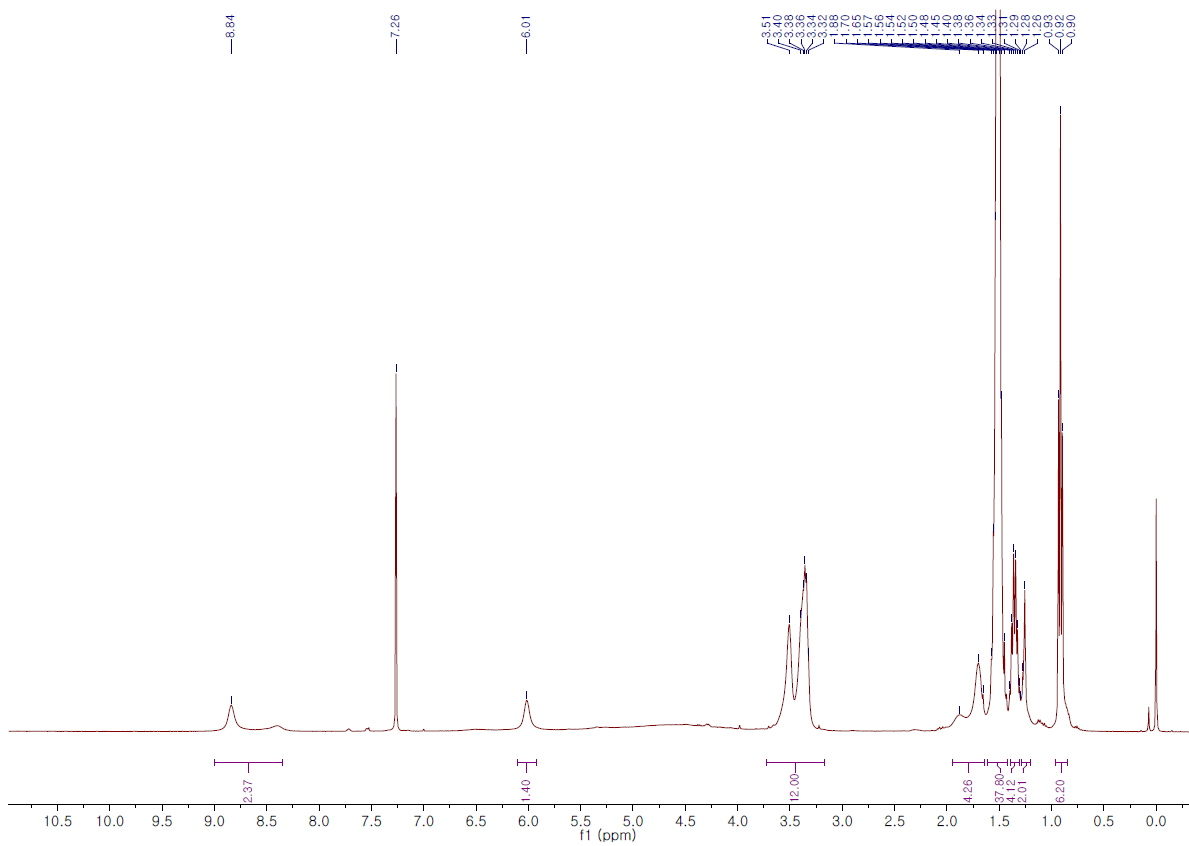


^1^H NMR spectrum of **10b** (CDCl_3_, 400 MHz)


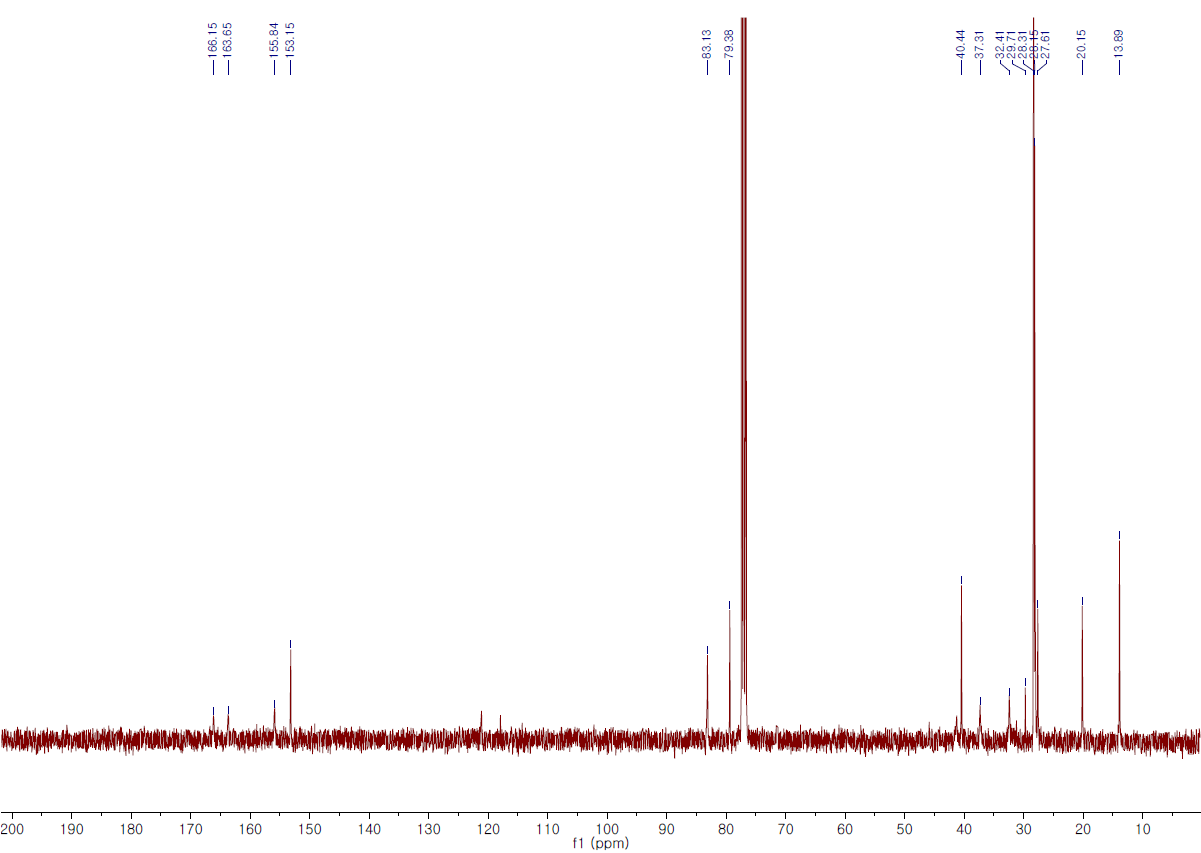


^1^H NMR spectrum of **10b** (CDCl_3_, 101 MHz)


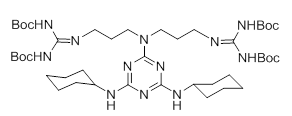

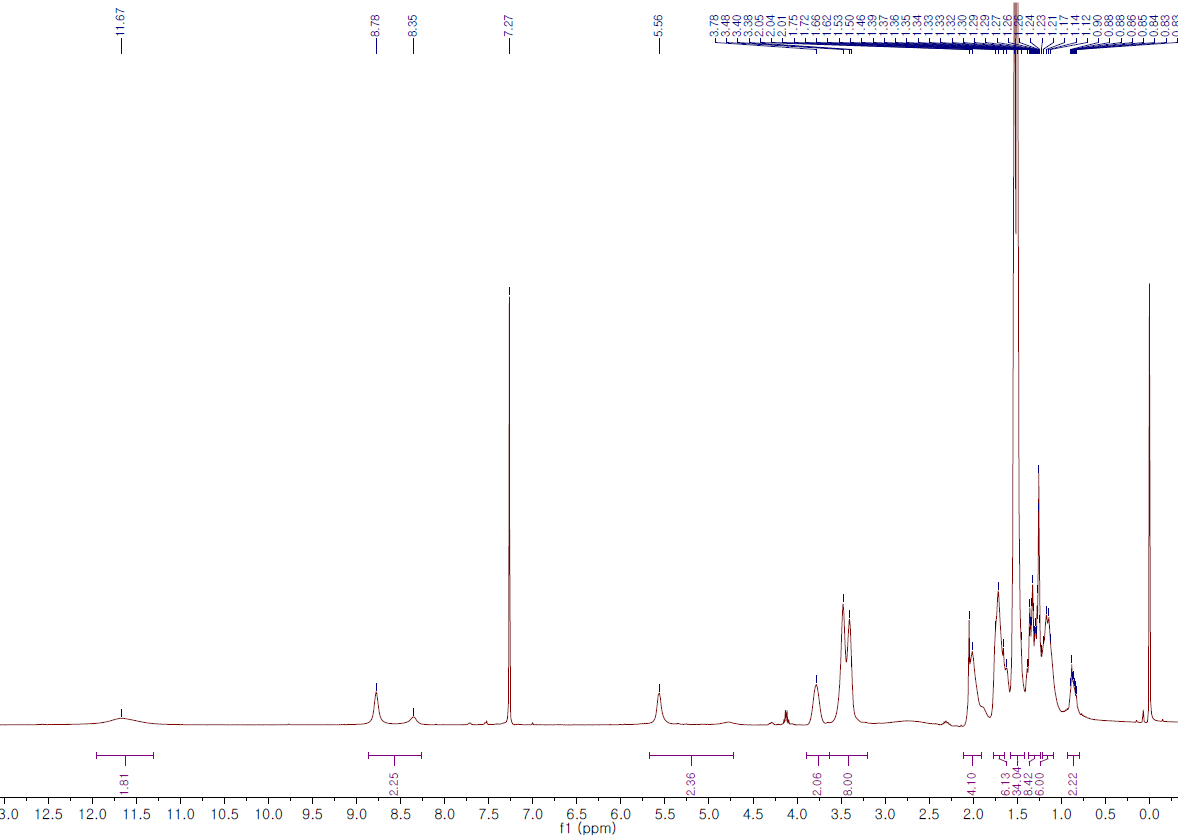


^1^H NMR spectrum of **10c** (CDCl_3_, 400 MHz)


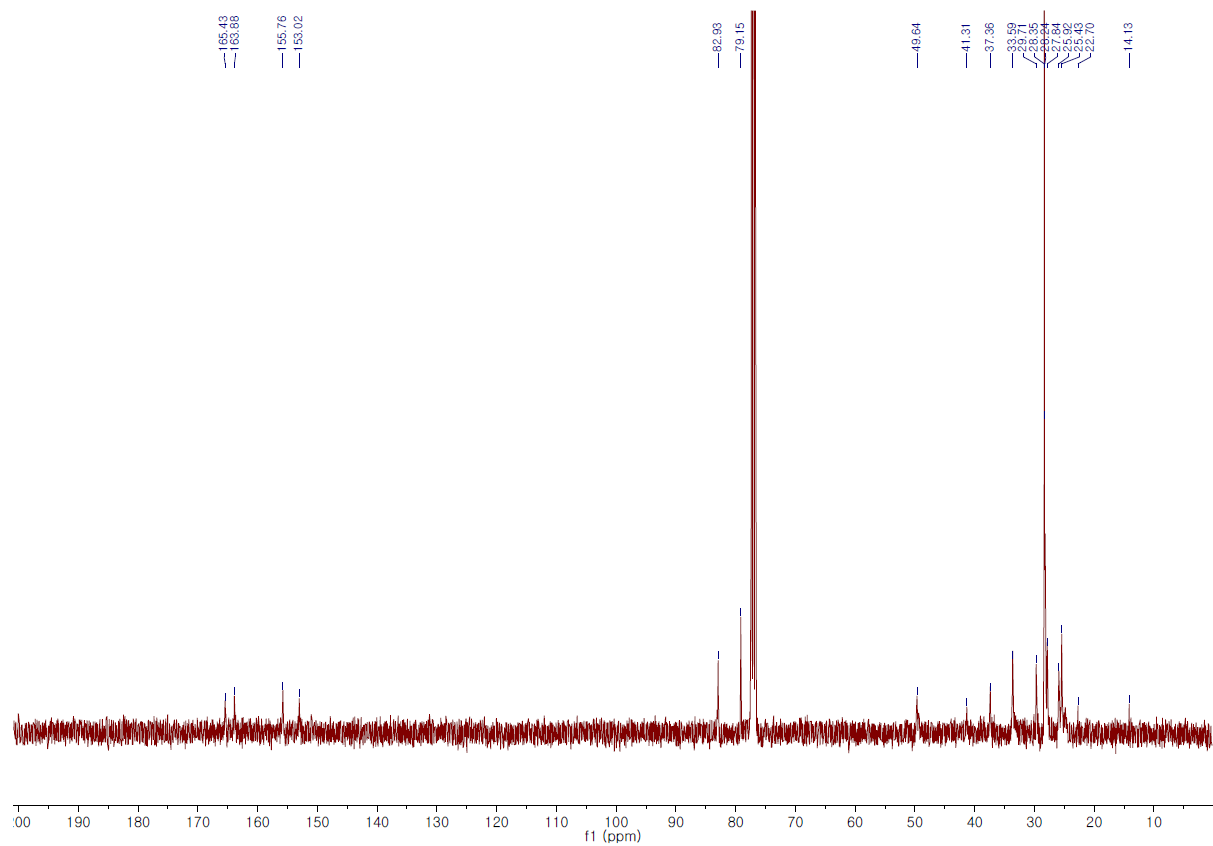


^1^H NMR spectrum of **10c** (CDCl_3_, 101 MHz)


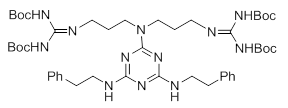

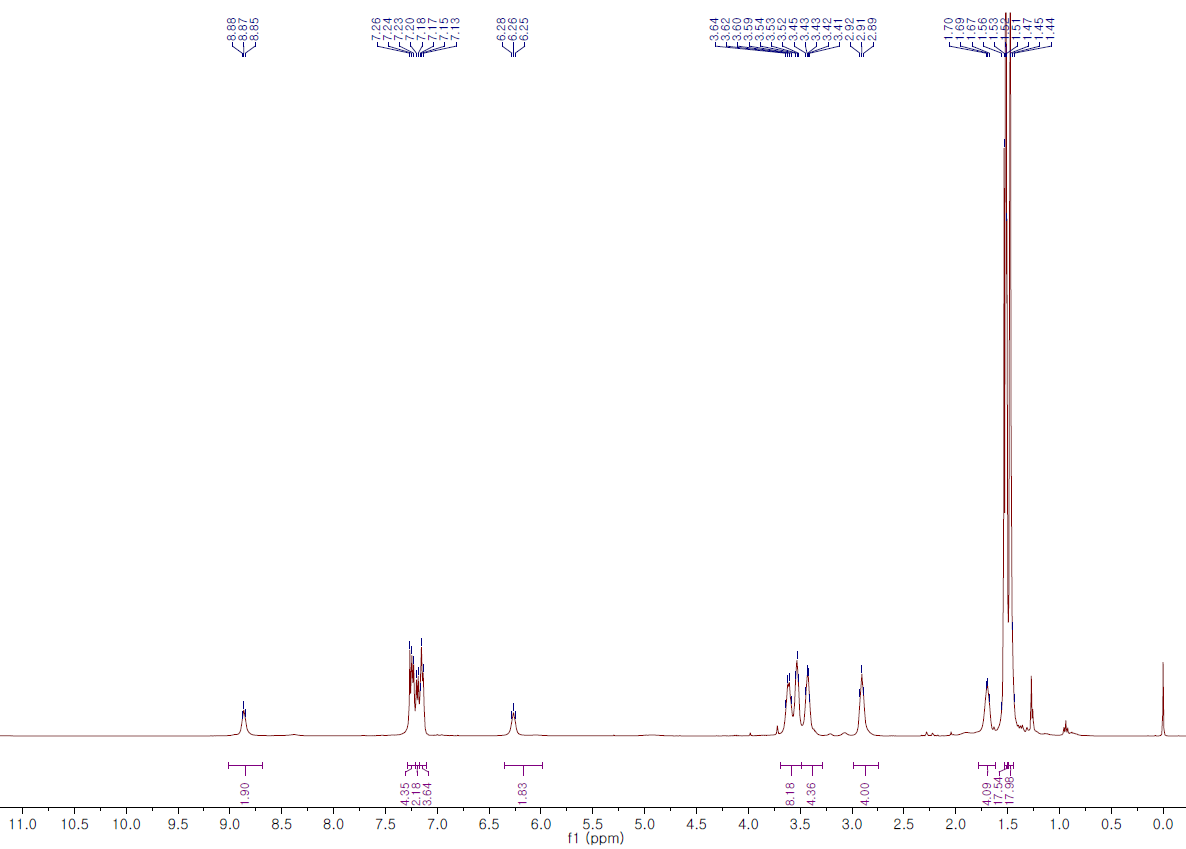


^1^H NMR spectrum of **10d** (CDCl_3_, 400 MHz)


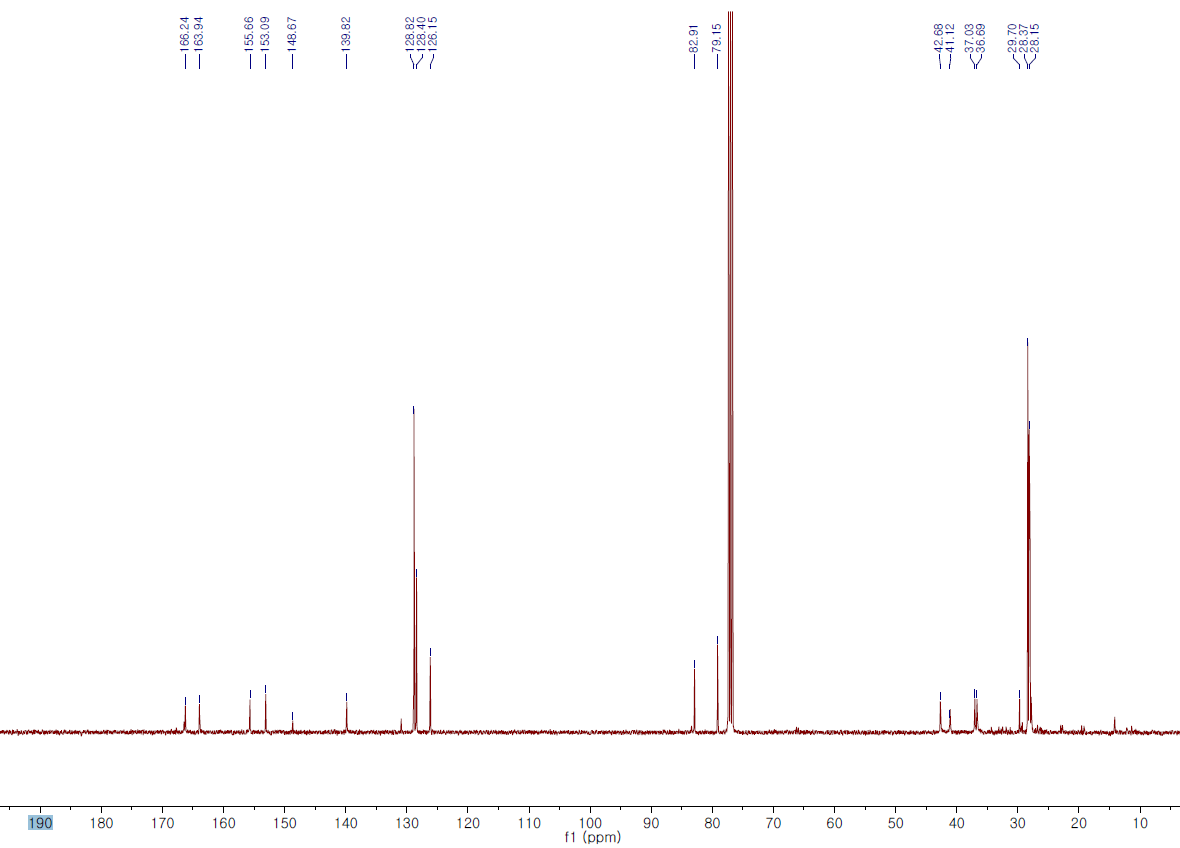


^13^C NMR spectrum of **10d** (CDCl_3_, 101 MHz)

^1^H NMR spectrum of **10e** (CDCl_3_, 400 MHz)

^13^C NMR spectrum of **10e** (CDCl_3_, 101 MHz)

^1^H NMR spectrum of **10f** (CDCl_3_, 400 MHz)

^13^C NMR spectrum of **10f** (CDCl_3_, 101 MHz)

^1^H NMR spectrum of **DL-1** (MeOD, 500 MHz)

^13^C NMR spectrum of **DL-1** (MeOD, 101 MHz)

^1^H NMR spectrum of **DL-2** (MeOD, 500 MHz)

^13^C NMR spectrum of **DL-2** (MeOD, 101 MHz)

^1^H NMR spectrum of **DL-3** (MeOD, 500 MHz)

^13^C NMR spectrum of **DL-3** (MeOD, 101 MHz)

^1^H NMR spectrum of **DL-4** (MeOD, 500 MHz)

^13^C NMR spectrum of **DL-4** (MeOD, 101 MHz)

^1^H NMR spectrum of **DL-5** (MeOD, 400 MHz)

^13^C NMR spectrum of **DL-5** (MeOD, 101 MHz)

^1^H NMR spectrum of **DL-6** (MeOD, 400 MHz)

^13^C NMR spectrum of **DL-6** (MeOD, 101 MHz)

^1^H NMR spectrum of **DG-1** (MeOD, 400 MHz)

^13^C NMR spectrum of **DG-1** (MeOD, 101 MHz)

^1^H NMR spectrum of **DG-2** (MeOD, 500 MHz)

^13^C NMR spectrum of **DG-2** (MeOD, 101 MHz)

^1^H NMR spectrum of **DG-3** (MeOD, 500 MHz)

^13^C NMR spectrum of **DG-3** (MeOD, 101 MHz)

^1^H NMR spectrum of **DG-4** (MeOD, 500 MHz)

^13^C NMR spectrum of **DG-4** (MeOD, 101 MHz)

^1^H NMR spectrum of **DG-5** (MeOD, 500 MHz)

^13^C NMR spectrum of **DG-5** (MeOD, 101 MHz)

^1^H NMR spectrum of **DG-6** (MeOD, 500 MHz)

^13^C NMR spectrum of **DG-6** (MeOD, 101 MHz)

^1^H NMR spectrum of **Dl-6-FITC** (MeOD, 400 MHz)

^13^C NMR spectrum of **DL-6-FITC** (MeOD, 101 MHz)
